# Changes in condom use among males who have sex with males (MSM): Measuring the effect of HIV prevention programme in Dhaka city

**DOI:** 10.1371/journal.pone.0236557

**Published:** 2020-07-24

**Authors:** Md. Masud Reza, AKM Masud Rana, Tasnim Azim, Ezazul Islam Chowdhury, Gorkey Gourab, Md. Sha Al Imran, Md. Aminul Islam, Sharful Islam Khan

**Affiliations:** 1 Programme for HIV and AIDS, Infectious Disease Division, International Centre for Diarrhoeal Disease Research, Bangladesh (icddr,b), Dhaka, Bangladesh; 2 Regional Advisor for Research Policy and Cooperation (RPC), Department of Communicable Diseases, WHO South-East Asia Regional Office, Delhi, India; 3 HIV/AIDS Program, Save the Children International, Dhaka, Bangladesh; National Institute for Communicable Disease (NICD), South Africa, SOUTH AFRICA

## Abstract

**Background:**

A systematic assessment was done to examine the effect of HIV interventions among MSM in Dhaka, Bangladesh. MSM were defined as males having sex with males but did not sell sex in the last year. MSM are hidden, marginalized and stigmatized population groups not only in Bangladesh but also globally. In 2010, HIV interventions for MSM were expanded in 40 districts of Bangladesh through 65 drop-in-centres (DICs) and peer outreach workers.

**Methods:**

Data from two surveys on MSM in Dhaka in 2010 (baseline) and 2013 (midline) were used to analyse the effect of ongoing HIV prevention services. Both surveys used time location sampling to randomly select MSM for risk behaviour interviews. Two outcome variables were considered; condom use in the last anal sex act and consistent condom use during anal sex in the last month. Univariate and multivariate logistic regression methods were used to determine factors associated with condom use.

**Results:**

Condom use significantly increased at the midline than baseline (p<0.001 for both). Multivariate analysis showed that having comprehensive knowledge of HIV and participation in HIV prevention programme were positively associated with both last time and consistent condom use. MSM who had comprehensive knowledge of HIV were 1.9 times (95% CI: 1.3–2.8, p = 0.002) and 2.1 times (95% CI: 1.4–3.2, p<0.001) more likely to use condoms than those who did not have comprehensive knowledge of HIV. The likelihood of using condoms among MSM was more than double at the midline than the baseline (p<0.01 for both). However, odds of condom use was significantly lower among those who perceived themselves to be at risk or were not able to assess their own risk of HIV.

**Conclusion:**

To sustain positive changes in HIV risk behaviours, HIV prevention programmes for MSM need to be continued and strengthened.

## Introduction

Males having Sex with Males (MSM) are at elevated risk of HIV infection in most countries around the world [1]. In the cities of Southern and South-eastern Asia, prevalence of HIV among MSM ranges from 6–29% [2] and 4–81% [3–12] of MSM received HIV prevention services. The hidden nature of MSM and the criminalisation of MSM behaviours in many countries makes accessing MSM for services a challenge [13] and in South Asia including Bangladesh and Pakistan, the Penal Code 377 is one such act that states “Whoever voluntarily has carnal intercourse against the order of nature with any man, woman or animal, shall be punished with imprisonment for life, or with imprisonment of either description for a term which may be extended to ten years, and shall also be liable to fine” [14, 15]. Furthermore, police can arrest persons without any warrant based on information provided by a third party who are involved with such offences [16]. Bail for this offence is at the discretion of the courts and can take up to two years to obtain [15].

In Bangladesh, HIV prevalence among MSM is still low. The last HIV serological surveillance conducted in 2015 among MSM in Dhaka city showed that the prevalence of HIV was only 0.3% and the prevalence of active syphilis was 1.5% [17]. The data also showed that, risky sexual behaviours were highly prevalent with 46% of MSM not using a condom in the last sex act and only 34–50% using condoms consistently with male sex partners during the last month [17]. Moreover, 13.5% of MSM reported that they were beaten and/or raped in the last year, only 10.6% ever accessed HIV testing services (HTS) and 35.5% had comprehensive knowledge of HIV [17]. Given these data there is concern that HIV may rise among MSM if prevention efforts are not strengthened.

In Bangladesh HIV prevention services for MSM started in 1997 [18] and since then services have expanded with support from various sources. In 2010, the estimated number of MSM was 110,581 [19] and the HIV prevention services expanded to 18,231 (16.5% of the estimated number) MSM in 40 districts of Bangladesh through 65 drop-in-centres (DICs). At the end of December 2012, in Dhaka city, ~1,000 (0.9% of the estimated number) received services through five DICs. The current HIV prevention services (from 2010 and onwards) to MSM build on the previous design of November 2009 [18] with a few differences. The services are implemented through static DICs and peer outreach workers in cruising spots where MSM congregate. The services include behaviour change communication (BCC), condom and lubricant promotion and distribution, HIV testing and services (HTS), referral for HIV positive MSM to care, support and treatment and management of sexually transmitted infections (STIs) including counselling. Each peer educator provides HIV prevention services to ~140 MSM at the cruising spots five days a week. To ensure availability of condoms and lubricants during holidays and weekends, condoms and lubricants are made available at fixed sites, known as depot holders (DH), in the localities frequented by MSM. Advocacy with community and law enforcement agencies are conducted locally to promote an enabling environment. There are provisions for legal support, if required. In addition, a participatory monitoring and evaluation (PM&E) is in place that has served to enhance the quality of the programme data and provide a better understanding of the underlying context and to ensure rapid action for improvement of programme performance [20].

Prior to the upscaling of HIV prevention programme for MSM in Bangladesh, a risk behaviour survey was conducted in Dhaka, the capital city in 2010 (referred to as the baseline survey) [21] and this was repeated later, in 2013 (referred to as the midline survey) [22]. The objective of the midline survey was to compare the progress of the HIV prevention programme in-terms of impact and outcome indicators, i.e. HIV prevalence and safer sex behaviours with MSM and transgender women (hijra) over time. Hijra or transgender women refer to those who identify themselves as belonging to a traditional hijra sub-culture [19]. Dhaka was selected for the baseline survey because of two reasons: i) at that time, 35,355 MSM [19] (32% of the total MSM in Bangladesh) were living in Dhaka city and ii) of 2,228 HIV positive cases that were detected from key populations (KPs) at risk of HIV from 2007–2013 in Bangladesh, mostly (19.2%) were detected in Dhaka [23].

Here we present an analysis of data obtained from the two surveys (baseline and midline) that was conducted to determine changes in key risk behaviours and correlates of condom use in MSM in Dhaka following implementation three years of HIV prevention services. Such evaluation of behavioural interventions is pertinent in generating evidence for outcome-oriented interventions [24, 25].

## Materials and methods

### Setting

Both the baseline 2010 and midline 2013 was conducted in Dhaka, the capital city of Bangladesh. Furthermore, in 2010, an assessment was conducted to estimate the size of MSM in Dhaka city before starting HIV prevention program for MSM. The data showed that the estimated size of MSM was 110,581 [19]. Based on the fund available, during the phase-1 of the project (2010–2012), 18,230 (16.0%) and the phase-2 (2013–2015), 22,448 (20.0%) MSM received HIV prevention services. This is to be mentioned that in Dhaka city, there was no HIV prevention services for MSM by any other agencies and since 2010, it is being running by a single donor. Male to male sex is still prohibited in Bangladesh and therefore, they are hidden and hard to reach. There are 9,379 MSM in Dhaka city [26] and only 17.7% (1,659; program data, Jul-Dec, 2019) received HIV prevention services. There is also a lack of research data on MSM population that can contribute to increase HIV prevention services. Therefore, the findings from this analysis are still important for program managers and policy makers.

### Study design

Both surveys (baseline in 2010 and midline in 2013) were conducted in Dhaka city and followed a similar cross-sectional design aimed at collecting HIV risk behavioural data from MSM 18 years and older [21, 22]. MSM were defined as males having sex with males but did not sell sex in the last month.

Standard guideline was followed to conduct both the baseline and midline surveys as suggested by USAID and DFID [27]. Time Location Sampling (TLS), which is a two-stage probability sampling method [27] was used in both surveys. Both in 2010 and 2013, a place to be defined as a ‘spot’ where at least 3 MSM were seen during mapping.

In 2010, social mapping was conducted in 211 places in Dhaka city. At least one MSM was seen during mapping in 160 places. To make the sampling frame of spots, which was the first stage of sampling, 126 places were filtered (considered as spots) from 160 places where at least 2 MSM were seen during mapping. This is to be noted that to be in the safe side we filtered the spots where at least 2 or more MSM were seen during mapping. Then from 126 spots, 114 spots were chosen using systematic random sampling technique.

In 2013, social mapping was conducted in 234 places in Dhaka city. At least one MSM was seen during mapping in 182 places. To make the sampling frame of spots, which was the first stage of sampling, 164 places were filtered (considered as spots) from 182 places where at least 2 MSM were seen during mapping. This is to be noted that to be in the safe side we filtered the spots where at least 2 or more MSM were seen during mapping. Then from 164 spots, 119 spots were chosen using systematic random sampling technique.

In both surveys, interviews were conducted randomly during a suitable time of day from 5 PM to 11 PM assuming a fixed 4 MSM to be interviewed from each spot, which was the second stage of sampling. Besides interviewing of MSM, the data collectors also collected information on the number of MSM available in a particular spot during 5 PM to 11 PM which referred to as seen during interview. This information was used in the calculation of sampling weights. Data were collected for the baseline and midline surveys between February-March, 2010 and January-February, 2013, respectively.

### Sample size calculation

Both in the baseline and midline, the sample sizes were calculated using a standard formula shown below [27].

$$n=D\frac{{\left\{z_{1-\text{α}}\sqrt{2\bar{p}(1-\bar{p})}+z_{1-\beta }\sqrt{p_{1}(1-p_{1})+p_{2}(1-p_{2})}\right\}}^{2}}{{\left(p_{2}-p_{1}\right)}^{2}}$$

In the above formula:

D = Design effect.

p1 = Estimated proportion of risk behaviour at the time of previous survey.

p2 = The target proportion at some future date, so that (p2-p1) is the magnitude of change that we want to be able to detect.

p(bar)=(p1+p2)/2

Z_1-α_ = The Z-score corresponding to desired level of significance = 1.645.

Z_1-β_ = The Z-score corresponding to desired level of power = 0.83.

In the baseline, two risk behavioural indicators from the last round of behavioural survey and surveillance (BSS) conducted in 2006–07 among MSM in the Dhaka were used [28]. These indicators were: condom use in the last anal sex act while buying sex from males (not transgender) and condom use in the last anal sex act while having sex with non-commercial male/transgender sex partners in the last month. The sample size was calculated to detect 11–14% (1-way change detectable) changes in these risk behaviours over time for the population group. In the calculation of sample size, we also used the inflation rates (percent of the population that is eligible to be considered for the indicators) from BSS 2006–07 for MSM population and a design effect of 2, 95% confidence level and 80% power. The calculated sample sizes were also adjusted for 5% non-response. For the first indicator, the calculated sample size was 439 and for the second indicator, 454.

In the midline 2013 [22], similar indicators and approach were used to calculate sample sizes. The estimates of the indicators were taken from baseline 2010 [21]. For the first indicator, the calculated sample size was 476 and for the second indicator, 387. Finally, the largest sample size was chosen for interview both in the baseline and midline.

### Survey tool

Face to face interviews were conducted using semi-structured questionnaires by experienced interviewers at public spots where some privacy could be assured [28–30]. The questionnaire included information on socio-demographic characteristics sexual history, sexual risk-behaviour, mobility, knowledge of male condoms and lubricants, knowledge of STIs and HIV, self-reported symptoms of STIs, knowledge and uptake of HTS, violence, HIV risk self-assessment and participation in HIV prevention programmes. Before each interview, verbal informed consent was obtained from the respondents. Because, same-sex sexual acts are criminalized in Bangladesh under Section 377 of the Penal Code and punishable by life imprisonment. Our experiences of working with MSM and transgender women (known as *hijra*) with such sensitive topic suggest that many of them do not like to disclose their identity (e.g. sexual orientation, involvement in sex trade) in written form due to the fear of identity disclosure. Structural barriers such as religious prohibition; criminalization of sex work and same-sex behaviour; and social stigma hinders getting written consent from them. Also, most often, requesting for written consent make them suspicious about the purpose of the study. This process not only influences their participation in the study but also their responses to the sensitive questions. Hence, we collected verbal consent from the research participants. Our trained research team members carefully obtained verbal informed consent and sought permission through verbal approval before each interview. The studies, findings of which were analysed in this manuscript, were approved by the Ethical Review Committee (ERC) at icddr,b.

### Measures

In this analysis, two key risk behaviours of MSM were considered as outcome variables. The first outcome variable was ‘used condom in the last sex act with any transactional/non-transactional males/hijra sex partners in the last month’ and the second outcome variable was ‘used condoms consistently in all anal sex acts with any transactional/non-transactional males/hijra sex partners in the last month’.

### Statistical analysis

A composite indicator was computed to measure the comprehensive knowledge of HIV who correctly identified two ways to prevent HIV and rejected three misconceptions regarding HIV transmission implies that 1) Can the risk of HIV transmission be reduced by having sex with only one uninfected partner who has no other partners?, 2) Can a person reduce the risk of getting HIV by using a condom, every time they have sex?, 3) Can a healthy-looking person have HIV?, 4) Can a person get HIV from mosquito bites? and 5) Can a person get HIV by sharing food with someone who is infected? [31]. Uptake of HTS was computed based on who received HIV testing as well as received the test result in the last 12 months [31]. Categorical variables were measured by percentage points and numerical variables by means along with 95% conficence intervals (CIs) for both. All variables between the two survey periods were compared using Chi-square statistics and any 5% was used as a level of significance. To identify the factors associated with using condom in the last sex act and using condoms consistently in the last month, bivariate analysis was carried out initially using univariate logistic regression models [32]. Thereafter, the net association of the factors associated with using condom in the last sex act and consistently with male/hijra sex partners, was determined by using multiple logistic regression models [32]. Factors that were significant at the 10% level in the bivariate analysis were included in the multivariate analysis [33, 34]. Results from the bivariate analysis were reported as unadjusted odds ratios (UOR) and from multivariate analysis as adjusted odds ratios (AOR). Before running multivariate analysis, pair-wise correlation coefficients were checked among the factors that were significant at p-value of <0.10 in the bivariate analysis [35]. In all analysis, appropriate sampling weights and clustering of observations were incorporated [27]. In this analysis, data from two-time points were appended. Data were analysed with Stata using complex survey design commands, Version 13.1 [36]. In the baseline, data were collected from 457 MSM and in the midline, from 487 MSM. The analysis was conducted among those who were 18 or above years of old. Finally, at both time points some of which were also married to women, a total of 457 MSM participated at the baseline were compared with 475 MSM at the midline. This is to be noted that after appending two data sets, sampling weights were modified [37] and then standard formula was used [27] to make it standardised so that in the analysis, the total number of observations become equal to the sum of sampling weights. Hence, for the sake of completeness in the analysis, all results in the manuscript are reported based on the modified sampling weights. After incorporating clustering of observations in the analysis, the calculation of standard error of estimates was based on cluster sampling design that was been automatically modified by Stata and based on that 95% confidence intervals were calculated in the univariate, bivariate and multivariate analyses.

## Results

### Comparison between the baseline and midline surveys

Comparisons between the two surveys are shown in Table 1. There were no differences in sociodemographic characteristics including age, years of schooling, marital status however, income significantly increased at the midline than the baseline (p<0.001) (Table 1). This is to be mentioned that 30–32% of MSM in both time points were married that indicates they were bisexual.

**Table 1:** Comparison of socio-demographics between baseline and midline.

Variables	Baseline (2010)	Midline (2013)	Comparison
	n, Col% (95% CI)	n, Col% (95% CI)	(p-value)
	(N = 457)	(N = 475)	
Age			
18–24 years	183, 39.4 (34.1–45.0)	197, 40.1 (35.1–45.3)	NS
>= 24 years	274, 60.6 (55.0–65.9)	278, 59.9 (54.7–64.9)	NS
Years of schooling^§^			
0	36, 7.9 (5.6–11.1)	44, 9.4 (5.8–14.9)	NS
1–5	120, 26.6 (21.4–32.6)	107, 23.1 (18.9–28.0)	NS
6–10	214, 46.0 (40.9–51.3)	183, 38.5 (33.5–43.8)	NS
>10	86, 19.4 (15.0–24.7)	139, 28.9 (23.7–34.8)	NS
Income in the last month (USD)*			
<= 91	296, 66.3 (60.4–71.7)	195, 40.1 (35.4–45.0)	<0.001
>91	161, 33.7 (28.3–39.6)	280, 59.9 (55.0–64.6)	<0.001
Current marital status			
Unmarried	295, 65.0 (59.9–69.8)	323, 67.0 (61.9–71.7)	NS
Married	144, 31.8 (27.2–36.9)	136, 29.6 (25.2–34.3)	NS
Divorced/Separated/Widower	18, 3.1 (1.9–5.1)	16, 3.5 (2.1–5.6)	NS

*Based on median value (1 USD = 91.0 BDT); NS indicates a non-significant comparison at the 5% level.

^§^ N = 456, one observation was missing in 2010 and N = 473, 2 observations were missing in 2013.

Condom use (both last time and consistent) significantly increased at the midline compared to the baseline (p<0.001 for both, Table 2). Risk perception changed significantly between the two time points; significantly more MSM at the midline versus the baseline survey did not consider themselves to be at risk of HIV (63%, 95% CI: 58.1–67.6 and 43%, 95% CI: 36.8–49.4; p<0.001) respectively. At the same time, fewer participants were not able to assess their own risk of HIV with 34.3% (95% CI: 27.9–41.3) at the baseline to 11.3% (95% CI: 7.7–16.2; p<0.001) at the midline. Two questions were asked to assess the knowledge of HIV prevention and three questions were asked to assess knowledge of HIV transmission. Use of condom (properly and consistently) as a mode of HIV prevention was reported by 82.4% (95% CI: 78.0–86.1) at the baseline and 88.5% (95% CI: 84.0–91.9) at the midline. At the baseline, 66.2% (95% CI: 61.2–70.8) and at the midline, 64.2% (95% CI: 58.1–69.9) mentioned avoiding multiple sex partners to prevent HIV. At both time points, almost half of the MSM rejected the misconception that HIV can be transmitted by mosquito bites and a little more than half rejected the misconception that HIV can be transmitted by sharing food with HIV infected persons. More MSM at the midline compared to the baseline rejected the misconception that one can tell by looking at someone whether he/she is infected with HIV (57.6%, 95% CI: 51.6–63.3 vs. 78.2%, 95% CI: 73.1–82.5; p<0.001). Only 23.5% (95% CI: 18.9–29.0) at the baseline and 26.6% (95% CI: 21.6–32.3) at the midline had comprehensive knowledge of HIV and no difference was observed.

**Table 2:** Comparison of sexual risk, vulnerabilities and programmatic variables between baseline and midline.

Variables	Baseline (2010)	Midline (2013)	Comparison
	n, Col% (95% CI)	n, Col% (95% CI)	(p-value)
	(N = 457)	(N = 475)	
Age at first sex (anal/vaginal)			
≤12 years	31, 7.7 (5.1–11.5)	70, 14.8 (11.3–19.3)	<0.01
13–19 years	373, 79.6 (74.2–84.0)	356, 75.4 (70.5–79.6)	NS
≥20 years	53, 12.8 (9.7–16.6)	49, 9.8 (7.2–13.2)	NS
Had anal sex with males/hijra in the last month	342, 74.7 (69.3–79.4)	400, 84.7 (80.0–88.5)	<0.01
Used condom in the last anal sex act with a male in the last month (denominator is who had anal sex with a male in the last month)	92, 24.8 (20.3–29.8) N = 342	197, 48.6 (42.4–54.7) N = 400	<0.001
Used condom consistently during all anal sex acts with males in the last month (denominator is who had anal sex with males in the last month)	61, 16.7 (12.7–21.6) N = 342	143, 35.1 (29.4–41.2) N = 400	<0.001
Reported at least one STI symptoms in the last year	80, 17.7 (13.4–23.2)	62, 13.3 (10.3–17.1)	NS
Mentioned condom use (correctly and consistently in any type of sex) as a mode of prevention	377, 82.4 (78.0–86.1)	417, 88.5 (84.0–91.9)	NS
Mentioned avoiding multiple sex partners as a mode of prevention	293, 66.2 (61.2–70.8) N = 448^¶^	305, 64.2 (58.1–69.9)	NS
Rejected misconception that HIV can be transmitted by mosquito bites	228, 50.6 (44.9–56.2) N = 450^§^	227, 46.7 (40.2–53.3)	NS
Rejected misconception that HIV can be transmitted by sharing food with an HIV infected person	261, 59.3 (53.5–64.8) N = 450^§^	265, 54.7 (48.1–61.2)	NS
Rejected misconception that one can tell by looking at someone whether he/she is infected with HIV	266, 57.6 (51.6–63.3) N = 448^¶^	371, 78.2 (73.1–82.5)	<0.001
Had comprehensive knowledge of HIV	108, 23.5 (18.9–29.0)	130, 26.6 (21.6–32.3)	NS
HIV risk perception^Ѳ^			
At risk (high or medium)	114, 22.7 (18.4–27.7)	128, 25.7 (21.1–31.0)	NS
Not at risk	192, 43.0 (36.8–49.4)	296, 63.0 (58.1–67.6)	<0.001
Not able to assess	146, 34.3 (27.9–41.3)	51, 11.3 (7.7–16.2)	<0.001

NS indicates a non-significant comparison at the 5% level.

^¶^9 observations were missing.

^§^7 observations were missing.

^Ѳ^N = 452, 5 observations were missing at baseline.

### Factors associated with condom use in the last sex act and consistently with a male/hijra sex partner in the last month

In the bivariate analysis, condom use in the last sex act was significantly associated (p<0.10) with income in the last month, having STI symptoms in the last year, HIV risk perception, having comprehensive knowledge of HIV and year of survey (Table 3). In the multivariate analysis, HIV risk perception, having comprehensive knowledge of HIV, having STI symptoms in the last year and year of survey retained significance at p<0.05 (Table 3). The likelihood of using a condom in the last sex act was significantly lower among those who perceived themselves to be at risk (high or medium) of HIV or were not able to assess their own risk compared to those who perceived themselves to be at no risk (p<0.05 for both). Concomitantly, comprehensive knowledge of HIV was positively associated with last time condom use (AOR: 1.9, 95% CI: 1.3–2.8, p = 0.002). Having a symptom of STI in the last year was negatively associated with last time condom use (AOR: 0.5, 95% CI: 0.4–0.9, p = 0.009). The year of intervention (2013) showed a positive impact on condom use in the last sex act (AOR: 2.3, 95% CI: 1.6–3.3, p<0.001).

**Table 3:** Factors associated with condom use in the last sex act.

Factors	Bivariate analysis				Multivariate analysis	
	Yes	No	UOR (95% CI)	p-value	AOR (95% CI)	p-value
	n/N (Row %)	n/N (Row %)				
Age						
18–24 years	115/296 (36.6)	181/296 (63.4)	0.9 (0.7–1.3)	NS	--	--
>24 years (RC)	174/446 (38.3)	272/446 (61.7)	1.0	--		
Years of schooling						
0 (RC)	20/67 (31.0)	47/67 (69.0)	1.0	--	--	--
1–5	58/164 (35.1)	106/164 (64.9)	1.2 (0.6–2.3)	NS		
6–10	123/319 (36.7)	196/319 (63.3)	1.3 (0.7–2.5)	NS		
>10	88/190 (44.1)	102/190 (55.9)	1.8 (0.9–3.4)	NS		
Income in the last month (USD)						
<= 91	120/375 (31.2)	255/375 (68.8)	1.0	--	1.0	--
>91	169/367 (44.3)	198/367 (55.7)	1.8 (1.3–2.4)	0.001	1.4 (1.0–1.9)	NS
Current marital status						
Unmarried (RC)	201/496 (38.9)	295/496 (61.1)	1.0	--	--	--
Married	80/217 (35.9)	137/217 (64.1)	0.9 (0.6–1.3)	NS		
Divorced/Separated/Widower	8/29 (29.4)	21/29 (70.6)	0.7 (0.3–1.6)	NS		
Age at first sex (anal/vaginal)						
≤12 years (RC)	31/83 (36.1)	52/83 (63.9)	1.0	--	--	--
13–19 years	230/581 (38.3)	351/581 (61.7)	1.1 (0.7–1.9)	NS		
≥20 years	28/78 (35.1)	50/78 (64.9)	1.0 (0.4–2.1)	NS		
HIV risk perception						
At risk (medium or high)	75/206 (34.8)	131/206 (65.2)	0.6 (0.4–0.9)	0.009	0.7 (0.4–1.0)	0.04
Not at risk (RC)	185/378 (47.2)	193/378 (52.8)	1.0	--	1.0	--
Not able to assess	28/154 (18.1)	126/154 (81.9)	0.2 (0.1–0.4)	<0.001	0.4 (0.2–0.6)	0.001
Reported at least one STI symptoms in the last year	32/120 (24.6)	88/120 (75.4)	0.5 (0.3–0.8)	0.002	0.5 (0.4–0.9)	0.009
Had comprehensive knowledge of HIV	108/204 (50.7)	96/204 (49.3)	2.1 (1.4–3.1)	<0.001	1.9 (1.3–2.8)	0.002
Year of survey						
2010 (baseline) (RC)	92/342 (24.8)	250/342 (75.2)	1.0	--	1.0	--
2013 (midline)	197/400 (48.6)	203/400 (51.4)	2.9 (2.0–4.1)	<0.001	2.3 (1.6–3.3)	<0.001

In the bivariate analysis NS indicates a non-significant result at the 10% level and in the multivariate analysis, at the 5% level; RC = reference category.

Consistent use of condoms was significantly lower among those who perceived themselves to be at risk (AOR: 0.4, 95% CI: 0.3–0.7, p<0.001) or were not able to assess their own risk of HIV compared to those who perceived themselves to be at no risk (AOR: 0.3, 95% CI: 0.2–0.5, p<0.001, Table 4). Significant positive associations with consistent condom use were observed with having comprehensive knowledge of HIV (AOR: 2.1, 95% CI: 1.4–3.2, p<0.001) and year of survey (AOR: 2.1, 95% CI: 1.4–3.4, p = 0.001).

**Table 4:** Factors associated with consistent use of condoms.

Factors	Bivariate analysis				Multivariate analysis	
	Yes	No	UOR (95% CI)	p-value	AOR (95% CI)	p-value
	n/N (Row %)	n/N (Row %)				
Age						
18–24 years	80/296 (25.3)	216/296 (74.7)	0.9 (0.6–1.3)	NS	--	--
>24 years (RC)	124/446 (27.5)	322/446 (72.5)	1.0	--		
Years of schooling						
0 (RC)	15/67 (23.1)	52/67 (76.9)	1.0	--	1.0	--
1–5	41/164 (23.0)	123/164 (77.0)	1.0 (0.5–2.0)	NS	1.0 (0.5–2.0)	NS
6–10	78/319 (24.1)	241/319 (75.9)	1.1 (0.5–2.1)	NS	1.0 (0.5–2.0)	NS
>10	70/190 (35.6)	120/190 (64.4)	1.8 (0.9–3.6)	0.08	1.0 (0.5–2.1)	NS
Income in the last month (USD)						
<= 91	83/375 (21.5)	292/375 (78.5)	1.0	--	1.0	--
>91	121/367 (31.9)	246/367 (68.1)	1.7 (1.2–2.4)	0.003	1.3 (0.9–1.9)	NS
Current marital status						
Unmarried (RC)	138/496 (26.7)	358/496 (73.3)	1.0	--	--	--
Married	60/217 (27.0)	157/217 (73.0)	1.0 (0.7–1.5)	NS		
Divorced/Separated/Widower	6/29 (23.7)	23/29 (76.3)	0.9 (0.3–2.3)	NS		
Age at first sex (anal/vaginal)						
≤12 years (RC)	20/83 (22.6)	63/83 (77.4)	1.0	--	--	--
13–19 years	164/581 (27.2)	417/581 (72.8)	1.3 (0.7–2.3)	NS		
≥20 years	20/78 (27.3)	58/78 (72.7)	1.3 (0.6–3.0)	NS		
HIV risk perception						
At risk (medium or high)	43/206 (19.6)	163/206 (80.4)	0.4 (0.3–0.6)	<0.001	0.4 (0.3–0.7)	<0.001
Not at risk (RC)	145/378 (36.9)	233/378 (63.1)	1.0	--	1.0	--
Not able to assess	15/154 (10.2)	139/154 89.8)	0.2 (0.1–0.4)	<0.001	0.3 (0.2–0.5)	<0.001
Reported at least one STI symptoms in the last year	21/120 (16.1)	99/120 (83.9)	0.5 (0.3–0.8)	0.008	0.6 (0.3–1.0)	NS
Had comprehensive knowledge of HIV	84/204 (39.9)	120/204 (60.1)	2.4 (1.6–3.5)	<0.001	2.1 (1.4–3.2)	<0.001
Year of survey						
2010 (baseline) (RC)	92/342 (24.8)	250/342 (75.2)	1.0	--	1.0	--
2013 (midline)	197/400 (48.6)	203/400 (51.4)	2.7 (1.8–4.1)	<0.001	2.1 (1.4–3.4)	0.001

In the bivariate analysis NS indicates a non-significant result at the 10% level and in the multivariate analysis, at the 5% level; RC = reference category.

## Discussion

Condom use, especially consistent condom use, is a cornerstone in the behavioural prevention programme against sexual transmission of HIV [38]. Condom use can be improved with effective programmes as has been shown in several cohort studies in China, Vietnam and Thailand [39–41] and in randomised control trials [42–44] in India, China, Russia and Hungary. The analysis presented here shows that condom use increased following three years of programming suggesting that the prevention programme for MSM in Dhaka had a positive effect in promoting safer sexual behaviours. These findings are consistent with other studies conducted in various countries globally. For example, similar findings were observed among MSM in Andhra Pradesh, Maharashtra and Tamil Nadu in India where HIV prevention programme had a positive impact on increasing condom use [45–47] as was the case in Anhui and Sichuan Provinces in China [48, 49] and in Senegal [50].

Although condom use increased after three years of intervention, the level of improvement is not sufficient to prevent a future HIV epidemic as half of the interviewed MSM still did not use a condom in the last sex act and about three in every five were not using condoms consistently. It is fortunate that HIV prevalence is still low among MSM in Bangladesh but recent data on the number of reported cases of HIV shows a consistent annual rise and MSM show the greatest rise in numbers after people who inject drugs [51]. Moreover, reports of STI symptoms within the last year did not decline significantly between 2010 and 2013. The reasons for this cannot be gauged from this analysis but it does raise concerns about the future of the epidemic if it is not better understood and dealt with.

Relationship of condom use and HIV prevention programmes has also been assessed by using AIDS Epidemic Model (AEM) [52] in many countries. The results of AEM among MSM in China showed a positive association between condom use and increased coverage of HIV intervention programme and concluded that the HIV prevention programme needed to be continued and strengthened to control the AIDS epidemic in MSM [53]. Findings of AEM in Peru, Ukraine, Kenya, and Thailand showed the positive impact of increased HIV prevention programme in reducing new HIV infections among MSM [54]. A modelling exercise conducted among MSM of Dhaka city using AEM showed that new HIV infections would be increasing in 2020 through various risk behaviours particularly male-to-male sex due to condom-less anal sex [55]. The findings also showed that 54% of the total new HIV infections will occur among MSM in 2020 compared to 12% in 2000 and hence recommended to increase coverage of HIV prevention for MSM in Dhaka city.

Several factors affect condom use and a major one is having comprehensive knowledge about HIV [56] that was reported to be low and did not improve in MSM over the three-year intervention period. The BCC leaflet that the peer-educators use to improve knowledge of HIV among MSM contains all correct information regarding HIV prevention and transmission. This analysis has revealed some important findings that need the attention of the programme implementers. At both time-points, a substantial percentage of MSM reported HIV can be prevented by using condoms correctly and consistently while on the other hand avoiding multiple sex partners as a mode of HIV prevention was reported to be low. Having correct knowledge that HIV is not transmitted by mosquito bites and not by sharing food with HIV infected persons was also reported to be low. These findings signal that when PEs conduct sessions to change risk behaviours using BCC with MSM, one-to-one or in a group, they stress only on correct and consistent use of condoms as a mode of HIV prevention and may ignore or do not stress enough on other ways of HIV prevention and transmission. However, misconception regarding HIV transmission that requires testing to know the status of HIV has increased at the midline compared to the baseline that highlights the benefit of HTS. In this analysis, since the prevalence of active-syphilis was low, self-reported symptoms of STIs was used as a proxy variable for prevalence of STIs. The association of self-reported STIs was found significant only with condom use in the last sex act. Those who used condom in the last sex act was less likely to report symptoms of STIs. Similar finding was observed in a study among MSM in Africa [57].

An outcome of knowledge is risk perception. In this analysis, more MSM at the midline perceived themselves to not be at risk, not because of lack of knowledge but because they used their knowledge on condoms and were therefore more likely to use condoms. It is essential to get everyone participating in the HIV prevention programme to be fully knowledgeable regarding routes of HIV transmission and means of prevention. In order to achieve this, assessment of the knowledge transfer techniques using BCC materials by the PEs need to be evaluated and improved.

This analysis is limited only to the outcome variables, condom use in the last sex act and consistently. The prevalence of HIV (<1%) and active syphilis (<2%) was very low among MSM in Dhaka city both at the baseline and midline [21, 22] and therefore, were not considered as outcome measures. Time location sampling encompassed to those MSM who were visible at the public cruising spots, therefore, results cannot be generalised to those who were hidden [58]; behavioural data may also be affected by social desirability and recall bias [59]. Furthermore, uunfortunately, we do not have data on what percentage of MSM participated in both baseline and in midline. Hence, pre-post design statistical analysis could not be performed.

## Conclusions

In summary, the results of the current analysis provide an evidence of positive changes in HIV risk behaviours among MSM in Dhaka city. However, the time gap between baseline and midline is only for three years therefore, a proper longitudinal study using similar methodology could better substantiate evidence to measure such sustainability. In sum to endure positive changes in HIV risk behaviours and end HIV in Bangladesh by 2030 [23], HIV prevention for MSM needs to be continued, strengthened and scaled up.

## Supporting information

S1 File (ZIP)
