# Supplementary material for: Changes in condom use among males who have sex with males (MSM): Measuring the effect of HIV prevention programme in Dhaka city
Source: PLoS One. 2020 Jul 24;15(7):e0236557. doi: 10.1371/journal.pone.0236557 (PMC7380615; doi:10.1371/journal.pone.0236557)
Supplement: S1 File — (ZIP) [file pone.0236557.s001.zip › MSM_ BSS Baseline English Questionnaire_2010.pdf]

Questionnaire identification number

MSM BEHAVIOURAL SURVEY FORM 2010 (PSA)

I (name) am working in a research project in collaboration with the Ministry of Health and Family Welfare GoB. We are trying to find out how to help people avoid a sickness called AIDS. We need to ask you some very personal questions. Nothing you tell me will be used for anything but the purposes of this survey. Your name or address will never be written down. You can refuse without any problem and you can stop the interview at any time after we start. What you tell me will be kept strictly confidential. Because we sincerely want to help all the people of Bangladesh avoid AIDS, if you agree to give the interview, it is really important that you are willing to be very truthful. Is it all right to begin?

Yes **No**

Name of the location: -

Cluster code:

Thana code:

Interviewer code:

Date Interview: ---------------------------------------

Interview starting time: -----------------------------

Interview ending time: ------------------------------

Did the interviewee abandon the interview:

Yes

No

Checked by the supervisor: Signature - - - - - - - - - - - - - - - - - - **Date: ------------------------**

Section 1: Introduction

No.	Questions and filters	Coding categories	Skip to	Comments
101.	How old are you?<br>(In completed years)	Years____		
102.	How many years of education have you completed up to now?	# Years Completed _____<br>Less than one year 00<br>No response 98<br>Never been to school 99		
103.	How long have you lived in this city (Dhaka)?	Number of Years ______<br>Record 00 if less than 1 year<br>All my life 96<br>Don't remember / know 97<br>No response 98		
104.	What is your total income from last month?	Tk. _______.<br>Don't know 97<br>No response 98		
105.	How did you earn that money<br><br>**Multiple answers possible**<br>**Provide ranking**	Ranking<br>Business 1 --------<br>Service 2 --------<br>Driving 3 --------<br>Tution/Teaching 4 --------<br>Family 5 --------<br>Others .... 6 -- --------		
106.	Have you sold blood for money in the past 12 months?	Yes….1<br>No….2<br>Don't Know…97<br>No Response…98		
107.	Some people take drugs for fun or to get high. Have you taken any drugs other than alcohol in the last 12 months?	Yes 1<br>No 2<br>Don't know 97<br>No response 98	**→ 109**	
108.	If yes, mention name of drugs?<br><br>**Do not read out**<br>**Multiple answere possible**<br>**If yes, circle 1**<br>**If no, circle 2**	Cannabis 1 2<br>Phensidyle 1 2<br>Tablet 1 2<br>Heroin 1 2<br>Injection 1 2<br>Yaba 1 2<br>Others -------------------- 1 2		
109.	Some people have tried injecting drugs for fun or to get high. Have you injected drugs in the last 12 months?	Yes 1<br>No 2<br>Don't Know 97<br>No Response 98	**→ 201**	
110.	Some people have tried injecting drugs for fun or to get high. Have you injected drugs in the last 2 months?	Yes….1<br>No….2<br>Don't Know…97<br>No Response…98		
111.	Last time you injected, did you use a needle or syringe after someone else had used it or did you pass your needle or syringe on to someone else in the last 12 past?	Yes 1<br>No 2<br>Don't Know/ Don't Remember 97<br>No Response 98		

Section 2: Marriage and Partnership and Sexual History

No.	Questions and filters	Coding categories	Skip to	Comments
201.	What is your current marital status?<br><br>READ OUT<br>**(Only one response)**	Married 1<br>Unmarried 2<br>Divorced 3<br>Widow 4<br>Separated 5<br>No Response 98	(2–98) → Q203	
202.	Are you currently living with spouse?	Yes 1<br>No 2<br>No Response 98		
203.	Are you currently living with a regular sex partner (except wife?	Yes .1<br>No. 2<br>No Response 98	**→205**	
204.	Is your sexual partner who you live with male, female or hijra?	Male 1 2<br>Female 1 2<br>Hijra 1 2<br>No response 98		
205.	At what age did you first have sexual intercourse?<br>(Anal and or vaginal sex)	Age In Years ____<br>Never had vaginal or anal sex 96<br>Don't Remember 97<br>No Response 98	**→331**	
206	Was your first sexual partner male, female or *hijra*?	Male 1<br>Female 2<br>Hijra 3<br>Don't remember 97<br>No response 98		
207A	Have you had anal sex with a male/*hijra* where taking money/by force gift in the last one-year?	Yes 1<br>No 2<br>No response 98	**→207c**	
207B	When did you last anal sex with male/*hijra* sex partners where in exchange of money/by force gift was involved?	------ months<br>0 (within 1 month)<br>Don't remember 97<br>No response 98		
207C	When did you last anal sex with male (not in *hijra*) sex partners in exchange of money/without money?	------ months<br>0 (within 1 month)<br>Don't remember 97<br>No response 98		
208	When did you last anal sex with *hijra* sex partners in exchange of money/without money?	------ months<br>0 (within 1 month)<br>No sex with *hijra* 96<br>Don't remember 97<br>No response 98		
209	When did you last vagainal/anal sex with female (not in *hijra*) sex partners in exchange of money/without money?	------ months<br>0 (within 1 month)<br>No sex with female 96<br>Don't remember 97<br>No response 98		
210.	Did you ever use condom during any **receptive/penetrative** sex act? **(Put Tick Mark)**	Yes…....1<br>No……2<br>No responses..…98		
211.	Did you use condom during your last receptive sex act in the **past 12 months**?	Yes….1<br>No….2<br>Never have receptive sex ….3<br>Never use condom….4<br>No response…98		
212.	Did you use condom during your last penetrative sex act in the **past 12 months**?	Yes….1<br>No….2<br>Never have penetrative sex ….3<br>Never use condom….4		

No.	Questions and filters	Coding categories	Skip to	Comments
		No response…98		
213.	Did you use condom in anal sex during your last receptive/penetrative sex act?	Yes….1<br>No….2<br>No response…98		

Section 3: Sexual Behaviour with Male Partners

Let's talk about non-paying male/Hijra partner

No.	Questions and filters	Coding categories	Skip to	Comments
300.	In the past **one-month**, have you had sex **(anal or oral)** with a man/hijra where no payment was involved?	Yes…....1<br>No……2	→306	
301.	In the past **one month**, how many different male/ hijra partners have you had sex with where no payment **(anal or oral)** was involved?	Number______<br>Don't know…97<br>No response.98		
302.	In the past **one month**, how many times have you had **anal sex** with your non-paying male/hijra sex partners?	Zero 0<br>Number …………..<br>Don't know….…97<br>No response…….98	→305	
303.	The last time **(in the past one month)** you had **anal sex** with a non-paying male/hijra partner, did you use a condom?	Yes......1<br>No…..2<br>Don't know…97<br>No response....98		
304.	Of all times you had **anal sex** with a non-paying male/hijra partner in the last month, how frequently did you use a condom?<br>**(Read out options 1-3)**	Always 1<br>Sometimes 2<br>Never 3<br>No response 98		
305.	In the **past one month**, how many different non-paying male partners did you have **oral sex** with?	Zero 0<br>Number …………..<br>Don't know….…97<br>No response…….98		

Now let's talk about non-paying female partner (not Hijras)

No.	Questions and filters	Coding categories	Skip to	Comments
306.	In the **past one-month**, have you had sex **(vaginal/ anal/oral)** with a woman where no payment was involved?	Yes…..1<br>No……2	→310	
307A	In the **past one month**, how many different female partners have you had sex **(vaginal/ anal/oral)** with where no payment was involved?	Number______<br>Don't know…97<br>No response.98		
307B	In the past **one month**, how many times have you had **vaginal or anal sex** with your non-paying female partners?	Number______<br>Don't know…97<br>No response.98		
308.	The last time you had **vaginal or anal sex** with a non-paying female partner, did you use a condom?	Yes......1<br>No…..2<br>Don't know…97<br>No response....98		
309.	Of all times you had **vaginal or anal sex** with a non-paying female partner in the last month, how frequently did you use a condom?<br>**(Read out options 1-3)**	Always 1<br>Sometimes 2<br>Never 3<br>No response 98		

Now let's talk about buying sex from women (not hijras)

No.	Questions and filters	Coding categories	Skip to	Comments
310.	In the **past one month**, have you paid any woman (not hijra) to have **(vaginal/anal/oral)** sex with you?	Yes…..1<br>No……2	→315	
311.	In the past one month, how many different women have you paid to have	Number______<br>Don't know…97		

No.	Questions and filters	Coding categories	Skip to	Comments
	**(vaginal/anal/oral**) sex with you?	No response.98		
312.	In the **past one month**, how many times did you pay to have **vaginal or anal** sex?	Zero 0<br>Number …………..<br>Don't know….....97<br>No response…….98	→**315**	
313.	The last time you paid a woman for **vaginal or anal sex**, did you use a condom?	Yes 1<br>No…..2<br>Don't know…97<br>No response....98		
314.	Of all times you paid a woman for **vaginal/anal sex** in the **past one month**, how frequently did you use a condom?<br>**(Read out options 1-3)**	Always 1<br>Sometimes 2<br>Never 3<br>No response 98		

Let's talk about buying sex from men (not hijra)

No.	Questions and filters	Coding categories	Skip to	Comments
315.	In the **past one month**, have you paid another man (not hijra) to have **(anal or oral)** sex with you?	Yes…..1<br>No……2	→**321**	
316.	In the **past one month**, how many different men have you paid to have **(anal or oral)** sex with you?	Number______<br>Don't know….97<br>No response…..98		
317.	In the past one month, how many times did you pay to have **anal sex** with men?	Zero 0<br>Number …………..<br>Don't know….....97<br>No response…….98	→**320**	
318.	The last time you paid for **anal sex** with a man, did you use a condom?	Yes…..1<br>No……2<br>Don't know….....97<br>No response…….98		
319.	Of all times you paid for **anal sex** with a man in the last month, how frequently did you use a condom?<br>(**Read out options 1-3**)	Always 1<br>Sometimes 2<br>Never 3<br>No response 98		
320.	In the **past one month**, how many different men have you paid to have **oral sex** with you?	Zero 0<br>Number …………..<br>Don't know….....97<br>No response…….98		

Let's talk about buying sex from hijras

No.	Questions and filters	Coding categories	Skip to	Comments
321.	In the past one month, have you paid a hijra to have **(anal or oral)** sex with you?	Yes…..1<br>No……2	→**327**	
322.	In the **past one-month**, how many different hijras have you paid to have **(anal or oral)** sex with you?	Number …………..<br>Don't know….....97<br>No response…….98		
323.	In the **past one-month**, how many different hijras did you pay to have **anal sex** with?	Zero 0<br>Number …………..<br>Don't know….....97<br>No response…….98	→**326**	
324.	The last time you paid a hijra for **anal sex**, did you use a condom?	Yes…..1<br>No……2<br>Don't know….....97<br>No response…….98		
325.	Of all times you paid a hijra for anal **sex in the past one month**, how frequently did you use a condom?<br>**(Read out options 1-3)**	Always 1<br>Sometimes 2<br>Never 3<br>No response 98		
326.	In the **past one month**, how many different	Zero 0		

No.	Questions and filters	Coding categories	Skip to	Comments
	hijras have you paid to have **oral sex** with you?	Number …………..<br>Don't know…....97<br>No response…….98		

Let's talk about Group Sex

(Vaginal/oral/anal sex with more than 1 partner at the same time)

No.	Questions and filters	Coding categories	Skip to	Comments
327.	Have you had sex in a group in the past one month?	Yes…..1<br>No……2<br>Don't know…....97<br>No response…….98	**→331**	
328.	Last time you had sex in a group, how many partners (including yourself) were there?	Number …………..<br>Don't know…....97<br>No response…….98		
329.	Last time you had sex in a group, how many of the partners besides you used condom?	Number …………..<br>Don't know…....97<br>No response…….98		
330.	Last time you had sex in a group, did you yourself use a condom?	Yes…..1<br>No……2<br>Don't know…....97<br>No response…….98		

Section 3B: Let's talk about Mobility (inside country or outside country) & commercial or non-commercial Sex

No.	Questions and filters	Coding categories	Skip to	Comments
331.	Have you visited outside this city in the last year (inside country)?	Yes…..1<br>No……2<br>No response…….98	**→336**	
332.	Have you bought sex in another city in the **past 12 months**?	Yes…..1<br>No……2<br>No response…….98	**→334**	
333.	Did you use condom in the last sex act while buying sex in another city in the last year?	Yes…..1<br>No……2<br>No response…….98		
334.	Have you had sex in another city in the **past 12 months** where no payment was made?	Yes…..1<br>No……2<br>No response…….98	**→336**	
335.	Did you use condom in the last non-commercial sex act in another city in the last year?	Yes…..1<br>No……2<br>No response…….98		
336.	Have you been to any other country outside Bangladesh during the past year?	Yes…..1<br>No……2<br>No response…….98	**→401**	
337.	Have you bought sex in another country in the **past year**?	Yes…..1<br>No……2<br>No response…….98	**→339**	
338.	Did you use condom during your last bought sex act outside the country in the **past 12 months**?	Yes…..1<br>No……2<br>No response…….98		
339.	Have you had sex in another country in the **past 12 months** where no payment was made?	Yes…..1<br>No……2<br>No response…….98	**→401**	
340.	Did you use condom during your last non-commercial sex act outside the country in the **past 12 months**?	Yes…..1<br>No……2<br>No response…….98		

Section 4: Let's talk about Male Condoms and Lubricant

No.	Questions and filters	Coding categories	Skip to	Comments
401.	**SHOW CONDOM and ask**<br>"Can you tell me what this is"?	Can identify as condom 1<br>Cannot identify as condom 2<br>No response 98	 →406	
402.	Do you have a condom with you now? Please show me	Can show a condom…1<br>Cannot show a condom 2<br>No response 98		
403.	Which places or persons have you obtained condoms from in the last one month?<br><br>Multiple answers possible<br><br>**Do not read out**<br>**(Circle 1 if mentioned)**<br>**(Circle 2 if not mentioned)**	Don't know where condom available 1 2<br>Shop….. 1 2<br>Pharmacy….. 1 2<br>DIC------1 2<br>Health facility (Excluding DIC)…… 1 2<br>Bar/Guest House/Hotel….. 1 2<br>Friends…… 1 2<br>NGO worker….. 1 2<br>Did not buy condom in last month----1 2<br>Never used condom 1 2<br>Did not get condom 1 2<br>Other-------------------------------- 1 2<br>No response…..98	→ if 1, then go to q406	
404.	Can you obtain a condom every time you need one?	Yes…..1<br>No……2<br>Don't need one…3<br>Never used condom 4<br>Don't know……97<br>No response…….98	**→406**<br>**→405**<br><br>**→406** (3, 4, 97, 98)	
405.	Why can't you get a condom every time you need one?<br><br>**Multiple answers possible**<br>**Do not read out**<br>**(Circle 1 if mentioned)**<br>**(Circle 2 if not mentioned)**	Cost too much…..1 2<br>Shop/pharmacy too far away……1 2<br>Shops pharmacy closed……1 2<br>Shy to buy condom…….1 2<br>Don't know where to obtain…….1 2<br>Don't want to carry them ……1 2<br>No need------1 2<br>Never use in life-----1 2<br>Other__________________1 2<br>Don't know……….97<br>No response……….98		
406.	Have you ever used lubricant when having anal sex?<br>**I mean something to make your own or your partner's penis slippery so it is easier to insert**	Yes….. 1<br>No…… 2<br>Don't know…… 97<br>No response……. 98	 **→409**	
407.	What lubricant did you use during last anal sex?<br><br>Multiple responses possible<br>Do Not Read Out	Saliva……1 2<br>Oil……..1 2<br>Water-based condom lubricant…….1 2<br>Antiseptic cream…….1 2<br>Normal lotion….....1 2<br>Vasline/Jelli/Beuti Cream……1 2<br>Other_____________1 2<br>Don't know..................97<br>Not response............98		
408.	Were you using a condom that time?	Yes………1<br>No...............2<br>Don't know… 97<br>No response… 98		
409.	Some people use a lubricant product made especially for use with condoms. Have you heard of such a product?	Yes…..1<br>No……2<br>Don't know……97<br>No response…….98	 **→414**<br>**→414**<br>**→414**	
410.	Can you tell me the brand name of such a	Yes…..1		

No.	Questions and filters	Coding categories	Skip to	Comments
	product?	Record Name ___________________<br>No……2<br>Don't know……97<br>No response…….98		
411.	In the past one month, how frequently have you used a special lubricant for condoms together with a condom during anal sex?	Always………1<br>Sometimes………2<br>Never 3<br>Don't know……97<br>No response……..98<br>No sex in last month……..99	→413<br>→412<br>→412<br>→414<br>→414<br>→510	
412.	Why do you sometimes not use special condom lubricant, or never use it?<br><br>**Multiple answers possible**<br>**Do not read out**<br>**(Circle 1 if mentioned)**<br>**(Circle 2 if not mentioned)**	Costs too much…….1 2<br>Shy to buy lubricant…….1 2<br>Don't know where to obtain……1 2<br>I do not need to use…….1 2<br>I use other cream…….1 2<br>Supply short……1 2<br>Not easy to carry…….1 2<br>Other___________________1 2<br>I don't remember…….97<br>No response…….98	→414	
413.	For you, what are the purposes of using special condom lubricant with condoms during sex?<br><br>**Multiple answers possible**<br>**Do not read out**<br>**(Circle 1 if mentioned)**<br>**(Circle 2 if not mentioned)**	Decrease pain/inflammation..1 2<br>Increase feeling……….1 2<br>Decrease risk of condom breakage..1 2<br>Prevent HIV/AIDS infection…..1 2<br>Other ____________________1 2<br>Don't know……97<br>No response……..98		
414.	In the past one month, breakage condom when use it?	Yes………1<br>No..............2<br>Don't know… 97<br>No condom use in last month…3<br>Never use condom in life…4<br>No response… 98<br>No sex in last month…99	 <br><br>→501<br>→501<br>→501<br>→501	
415.	Sources of condom in the past one month?<br><br>**Multiple answers possible**<br>**Do not read out**<br>**(Circle 1 if mentioned)**<br>**(Circle 2 if not mentioned)**	Shop….. 1 2<br>Pharmacy….. 1 2<br>DIC……1 2<br>Bar/Guest House/Hotel….. 1 2<br>Friends…… 1 2<br>Broker (Dalal)…. 1 2<br>NGO worker….. 1 2<br>Bought condom in last month…..1 2<br>Sex partner……1 2<br>Other-------------------------------- 1 2<br>Don't know……97<br>No response…....98		

Section 5: Let's talk about STDs

No.	Questions and filters	Coding categories	Skip to	Comments
501.	Could you describe any symptoms in men of diseases that can be transmitted by having sex?<br><br>DO NOT READ OUT<br>Circle 1 when mentioned<br>Circle 2 when not mentioned<br>(Multiple responses possible)	Penis discharge 1 2<br>Burning pain on urination 1 2<br>Genital ulcers/sores 1 2<br>Swellings in groin area 1 2<br>Anal discharge 1 2<br>Anal ulcer/sores 1 2<br>Other: …………….…………. 1 2<br>Don't know 97<br>No response 98		
502.	Have you had a urethral discharge during the past 12 months? (Something liquid and sticky	Yes 1<br>No 2		

No.	Questions and filters	Coding categories	Skip to	Comments
	but not semen)	Don't know 97<br>No Response 98		
503.	Have you had anal discharge during the last 12 months? (Something liquid and sticky)	Yes 1<br>No 2<br>Don't know 97<br>No Response 98		
504.	Have you had a genital ulcer / sore during the past 12 months?	Yes 1<br>No 2<br>Don't know 97<br>No response 98		
505.	**Had genital ulcer / discharge / sore (penis and or anal) during the past 12 months?**<br>If yes in any of Q502, 503, 504- then circle 1 of 505 otherwise circle 2.	Yes 1<br>No 2	 **→509**	
506A.	What was the first thing you did when you had those symptoms in last time?<br><br>DO NOT READ OUT<br>Only one response	Treatment from hospital……1<br>Treatment from drug seller……2<br>Treatment from private doctor……3<br>Treatment from private clinic……4<br>Treatment from NGO clinic ……5<br>Treatment from traditional healer……6<br>Advice/treatment from friend……7<br>Self treatment…… 8<br>Nothing .... 9<br>Other__________________10<br>Don't know 97<br>No Response 98	**→Ask 506B**<br><br><br>**→509**	
506B.	Name of NGO clinic?			
507.	Last time you had one of those symptoms that you just told me about, how many days did you wait between discovering symptoms and going for treatment<br>**(If the same day, Var. name 1)**	Number of days____________<br>Don't know 97<br>No response 98		
508	Last time you had those symptoms, how much did the treatment cost you, including the medicine and the fees for the service?	Tk_________<br>Don't know 97<br>No response 98		
509.	Do you yourself do anything to avoid getting diseases which are transmitted by sex?<br><br>Multiple answers possible<br>**(DO NOT READ OUT)**<br>**(Circle 1 if mentioned)**<br>**(Circle 2 if not mentioned)**	Nothing….1 2<br>Wash genitals with dettol or urine after sexual intercourse…. 1 2<br>Always use condoms 1 2<br>Sometimes use condom 1 2<br>Always trusted sex partners … 1 2<br>Sex partners test before sex/sex with neat clean partners…. 1 2<br>Other__________________1 2<br>Take medicine……1 2<br>Don't know 97<br>No response 98	**→ask 510**	
510.	What medicine do you take?	Name________________<br>Don't know 97<br>No response 98		
511	During the last month have you visited an NGO STI clinic that is working with men who have sex with men in this city?	Yes 1<br>No 2<br>Don't know 97<br>No response 98	 **→601**	
512	If yes, which clinic was it?<br>(Multiple answer possible)	Name(s) of the clinic<br>……………………………<br><br>……………………………		

Section 6: Let's talk about AIDS knowledge, risk and avoidance

No.	Questions and filters	Coding categories	Skip to	Comments

No.	Questions and filters	Coding categories	Skip to	Comments
601.	Have you ever heard of HIV or the disease called AIDS?	Yes 1<br>No 2<br>No response 98	→ 701	
602.	Can people reduce their risk of HIV by using a condom correctly every time they have sex?	Yes 1<br>No 2<br>Don't know ……97<br>No response 98		
603.	Can people reduce their risk of HIV by avoiding anal sex?	Yes 1<br>No 2<br>Don't know ……97<br>No response……98		
604.	Can people reduce their risk of HIV by using a condom correctly every time they have vaginal/anal sex?	Yes 1<br>No 2<br>Don't know ……97<br>No response……98		
605.	Can people reduce their risk of HIV by avoiding multiple sexual partners?	Yes 1<br>No 2<br>Don't know ……97<br>No response……98		
606.	Can a person get the HIV from mosquito bites?	Yes 1<br>No 2<br>Don't know ……97<br>No response……98		
607.	Can a person get HIV by sharing a meal with someone who is infected?	Yes 1<br>No 2<br>Don't know ……97<br>No response……98		
608.	Can a person get HIV by taking injections with a needle that has already been used by someone else?	Yes 1<br>No 2<br>Don't know ……97<br>No response……98		
609.	Do you think you can tell by looking at someone whether they are infected with HIV?	Yes 1<br>No 2<br>Don't know ……97<br>No response……98		
610.	What do you yourself do to avoid getting HIV?<br><br>**(Multiple responses possible)**<br><br>**(Do not read out)**<br>**Circle 1 if mentioned**<br>**Circle 2 if not mentioned**	Nothing….1 2<br>Wash genitals with dettol or urine after sexual intercourse ………1 2<br>Always use condoms ….. 1 2<br>Sometimes use condom.... 1 2<br>Take medicine…… 1 2<br>Always sex with trusted partner/sex with clean partners …… 1 2<br>Other___________________1 2<br>Don't know 97<br>No response 98		

Confidential test for HIV

(Confidential means that no one will know the result if you don't want them to know)

No.	Questions and filters	Coding categories	Skip to	Comments
611.	Do you know anywhere you could go if you wanted to get a confidential test to find out if you are infected with HIV?	Yes 1<br>No 2<br>Don't know ……97<br>No response 98	→ 701	
612.	I don't want to know the result, but have you ever had an HIV test?	Yes 1<br>No 2<br>Don't know …… 97<br>No response……. 98	→ 701	
613.	If yes, wher done HIV test?			

No.	Questions and filters	Coding categories	Skip to	Comments
614.	Did you yourself request the test or did someone offer you to test for AIDS, or were you required to have the test?	Self 1<br>Some one offered 2<br>Required 3<br>No response 98	→ ask 617<br>→ don't ask 616<br>→ don't ask 615	
615.	Who request to your test? **(only 1 response)**			
616.	Why need the test? **(only 1 response)**			
617.	Please do not tell me the result, but did you get the result of your test?	Yes 1<br>No 2<br>Don't know ……97<br>No response 98		
618.	When did you have the most recent HIV test?	Within the past year ……1<br>More than a year ago ………2<br>Don't know 97<br>No response 98		

Section 7: Let's Talk about Violence

No.	Questions and filters	Coding categories	Skip to	Comments
701.	In the past 12 months, were you ever beaten?	Yes 1<br>No 2<br>Don't remember 97<br>No response 98	→ 703<br>→ 703	
702.	If yes, Who beaten?<br><br>**DO NOT READ OUT**<br>**(Multiple answer possible)**<br>**Circle 1 if mentioned**<br>**Circle 2 if not mentioned**	Mean in uniform….. 1 2<br>Mastans…… 1 2<br>New sex partner….. 1 2<br>Regular sex partner…. 1 2<br>Loacal people …. 1 2<br>Family/Realtives …. 1 2<br>Others_____________ 1 2<br>Don't know… 97<br>No responses…. 98		
703.	In the past 12 months, were you physically forced to have sex with someone even though you did not want to?	Yes 1<br>No 2<br>Don't remember 97<br>No response 98	→ 705<br>→ 705	
704.	Who was the person (or people) who physically forced you to have sex against your will or beaten you?<br><br>**Multiple answers possible**<br>**(DO NOT READ OUT)**<br>**(Circle 1 if mentioned)**<br>**(Circle 2 if not mentioned)**	Mean in uniform….. 1 2<br>Mastans…… 1 2<br>New sex partner….. 1 2<br>Regular sex partner…. 1 2<br>Loacal people …. 1 2<br>Realtives …. 1 2<br>Others_____________ 1 2<br>Don't know… 97<br>No responses…. 98		
705.	During the past 12 months, have you been arrested?	Yes 1<br>No 2<br>Don't know ……97<br>No response 98	→ 801	
706.	If yes, what is reason for arrest?	1. ………………………………………<br>2. ………………………………………<br>3. ………………………………………		

Section 8: Risk Perceptions

No.	Questions and filters	Coding categories	Skip to	Comments
801.	I want to ask if you yourself think you are risk for HIV. Do you think that you are at high risk, some risk or little or no risk?	High risk……..1<br>Some risk……..2<br>Little or no risk……..3<br>Don't know ……97<br>No response……98	→ 802<br>→ 802<br>→ 803<br>→ 901<br>→ 901	

No.	Questions and filters	Coding categories	Skip to	Comments
802.	Why do you think you are at risk for HIV?<br><br>Multiple answers possible<br>**(DO NOT READ OUT)**<br>**(Circle 1 if mentioned)**<br>**(Circle 2 if not mentioned)**	High risk job ……. 1 2<br>Frequent anal sex …….. 1 2<br>Don't use condoms…….. 1 2<br>Irregular condom use..... 1 2<br>Injections sharing……….. 1 2<br>Other-------------------------------- 1 2<br>Don't know ……97<br>No response……98	→ 901	
803.	Why do you think you are at little or no risk of HIV?<br><br>Multiple answers possible<br>**(DO NOT READ OUT)**<br>**(Circle 1 if mentioned)**<br>**(Circle 2 if not mentioned)**	Always use condoms ……..1 2<br>Partners are clean ……….1 2<br>Partners are healthy.......1 2<br>Never share injections 1 2<br>Sometimes share injections 1 2<br>Irregular use of condom 1 2<br>Always have sex with single trusted . partner 1 2<br>Other___________________1 2<br>Don't know ……97<br>No response 98		

Section 9: Exposure to interventions

No.	Questions and filters	Coding categories	Skip to	Comments
901.	Have you participated in any NGO/Self help group/CBO - run AIDS prevention program in the **past 12 months**?	Yes 1<br>No 2<br>No NGO/Self help group/CBO in this area 96<br>Don't know ……97<br>No response 98	No, 96, 97 → **907**	
902.	When you have last participated in any NGO/Self help group/CBO - run AIDS prevention program?	…………….. months<br>0 (If withn in a month) 96<br>Don't know/remember 97<br>No response 98		
903.	How long have you been involved in this activity/program?	…………….. months<br>0 (If withn in a month) 96<br>Don't know/remember 97<br>No response 98		
904.	If participated, how many times in the **past month**?	Zero 0<br>Number …..<br>Don't know 97<br>No response 98		
905.	If yes, which type of activities have you participated in (ever)?<br><br>(Do not read out)<br>(Multiple answers possible)<br>**Circle 1 if mentioned**<br>**Circle 2 if not mentioned**	Needle exchange program 1 2<br>Education 1 2<br>Received condoms 1 2<br>Treatment STD 1 2<br>Attending IHC 1 2<br>Attending DIC 1 2<br>Attending VCT 1 2<br>Other ....1 2<br>Don't Know 97<br>No Response 98		
906.	How did you benefit from the sessions?<br><br>**(Multiple answers possible)**<br><br>**DO NOT READ OUT**<br>**Circle 1 if mentioned**<br>**Circle 2 if not mentioned**	Helped you change your behavior 1 2<br>Gave useful information but did not affect behaviour 1 2<br>Learnt about HIV/AIDS/STD/safe sex and correct use of condom 1 2<br>Information was not easily understandable 1 2<br>Was not relevant to our needs……. 1 2<br>Other ---------------------------------- 1 2		

Section 10: Meeting place and Miscellaneous

No.	Questions and filters	Coding categories	Skip to	Comments
907.	Why were do you usually meet your friends?<br><br>**(Multiple answers possible)**<br><br>**DO NOT READ OUT**<br>**Circle 1 if mentioned**<br>**Circle 2 if not mentioned**	Cruising spot 1 2<br>Residence 1 2<br>Club 1 2<br>Party 1 2<br>Tea stall 1 2<br>On the street 1 2<br>Other....................................... 1 2<br>Don't know/remember 97<br>No response 98		
908.	How do you contact your male sex partner for having sex?<br><br>**(Multiple answers possible)**<br><br>**DO NOT READ OUT**<br>**Circle 1 if mentioned**<br>**Circle 2 if not mentioned**	Cruising spot 1 2<br>Phone 1 2<br>Internet 1 2<br>Through friends 1 2<br>Pimp 1 2<br>In clubs 1 2<br>In parties 1 2<br>Other... 1 2<br>Don't know/remember 97<br>No response 98		
909	With regards to your sexual behavior, how would you identify yourself?<br><br>**DO NOT READ OUT**<br><br>(Only one response)	Man/manly/general people 1<br>Parikh 2<br>Film hero 3<br>Panthi 4<br><br>Film heroin 5<br>Gay 6<br>Koti 7<br>Do-parata 8<br>Girls/woman 9<br>Other: ……………………… 10<br>Don't Know 97<br>No response 98	(1–4) → **stop the interview with thanks**<br><br>(5–9) → **910**	
910	Have you taken any medicine for feminine?	Yes 1<br>No 2<br>Don't Know 97<br>No response 98	→ **Stop interview with thanks**	
911	If yes, have you taken in last 3 months?	Yes 1<br>No 2<br>Don't Know 97<br>No response 98	→ **Stop interview with thanks**	
910	Mention the name of medicine which taken in the last 3 months?<br><br>**(Multiple answers possible)**<br><br>**DO NOT READ OUT**<br>**Circle 1 if mentioned**<br>**Circle 2 if not mentioned**	Shukhi 1 2<br>Cilest-21 1 2<br>Desolon 1 2<br>Lynes 1 2<br>Marvelon 1 2<br>Ovostat 1 2<br>Femecon 1 2<br>Nordat-28 1 2<br>Emcon 1 2<br>Postinor-2 1 2<br>Minicon 1 2<br>Ovacon 1 2<br>Others ……… 1 2<br>Don't Know 97<br>No response 98		
913.	Mention the cause of taken medicine in the last 3 months?<br><br>**(Multiple answers possible)**<br>**DO NOT READ OUT**<br>**Circle 1 if mentioned**<br>**Circle 2 if not mentioned**	Increased breast size 1 2<br>Increased shape of thigh/hip 1 2<br>Increased smoothness of body 1 2<br>Dari ghop na gozanor jonno 1 2<br>Others ……… 1 2<br>Don't Know 97<br>No response 98		

No.	Questions and filters	Coding categories	Skip to	Comments
914.	How frequently taken that medicine in the last 3 months?<br>**(Multiple answers possible)**<br>**DO NOT READ OUT**<br>**Circle 1 if mentioned**<br>**Circle 2 if not mentioned**	Every day 1 2<br>More than one a week 1 2<br>Onece in a week 1 2<br>Once in a month 1 2<br>Don't Know/remember 97<br>No response 98		

Thank you very much for your kind cooperation and spending your valuable time with me.

= < =
