# Supplementary material for: Changes in condom use among males who have sex with males (MSM): Measuring the effect of HIV prevention programme in Dhaka city
Source: PLoS One. 2020 Jul 24;15(7):e0236557. doi: 10.1371/journal.pone.0236557 (PMC7380615; doi:10.1371/journal.pone.0236557)
Supplement: S1 File — (ZIP) [file pone.0236557.s001.zip › MSM_ BSS Midline Bangla Questionnaire_2010.pdf]

এম এস এমদের (MSM) আচরণগত সার্ভে ফর্ম ২০১০ (PSA)

আমি (নিজের নাম বলুন) Global Fund (GF) এর অর্থায়নে Rolling Continuation Channel (RCC), ICDDR,B (কলেরা হাসপাতাল, মহাখালী, ঢাকা) project এর পক্ষ থেকে এম এস এমদের সম্পর্কে বিভিন্ন তথ্যাবলী জানার জন্য একটি জরীপ প্রকল্পে কাজ করছি। এইডস নামক রোগ যাতে না হয় সে ব্যপারে মানুষকে কি ভাবে সাহায্য করা যায় আমরা তা জানার চেষ্টা করছি। এ ব্যপারে আপনাকে কিছু একান্ড় ব্যক্তিগত প্রশ্ন জিজ্ঞাসা করা প্রয়োজন। কারন আমরা আন্ড়রিক ভাবে চাই, আপনাদের মধ্যে কারো যাতে HIV বা AIDS না হয়। আপনি আমাকে যা বলবেন তা সম্পূর্ণভাবে গোপন রাখা হবে। সাক্ষাৎকার শুর্ব করার পর আপনি যে কোন সময়ে সাক্ষাৎকার প্রদান বন্ধ করে দিতে পারবেন। আপনি যদি সাক্ষাৎকার দিতে রাজী থাকেন, তা হলে সম্পূর্ণ সত্য কথা বলা অত্যন্ড় গুরুত্বপূর্ন। আমরা কি তাহলে সাক্ষাৎকার শুর্ব করতে পারি ?

হ্যাঁ না

জরীপ এলাকা :---------------------------------

ক্লাষ্টার নং ঃ ☐

থানা কোড ঃ ☐

সাক্ষাৎকার গ্রহনকারীর কোড ঃ ☐

সাক্ষাৎকার গ্রহণকরার তারিখ :--------------------------------------

সাক্ষাৎকার শুর্ব করার সময় :---

সাক্ষাৎকার শেষ করার সময় :---------------------------------------

---

সাক্ষাৎকার গ্রহন কি সম্পন্ন হয়েছে ?

হ্যাঁ ☐

না ☐

সুপারভাইজারের স্বাক্ষর ....

তারিখ ঃ ....../....../........

সেকশন ১ঃ পটভূমি

নং	প্রশ্ন	কোডের ধরন	নির্দেশ	মন্ড্র্ব্য
১০১	আপনার বয়স কত? (পূর্ণ বছরে লিখুন)	বৎসর---------------		
১০২	আপনি কত (শ্রেনী) পর্যন্ড় লেখাপড়া করেছেন?	সম্পন্নকৃত বৎসর----- ------<br>কখনও স্কুলে যায় নাই ৯৯<br>১ বৎসরের কম ০০<br>উত্তর না দেয়া ৯৮		
১০৩	এই শহরে (ঢাকা) আপনি কতদিন যাবৎ বসবাস করছেন?	বৎসর ______<br>১ বৎসরের কম ০০<br>সারাজীবন ৯৬<br>মনে নাই/জানি না ৯৭<br>উত্তর না দেয়া ৯৮		
১০৪	গত মাসে আপনার মোট আয় কত?	টাকা --------<br>জানি না ৯৭<br>উত্তর না দেয়া ৯৮		
১০৫	কিভাবে এই টাকা উপার্জন করেছেন?<br><br>**(একাধিক উত্তর সম্ভব)**<br>**(র‌্যাংকিং কর‍ুন)**	র‌্যাংকিং<br>ব্যবসা ১ ----<br>চাকুরী ২ ----<br>ড্রাইভার ৩ ----<br>টিউশনি/শিক্ষকতা ৪ ----<br>পরিবার ৫ ----<br>অন্যান্য ---------------------------- ৬ ----		
১০৬	গত ১২ মাসে কি আপনি টাকার জন্য রক্ত বিক্রি করেছেন?	হ্যাঁ ১<br>না ২<br>জানি না ৯৭<br>উত্তর না দেয়া ৯৮		
১০৭	কিছু লোক মজা করার জন্য বা নেশার জন্য মাদক দ্রব্য নেয়। গত ১২ মাসে আপনি কি **মদ ছাড়া** অন্য কোন মাদকদ্রব্য নিয়েছেন?	হ্যাঁ ১<br>না ২<br>জানি না ৯৭<br>উত্তর না দেয়া ৯৮	→ ১০৯	
১০৮	যদি হ্যাঁ হয়, তবে কি নিয়েছেন ?<br><br>**(পড়ে শোনাবেন না)**<br>**(একাধিক উত্তর সম্ভব)**<br>**(উলেণ্টখ করলে ১ -এ গোল কর‍ুন)**<br>**(উলেণ্টখ না করলে ২-এ গোল কর‍ুন)**	গাঁজা ১ ২<br>ফেন্সিডিল ১ ২<br>ট্যাবলেট ১ ২<br>হেরোইন ১ ২<br>ইঞ্জেকশন ১ ২<br>ইয়াবা ১ ২<br>অন্যান্য ----------------------------- ১ ২		
১০৯	কিছু লোক মজার জন্য বা নেশার জন্য ইনজেকশন নেয়। গত ১২ মাসে আপনি কি নেশার জন্য ইনজেকশন নিয়েছেন?	হ্যাঁ ১<br>না ২<br>জানি না ৯৭<br>উত্তর না দেয়া ৯৮	→ ২০১	
১১০	কিছু লোক মজা করার জন্য বা নেশার জন্য ইনজেকশন নেয়। গত ২ মাসে আপনি কি নেশার জন্য ইনজেকশন নিয়েছেন ?	হ্যাঁ ১<br>না ২<br>জানি না ৯৭<br>উত্তর না দেয়া ৯৮		
১১১	গত ১২ মাসে নেশার ইনজেকশন নেয়ার সময় আপনি কি আপনার ব্যবহৃত সূঁচ/সিরিঞ্জ অন্য কাউকে দিয়েছেন বা অন্যের ব্যবহৃত সূঁচ/সিরিঞ্জ আপনি নিয়েছেন?	হ্যাঁ ১<br>না ২<br>জানি না /মনে নাই ৯৭<br>উত্তর না দেয়া ৯৮		

সেকশন ২ ঃ বিবাহ, সঙ্গী এবং যৌন ইতিহাস

নং	প্রশ্ন	কোডের ধরন	নির্দেশ	মন্ড্ব্য
২০১	বর্তমানে আপনার বৈবাহিক অবস্থা কি?<br>**(একটি মাত্র উত্তর হবে)**	বিবাহিত ১<br>অবিবাহিত ২<br>ডিভোর্সড ৩<br>বিপত্নিক ৪<br>আলাদা বসবাস ৫<br>উত্তর না দেয়া ৯৮	(২–৯৮) → ২০৩	
২০২	বর্তমানে আপনি কি আপনার স্ত্রীর সাথে বসবাস করছেন?	হ্যাঁ ১<br>না ২<br>উত্তর না দেয়া ৯৮		
২০৩	বর্তমানে আপনার কি কোন নিয়মিত যৌনসঙ্গী আছে (স্ত্রী **ছাড়া**)?	হ্যাঁ ১<br>না ২<br>উত্তর না দেয়া ৯৮	→ ২০৫	
২০৪	যদি হ্যাঁ হয়, আপনার বর্তমান নিয়মিত যৌনসঙ্গী পুরুষ, মহিলা, না হিজড়া?<br>**(একাধিক উত্তর সম্ভব)**	পুরুষ ১ ২<br>মহিলা ১ ২<br>হিজড়া ১ ২<br>উত্তর না দেয়া ৯৮		
২০৫	কত বৎসর বয়সে আপনি প্রথম যৌনমিলন করেছেন? **(পায়ু/যোনীপথে)**	বৎসর ---<br>কখনো না ৯৬<br>মনে নাই ৯৭<br>উত্তর না দেয়া ৯৮	→ ৩৩১	
২০৬	আপনার প্রথম যৌনসঙ্গী পুরুষ, মহিলা না হিজড়া ছিল?<br>**(একটি মাত্র উত্তর হবে)**	পুরুষ ১<br>মহিলা ২<br>হিজড়া ৩<br>মনে নাই ৯৭<br>উত্তর না দেয়া ৯৮		
২০৭ক	আপনি কি কখনো টাকা নিয়ে অথবা বাধ্যতামূলক উপহার নিয়ে পুরুষ/হিজড়ার খদ্দেরের সাথে **পায়ুপথে** যৌনমিলন করেছেন?	হ্যাঁ ১<br>না ২	(২) → ২০৭গ	
২০৭খ	আপনি সর্বশেষ কবে টাকা নিয়ে অথবা বাধ্যতামূলক উপহার নিয়ে পুরুষ/হিজড়ার খদ্দেরের সাথে **পায়ুপথে** যৌনমিলন করেছেন?	. . . . মাস<br>০ (যদি ১ মা‡সর মধ্যে হয়)<br>মনে নাই/জানি না ৯৭<br>উত্তর না দেয়া ৯৮		
২০৭গ	আপনি সর্বশেষ কত মাস আগে **পুরুষের** (হিজড়া নয়) সাথে (টাকা দিয়ে/ টাকা ছাড়া) **পায়ুপথে** যৌনমিলন করেছেন?	. . . . মাস<br>০ (যদি ১ মা‡সর মধ্যে হয়)<br>মনে নাই/জানি না ৯৭<br>উত্তর না দেয়া ৯৮		
২০৮	আপনি সর্বশেষ কত মাস আগে **হিজড়ার** সাথে (টাকা দিয়ে/টাকা ছাড়া) **পায়ুপথে** যৌনমিলন করেছেন?	. . . . মাস<br>০ (যদি ১ মা‡সর মধ্যে হয়)<br>হিজড়ার সাথে যৌনমিলন করে নাই ৯৬<br>মনে নাই/জানি না ৯৭<br>উত্তর না দেয়া ৯৮		
২০৯	আপনি সর্বশেষ কত মাস আগে কোন মহিলার সাথে (হিজড়া নয়) (টাকা দিয়ে/টাকা ছাড়া) **যোনী অথবা পায়ুপথে** যৌনমিলন করেছেন?	. . . . মাস<br>০ (যদি ১ মা‡সর মধ্যে হয়)<br>মহিলার সাথে যৌনমিলন করে নাই ৯৬<br>মনে নাই/জানি না ৯৭<br>উত্তর না দেয়া ৯৮		
২১০	আপনি কি জীবনে কখনও প্রবেশম□লক (আপনার লিঙ্গ	হ্যাঁ ১		

নং	প্রশ্ন	কোডের ধরন		নির্দেশ	মন্তব্য
	যৌনসঙ্গীর পায়ুপথে প্রবেশ করিয়েছেন) অথবা গ্রহনম□লক (যৌনসঙ্গী তার লিঙ্গ আপনার পায়ুপথে প্রবেশ করিয়েছে) যৌনমিলনে কনডম ব্যবহার করেছেন?	না<br>উত্তর না দেয়া	২<br>৯৮		
২১১	শেষবার গ্রহনমূলক (যৌনসঙ্গী তার লিঙ্গ আপনার পায়ুপথে প্রবেশ করিয়েছে) যৌন কাজের সময় আপনি কি কনডম ব্যবহার করেছিলেন?	হ্যাঁ<br>না<br>কখনো গ্রহনমূলক যৌন কাজ করে নাই<br>কখনও কনডম ব্যবহার করে নাই হলে<br>উত্তর না দেয়া	১<br>২<br>৩<br>৪<br>৯৮		
২১২	শেষবার প্রবেশমূলক (আপনার লিঙ্গ যৌনসঙ্গীর পায়ুপথে প্রবেশ করিয়েছেন) যৌন কাজের সময় আপনি কি কনডম ব্যবহার করেছিলেন?	হ্যাঁ<br>না<br>কখনো প্রবেশমূলক যৌন কাজ করে নাই<br>কখনও কনডম ব্যবহার করে নাই হলে<br>উত্তর না দেয়া	১<br>২<br>৩<br>৪<br>৯৮		
২১৩	শেষবার গ্রহনমূলক/প্রবেশমূলক যে কোন পুরুষের সাথে পায়ুপথে যৌন কাজের সময় আপনি কি কনডম ব্যবহার করেছিলেন?	হ্যাঁ<br>না<br>উত্তর না দেয়া	১<br>২<br>৯৮		

সেকশন ৩ক ঃ পুরুষ সঙ্গীদের সাথে যৌন আচরন

প্রথমে আমি আপনার টাকা ছাড়া পুরুষ/হিজড়া সঙ্গীদের সাথে সেক্সের ব্যাপারে কথা বলব

নং	প্রশ্ন	কোডের ধরন		নির্দেশ	মন্তব্য
৩০০	গত এক মাসের মধ্যে আপনি কি কোন পুরুষ/হিজড়ার সাথে টাকা ছাড়া **(পায়ু পথে বা মুখে)** যৌনমিলন করেছেন?	হ্যাঁ<br>না	১<br>২	 → ৩০৬	
৩০১	গত এক মাসের মধ্যে আপনি কতজন ভিন্ন ভিন্ন পুরুষ/হিজড়া সঙ্গীর সাথে টাকা ছাড়া **(পায়ু পথে বা মুখে)** যৌনমিলন করেছেন?	সংখ্যা<br>জানি না<br>উত্তর না দেয়া	---<br>৯৭<br>৯৮		
৩০২	গত এক মাসের মধ্যে আপনি টাকা ছাড়া পুরুষ/হিজড়া সঙ্গীর সাথে কতবার **পায়ুপথে** যৌনমিলন করেছেন?	শূন্য<br>সংখ্যা<br>জানি না<br>উত্তর না দেয়া	০<br>---<br>৯৭<br>৯৮	→ ৩০৫	
৩০৩	গত এক মাসে যখন টাকা ছাড়া পুরুষ/হিজড়া সঙ্গীর সাথে **পায়ুপথে** সর্বশেষ যৌনমিলন করেছিলেন তখন কি কনডম ব্যবহার করেছিলেন?	হ্যাঁ<br>না<br>জানি না<br>উত্তর না দেয়া	১<br>২<br>৯৭<br>৯৮		
৩০৪	গত এক মাসের মধ্যে আপনি টাকা ছাড়া পুরুষ/হিজড়া সঙ্গীর সাথে যতবার **পায়ুপথে** যৌনমিলন করেছিলেন তখন কি হারে কনডম ব্যবহার করেছিলেন?<br>**(১-৩ পড়ে শুনান)**	সব সময়<br>মাঝে মাঝে<br>কখনো না<br>উত্তর না দেয়া	১<br>২<br>৩<br>৯৮		
৩০৫	গত এক মাসের মধ্যে আপনি কতজন ভিন্ন ভিন্ন পুরুষ সঙ্গীর সাথে টাকা ছাড়া **মুখে (বীর্যপাত পর্যন্ত)** যৌনমিলন করেছিলেন?	শূন্য<br>সংখ্যা<br>জানি না<br>উত্তর না দেয়া	০<br>------<br>৯৭<br>৯৮		

এখন টাকা ছাড়া মহিলা সঙ্গীদের সাথে সেক্সের ব্যাপারে কথা বলব (হিজড়া বাদে)

নং	প্রশ্ন	কোডের ধরন		নির্দেশ	মন্তব্য
৩০৬	গত এক মাসে আপনি কি কোন মহিলার (হিজড়া নয়) সাথে টাকা ছাড়া **(যোনী পথে বা পায়ু পথে বা মুখে)** যৌনমিলন করেছেন?	হ্যাঁ<br>না	১<br>২	 → ৩১০	
৩০৭-ক	গত এক মাসের মধ্যে আপনি কতজন ভিন্ন ভিন্ন মহিলার সাথে টাকা ছাড়া **(যোনী পথে বা পায়ু পথে বা মুখে)** যৌনমিলন করেছেন?	সংখ্যা<br>জানি না<br>উত্তর না দেয়া	---<br>৯৭<br>৯৮		
৩০৭-খ	গত এক মাসের মধ্যে আপনি মহিলাদের সাথে টাকা	সংখ্যা	---		

নং	প্রশ্ন	কোডের ধরন	নির্দেশ	মন্তব্য
	ছাড়া কতবার **<u>যোনী পথে বা পায়ু পথে</u>** যৌনমিলন করেছেন?	জানি না ৯৭<br>উত্তর না দেয়া ৯৮		
৩০৮	শেষবার যখন টাকা ছাড়া মহিলা সঙ্গীর সাথে **<u>যোনীপথে অথবা পায়ুপথে</u>** যৌনমিলন করেছিলেন তখন কি কনডম ব্যবহার করেছিলেন?	হ্যাঁ ১<br>না ২<br>জানি না ৯৭<br>উত্তর না দেয়া ৯৮		
৩০৯	গত একমাসের মধ্যে আপনি যতবার টাকা ছাড়া মহিলা সঙ্গীর সাথে **<u>যোনীপথে অথবা পায়ুপথে</u>** যৌনমিলন করেছিলেন তখন কি হারে কনডম ব্যবহার করেছিলেন?<br>**(১-৩ পড়ে শুনান)**	সব সময় ১<br>মাঝে মাঝে ২<br>কখনো না ৩<br>উত্তর না দেয়া ৯৮		

এখন টাকা দিয়ে মহিলা যৌনসঙ্গীর সাথে সেক্সের ব্যপারে কথা বলব (হিজড়া বাদে)

নং	প্রশ্ন	কোডের ধরন	নির্দেশ	মন্তব্য
৩১০	গত একমাসের মধ্যে আপনি কি টাকা দিয়ে কোন মহিলার (হিজড়া নয়) সাথে **<u>(যোনী পথে বা পায়ু পথে বা মুখে)</u>** যৌনমিলন করেছেন?	হ্যাঁ ১<br>না ২	না → ৩১৫	
৩১১	গত একমাসের মধ্যে আপনি কতজন ভিন্ন ভিন্ন মহিলার সাথে টাকা দিয়ে **<u>(যোনী পথে বা পায়ু পথে বা মুখে)</u>** যৌনমিলন করেছেন?	সংখ্যা ---<br>জানি না ৯৭<br>উত্তর না দেয়া ৯৮		
৩১২	গত একমাসের মধ্যে আপনি কতবার টাকা দিয়ে **<u>যোনীপথে বা পায়ুপথে</u>** যৌনমিলন করেছিলেন?	শূন্য ০<br>সংখ্যা ---<br>জানি না ৯৭<br>উত্তর না দেয়া ৯৮	শূন্য → ৩১৫	
৩১৩	শেষবার যখন টাকা দিয়ে মহিলার সাথে **<u>যোনীপথে বা পায়ুপথে</u>** যৌনমিলন করেছিলেন তখন কি কনডম ব্যবহার করেছিলেন?	হ্যাঁ ১<br>না ২<br>জানি না ৯৭<br>উত্তর না দেয়া ৯৮		
৩১৪	গত একমাসের মধ্যে আপনি যতবার টাকা দিয়ে মহিলাদের সাথে **<u>যোনীপথে বা পায়ুপথে</u>** যৌনমিলন করেছিলেন তখন কি হারে কনডম ব্যবহার করেছিলেন?<br>**(১-৩ পড়ে শুনান)**	সব সময় ১<br>মাঝে মাঝে ২<br>কখনো না ৩<br>উত্তর না দেয়া ৯৮		

এখন টাকা দিয়ে পুরুষ যৌনসঙ্গীর সাথে সেক্সের কথা বলব (হিজড়া বাদে)

নং	প্রশ্ন	কোডের ধরন	নির্দেশ	মন্তব্য
৩১৫	গত এক মাসের মধ্যে আপনি কি টাকা দিয়ে কোন পুরুষের (হিজড়া নয়) সাথে **(পায়ু পথে বা মুখে)** যৌনমিলন করেছেন?	হ্যাঁ ১<br>না ২	না → ৩২১	
৩১৬	গত একমাসের মধ্যে আপনি কতজন ভিন্ন ভিন্ন পুরুষের সাথে টাকা দিয়ে **(পায়ু পথে বা মুখে)** যৌনমিলন করেছেন?	সংখ্যা ---<br>জানি না ৯৭<br>উত্তর না দেয়া ৯৮		
৩১৭	গত একমাসের মধ্যে আপনি কতবার টাকা দিয়ে **পায়ু পথে** যৌনমিলন করেছিলেন?	শূন্য ০<br>সংখ্যা ---<br>জানি না ৯৭<br>উত্তর না দেয়া ৯৮	শূন্য → ৩২০	
৩১৮	শেষবার যখন টাকা দিয়ে পুরুষ সঙ্গীর সাথে **পায়ু পথে** যৌনমিলন করেছিলেন তখন কি কনডম ব্যবহার করেছিলেন?	হ্যাঁ ১<br>না ২<br>জানি না ৯৭<br>উত্তর না দেয়া ৯৮		
৩১৯	গত একমাসের মধ্যে আপনি যতবার টাকা দিয়ে পুরুষ সঙ্গীর সাথে **পায়ু পথে** যৌনমিলন করেছিলেন তখন কি হারে কনডম ব্যবহার করেছিলেন?	সব সময় ১<br>মাঝে মাঝে ২<br>কখনো না ৩		

নং	প্রশ্ন	কোডের ধরন	নির্দেশ	মন্তব্য
	**(১-৩ পড়ে শুনান)**	উত্তর না দেয়া ৯৮		
৩২০	গত একমাসের মধ্যে আপনি টাকা দিয়ে কতজন ভিন্ন ভিন্ন পুরুষের সাথে **মুখে (বীর্যপাত পর্যন্ত)** যৌনমিলন করেছেন?	শূন্য ০<br>সংখ্যা ---<br>জানি না ৯৭<br>উত্তর না দেয়া ৯৮		

এখন আমি টাকা দিয়ে হিজড়া যৌনসঙ্গীর সাথে সেক্সের কথা বলব

নং	প্রশ্ন	কোডের ধরন	নির্দেশ	মন্তব্য
৩২১	গত একমাসের মধ্যে আপনি কি টাকা দিয়ে কোন হিজড়ার সাথে **(পায়ু পথে বা মুখে)** যৌনমিলন করেছেন?	হ্যাঁ ১<br>না ২	→ ৩২৭	
৩২২	গত একমাসের মধ্যে আপনি টাকা দিয়ে কতজন ভিন্ন ভিন্ন হিজড়ার সাথে **(পায়ু পথে বা মুখে)** যৌনমিলন করেছেন?	সংখ্যা ---<br>জানি না ৯৭<br>উত্তর না দেয়া ৯৮		
৩২৩	গত একমাসের মধ্যে আপনি টাকা দিয়ে কতজন ভিন্ন ভিন্ন হিজড়ার সাথে **পায়ুপথে** যৌনমিলন করেছেন?	শূন্য ০<br>সংখ্যা ---<br>জানি না ৯৭<br>উত্তর না দেয়া ৯৮	→ ৩২৬	
৩২৪	শেষবার যখন টাকা দিয়ে হিজড়ার সাথে **পায়ুপথে** যৌনমিলন করেছিলেন তখন কি কনডম ব্যবহার করেছিলেন?	হ্যাঁ ১<br>না ২<br>জানি না ৯৭<br>উত্তর না দেয়া ৯৮		
৩২৫	গত একমাসের মধ্যে আপনি যতবার টাকা দিয়ে হিজড়াদের সাথে **পায়ুপথে** যৌনমিলন করেছিলেন তখন কি হারে কনডম ব্যবহার করেছিলেন?<br>**(১-৩ পড়ে শুনান)**	সব সময় ১<br>মাঝে মাঝে ২<br>কখনো না ৩<br>উত্তর না দেয়া ৯৮		
৩২৬	গত একমাসের মধ্যে আপনি টাকা দিয়ে কতজন ভিন্ন ভিন্ন হিজড়ার সাথে **মুখে (বীর্যপাত পর্যন্ত)** যৌনমিলন করেছেন?	শূন্য ০<br>সংখ্যা ---<br>জানি না ৯৭<br>উত্তর না দেয়া ৯৮		

এখন আমি দলগত যৌনকাজ
(পায়ু/মুখে বা যৌনীপথে যৌনমিলন এবং একের অধিক সঙ্গী) সম্পর্কে বলব

নং	প্রশ্ন	কোডের ধরন	নির্দেশ	মন্তব্য
৩২৭	গত একমাসের মধ্যে আপনি কি কোন **দলগত** যৌনমিলনে অংশগ্রহন করেছিলেন?	হ্যাঁ ১<br>না ২<br>জানি না ৯৭<br>উত্তর না দেয়া ৯৮	→ ৩৩১	
৩২৮	শেষবার **দলগত** যৌনমিলনের সময় (আপনি সহ) কতজন সঙ্গী ছিল?	সংখ্যা-----------------<br>জানি না ৯৭<br>উত্তর না দেয়া ৯৮		
৩২৯	শেষবার **দলগত** যৌনমিলনে (আপনি ছাড়া) কতজন সঙ্গী কনডম ব্যবহার করেছেন?	সংখ্যা-----------------<br>জানিনা ৯৭<br>উত্তর না দেওয়া ৯৮		
৩৩০	শেষবার **দলগত** যৌনমিলনের সময় আপনি নিজে কি কনডম ব্যবহার করেছেন?	হ্যাঁ ১<br>না ২<br>জানি না ৯৭<br>উত্তর না দেয়া ৯৮		

সেকশন ৩খ ঃ এখন আমি অন্য কোন শহরে (দেশের ভিতরে অথবা দেশের বাহিরে) টাকা নিয়ে অথবা টাকা দিয়ে অথবা টাকা ছাড়া যৌন কাজ সম্পর্কে কথা বলব

নং	প্রশ্ন	কোডের ধরন	নির্দেশ	মন্তব্য
৩৩১	আপনি কি গত এক বছরে এই শহরের বাহিরে অন্য কোন শহরে গিয়েছেন?	হ্যাঁ ১<br>না ২<br>উত্তর না দেয়া ৯৮	২ → ৩৩৬	
৩৩২	আপনি কি গত এক বছরে এই শহরের বাহিরে অন্য কোন শহরে টাকা দিয়ে যৌনমিলন করেছেন?	হ্যাঁ ১<br>না ২<br>উত্তর না দেয়া ৯৮	২ → ৩৩৪	
৩৩৩	শেষবার গত এক বছরে এই শহরের বাহিরে অন্য কোন শহরে টাকা দিয়ে যৌনমিলনের সময় কনডম ব্যবহার করেছিলেন কি?	হ্যাঁ ১<br>না ২<br>উত্তর না দেয়া ৯৮		
৩৩৪	আপনি কি গত এক বছরে এই শহরের বাহিরে অন্য কোন শহরে টাকা ছাড়া যৌনমিলন করেছেন?	হ্যাঁ ১<br>না ২<br>উত্তর না দেয়া ৯৮	২ → ৩৩৬	
৩৩৫	শেষবার গত এক বছরে এই শহরের বাহিরে অন্য কোন শহরে টাকা ছাড়া যৌনমিলনের সময় কনডম ব্যবহার করেছিলেন কি?	হ্যাঁ ১<br>না ২<br>উত্তর না দেয়া ৯৮		
৩৩৬	আপনি কি গত এক বছরে বাংলাদেশের বাহিরে অন্য কোন দেশে গিয়েছিলেন?	হ্যাঁ ১<br>না ২<br>উত্তর না দেয়া ৯৮	২ → ৪০১	
৩৩৭	অপনি কি গত এক বছরে দেশের বাহিরে অন্য কোন দেশে টাকা দিয়ে যৌনমিলন করেছেন?	হ্যাঁ ১<br>না ২<br>উত্তর না দেয়া ৯৮	২ → ৩৩৯	
৩৩৮	শেষবার গত এক বছরে এই দেশের বাহিরে টাকা দিয়ে যৌনমিলনের সময় কনডম ব্যবহার করেছিলেন কি?	হ্যাঁ ১<br>না ২<br>উত্তর না দেয়া ৯৮		
৩৩৯	অপনি কি গত এক বছরে দেশের বাহিরে অন্য কোন দেশে টাকা ছাড়া যৌনমিলন করেছেন?	হ্যাঁ ১<br>না ২<br>উত্তর না দেয়া ৯৮	২ → ৪০১	
৩৪০	শেষবার গত এক বছরে এই দেশের বাহিরে টাকা ছাড়া যৌনমিলনের সময় কনডম ব্যবহার করেছিলেন কি?	হ্যাঁ ১<br>না ২<br>উত্তর না দেয়া ৯৮		

সেকশন ৪ ঃ এখন আমি পুর ষ কনডম এবং লুব্রিকেন্ট সম্পর্কে বলব

নং	প্রশ্ন	কোডের ধরন	নির্দেশ	মন্তব্য
৪০১	**(সাক্ষাৎকার গ্রহনকারী)**<br>কনডম দেখান এবং জিজ্ঞেস কর ন<br>**"আপনি বলতে পারেন এটা কি"?**	কনডম চিনতে পারা ১<br>কনডম চিনতে না পারা ২<br>উত্তর না দেয়া ৯৮	→ ৪০৬	
৪০২	আপনার সঙ্গে কি এখন কনডম আছে? দয়া করে আমাকে দেখান।	কনডম দেখাতে পারা ১<br>কনডম দেখাতে না পারা ২<br>উত্তর না দেয়া ৯৮		

নং	প্রশ্ন	কোডের ধরন		নির্দেশ	মন্তব্য
৪০৩	আপনি কি জানেন কোথায় বা কার নিকট থেকে কনডম পাওয়া যায়?<br>**(পড়ে শোনাবেন না)**<br>**(একাধিক উত্তর সম্ভব)**<br>**(উলেম্নখ করলে ১-এ গোল কর্‌ুন)**<br>**(উলেম্নখ না করলে ২-এ গোল কর্‌ুন)**	জানিনা কনডম কোথায় পাওয়া যায়<br>দোকান<br>ফার্মেসী<br>DIC<br>স্বাস্থ্যসেবা কেন্দ্র (DIC বাদে)<br>বার/গেস্টহাউস/হোটেল<br>বন্ধুবান্ধব<br>NGO কর্মী<br>যৌন সঙ্গি<br>অন্যান্য----------------------------<br>উত্তর না দেয়া	১ ২<br>১ ২<br>১ ২<br>১ ২<br>১ ২<br>১ ২<br>১ ২<br>১ ২<br>১ ২<br>১ ২<br>৯৮	→ যদি ১ হয়, ৪০৬ নং প্রশ্নে যান	
৪০৪	যখনই আপনার প্রয়োজন হয় তখনই কি কনডম পান?	হ্যাঁ<br>না<br>প্রয়োজন হয় না<br>কখনও কনডম ব্যবহার করে নাই<br>জানি না<br>উত্তর না দেয়া	১<br>২<br>৩<br>৪<br>৯৭<br>৯৮	→ ৪০৬<br>→ ৪০৫<br>}→ ৪০৬	
৪০৫	যখন আপনার কনডমের প্রয়োজন হয় তখন কনডম পান না কেন?<br><br>**(পড়ে শোনাবেন না)**<br>**(একাধিক উত্তর গ্রহণযোগ্য)**<br><br>**উলেম্নখ করলে ১-এ গোল কর্‌ুন**<br>**উলেম্নখ না করলে ২-এ গোল কর্‌ুন**	দাম বেশী<br>দোকান/ফার্মেসী দুরে<br>দোকান/ফার্মেসী বন্ধ<br>কনডম কিনতে লজ্জাবোধ<br>কোথায় পাওয়া যায় জানি না<br>বহন করতে চাই না<br>প্রয়োজন হয় না<br>জীবনে কখনো ব্যবহার করি নাই<br>অন্যান্য ----------------------------<br>জানি না<br>উত্তর না দেয়া	১ ২<br>১ ২<br>১ ২<br>১ ২<br>১ ২<br>১ ২<br>১ ২<br>১ ২<br>১ ২<br>৯৭<br>৯৮		
৪০৬	আপনি কি কখনো পায়ুপথে যৌনমিলনের সময় পিচ্ছিল জাতীয় কোন কিছু ব্যবহার করেছেন?<br>**(আমি বুঝাতে চাচ্ছি পায়ুপথে আপনি বা আপনার পুর্‌ুষ যৌনসঙ্গীর পুর্‌ুষ যৌনাঙ্গ সহজে প্রবেশের জন্য কোন পিচ্ছিল পদার্থ ব্যবহার করেছিলেন কিনা।)**	হ্যাঁ<br>না<br>জানি না<br>উত্তর না দেয়া	১<br>২<br>৯৭<br>৯৮	 → ৪০৯	
৪০৭	গত ১২ মাসে পায়ুপথে যৌনমিলনের সময় কোন ধরনের পিচ্ছিল জাতীয় পদার্থ ব্যবহার করেছিলেন?<br><br>**(একাধিক উত্তর গ্রহণযোগ্য)**<br>**(পড়ে শোনাবেন না)**	লালা<br>তৈল<br>Water based কনডম লুব্রিকেন্ট<br>এন্টিসেপ্টিক ক্রীম<br>সাধারন লোশন<br>ভ্যাসিলিন/জেলী/বিউটি ক্রীম<br>অন্যান্য ----------------------------<br>জানি না<br>উত্তর না দেয়া	১ ২<br>১ ২<br>১ ২<br>১ ২<br>১ ২<br>১ ২<br>১ ২<br>৯৭<br>৯৮		
৪০৮	শেষবার (গত ১২ মাসে) যখন পিচ্ছিল জাতীয় কিছু ব্যবহার করে যৌনমিলন করেছিলেন তখন কি কনডম ব্যবহার করেছিলেন?	হ্যাঁ<br>না<br>জানি না<br>উত্তর না দেয়া	১<br>২<br>৯৭<br>৯৮		
৪০৯	এমন কিছু লুব্রিকেন্ট আছে যা মূলত কনডম এর সাথে ব্যবহারের জন্য তৈরী করা হয়। আপনি কি এমন লুব্রিকেন্ট এর নাম শুনেছেন?	হ্যাঁ<br>না<br>জানি না<br>উত্তর না দেয়া	১<br>২<br>৯৭<br>৯৮	 → ৪১৪<br>→ ৪১৪<br>→ ৪১৪	
৪১০	আপনি কি আমাকে ঐ জাতীয় লুব্রিকেন্ট এর নাম বলতে পারবেন?	হ্যাঁ<br>নাম লিখুন ---------------------------	১		

নং	প্রশ্ন	কোডের ধরন	নির্দেশ	মন্তব্য
		না ২<br>জানি না ৯৭<br>উত্তর না দেয়া ৯৮		
৪১১	গত ১ মাসের মধ্যে কি হারে আপনি কনডমের জন্য তৈরী লুব্রিকেন্ট এবং কনডম **<u>একসাথে</u>** পায়ুপথে যৌনমিলনের সময় ব্যবহার করেছেন?	সব সময় ১<br>মাঝে মাঝে ২<br>কখনো না ৩<br>জানি না ৯৭<br>উত্তর না দেয়া ৯৮<br>গত এক মাসে যৌনকাজ করে নাই ৯৯	→ ৪১৩<br>→ ৪১২<br>→ ৪১২<br>→ ৪১৪<br>→ ৪১৪<br>→ ৫০১	
৪১২	আপনি কেন **<u>মাঝে মাঝে অথবা কখনোই</u>** কনডমের জন্য বিশেষভাবে তৈরী লুব্রিকেন্ট এবং কনডম **<u>একসাথে</u>** ব্যবহার করেন না?<br><br>**(পড়ে শোনাবেন না)**<br>**(একাধিক উত্তর সম্ভব)**<br>**উলেণ্টখ করলে ১-এ গোল কর–ন**<br>**উলেণ্টখ না করলে ২-এ গোল কর–ন**	দাম বেশী ১ ২<br>কিনতে লজ্জা ১ ২<br>কোথায় পাওয়া যায় জানি না ১ ২<br>ব্যবহার করার দরকার নাই ১ ২<br>অন্য ক্রীম ব্যবহার করি ১ ২<br>সরবরাহ কম ১ ২<br>সহজে বহনযোগ্য নয় ১ ২<br>অন্যান্য ---------------------------- ১ ২<br>জানি না ৯৭<br>উত্তর না দেয়া ৯৮	→ ৪১৪	
৪১৩	কি কারনে আপনি **<u>সবসময়</u>** যৌনমিলনের সময় কনডমের সাথে বিশেষভাবে তৈরী লুব্রিকেন্ট ব্যবহার করেন?<br><br>**(পড়ে শোনাবেন না)**<br>**(একাধিক উত্তর সম্ভব)**<br>**উলেণ্টখ করলে ১-এ গোল কর–ন**<br>**উলেণ্টখ না করলে ২-এ গোল কর–ন**	ফোলা/ব্যথা কমানো/সহজে প্রবেশ ১ ২<br>বেশী অনুভূতি ১ ২<br>কনডম ফাটার ঝুঁকি কমানো ১ ২<br>STI/HIV/AIDS কে বাধা দেয়া ১ ২<br>অন্যান্য ---------------------------- ১ ২<br>জানি না ৯৭<br>উত্তর না দেয়া ৯৮		
৪১৪	গতমাসে কনডম ব্যবহার কালে কি আপনার কনডম ফেটেছিল?	হ্যাঁ ১<br>না ২<br>জানি না ৯৭<br>গত মাসে কনডম ব্যবহার করি নাই ৩<br>জীবনে কনডম ব্যবহার করি নাই ৪<br>উত্তর না দেয়া ৯৮<br>গত এক মাসে সেক্স করি নাই ৯৯	 <br><br>→ ৫০১<br>→ ৫০১<br>→ ৫০১<br>→ ৫০১	
৪১৫	গত ১ মাসে আপনি কোথা অথবা কার নিকট থেকে কনডম পেয়েছেন?<br><br>**(গত মাসে যারা কনডম ব্যবহার করেছে শুধুমাত্র তাদেরকেই এই প্রশ্ন জিজ্ঞাসা কর–ন)**<br><br>**পড়ে শোনাবেন না**<br>**(একাধিক উত্তর গ্রহণযোগ্য)**<br><br>**উলেণ্টখ করলে ১-এ গোল কর–ন**<br>**উলেণ্টখ না করলে ২-এ গোল কর–ন**	দোকান ১ ২<br>ফার্মেসী ১ ২<br>স্বাস্থ্য সেবা কেন্দ্র ১ ২<br>বার/গেষ্ট হাউস/হোটেল ১ ২<br>বন্ধুবান্ধব ১ ২<br>দালাল ১ ২<br>N.G.O কর্মী ১ ২<br>এক মাসের আগে কেনা ছিল ১ ২<br>যৌন সঙ্গি ১ ২<br>অন্যান্য ----------------------------- ১ ২<br>জানি না ৯৭<br>উত্তর না দেয়া ৯৮		

সেকশন ৫ ঃ এখন আমি যৌন রোগ সম্পর্কে বলব

নং	প্রশ্ন	কোডের ধরন	নির্দেশ	মন্ড্রব্য
৫০১	আপনি কি পুর–ষের এমন কোন রোগের **<u>লক্ষণ বা উপসর্গের</u>** কথা জানেন যা যৌনমিলনের মাধ্যমে ছড়ায়?<br>**(পড়ে শোনাবেন না)**<br>**একাধিক উত্তর সম্ভব**<br>**উলেণ্ঢখ করলে ১-এ গোল কর–ন**<br>**উলেণ্ঢখ না করলে ২-এ গোল কর–ন**	পুর–ষাঙ্গে পুঁজ/স্রাব ১ ২<br>প্রস্রাবে জ্বালাপোড়া ১ ২<br>পুরূষাঙ্গে ঘাঁ/ক্ষত ১ ২<br>কুচকিতে বাগী ১ ২<br>পায়ুপথে স্রাব ১ ২<br>পায়ুপথে ঘাঁ/ক্ষত ১ ২<br>অন্যান্য---------------------------- ১ ২<br>জানি না ৯৭<br>উত্তর না দেয়া ৯৮		
৫০২	গত ১২ মাসের মধ্যে আপনার কি মূত্রনালী থেকে স্রাব/পুঁজ বেরিয়েছে? (কামরস ব্যতিত তরল কিংবা আঠালো জাতীয় কিছু)	হ্যাঁ ১<br>না ২<br>জানি না ৯৭<br>উত্তর না দেয়া ৯৮		
৫০৩	গত ১২ মাসের মধ্যে আপনার কি পায়ূপথ থেকে স্রাব/পুঁজ (তরল কিংবা আঠালো জাতীয় কিছু) বেরিয়েছে?	হ্যাঁ ১<br>না ২<br>জানি না ৯৭<br>উত্তর না দেয়া ৯৮		
৫০৪	গত ১২ মাসের মধ্যে আপনার কি পুর–ষাঙ্গে ঘাঁ/ক্ষত হয়েছিল?	হ্যাঁ ১<br>না ২<br>জানি না ৯৭<br>উত্তর না দেয়া ৯৮		
৫০৫	প্রশ্ন নং ৫০২, ৫০৩, ৫০৪ দেখুন যদি যে কোন একটিতে হ্যাঁ হয় তবে **''১''**গোল কর–ন অন্যথায় **''২''** গোল কর–ন।	হ্যাঁ ১<br>না ২	→ ৫০৯	
৫০৬ক	শেষবার আপনার যখন যৌন রোগের লক্ষণ দেখা দিয়েছিল তখন প্রথমে কি করেছিলেন?<br><br>**(পড়ে শোনাবেন না)**<br>**(একটি উত্তর হবে)**	হাসপাতাল থেকে চিকিৎসা ১<br>ঔষধ বিক্রেতা থেকে চিকিৎসা ২<br>প্রাইভেট ডাক্তার দ্বারা চিকিৎসা ৩<br>প্রাইভেট ক্লিনিক থেকে চিকিৎসা ৪<br>এন জি ও ক্লিনিক থেকে চিকিৎসা ৫<br>ক্যানভাস/সনাতনী চিকিৎসা ৬<br>বন্ধুবান্ধব থেকে উপদেশ/চিকিৎসা ৭<br>নিজে নিজে চিকিৎসা ৮<br>কিছুই না ৯<br>অন্যান্য---------------------------- ১০<br>জানি না ৯৭<br>উত্তর না দেয়া ৯৮	১–৮ → ৫০৬খ জিজ্ঞাসা করুন<br>৯ → ৫০৯	
৫০৬খ	GbwRI wK¬wb‡Ki bvg			
৫০৭	শেষবার যৌনরোগের লক্ষণ দেখা দেয়ার কতদিন পর চিকিৎসা নিয়েছিলেন?<br>(যদি প্রথম দিনই চিকিৎসা নেয় তাহলে কোড ১ হবে)	দিন ---<br>জানি না ৯৭<br>উত্তর না দেয়া ৯৮		
৫০৮	শেষবার যখন চিকিৎসা নিয়েছিলেন তখন মোট কত টাকা খরচ হয়েছিল?<br>(ঔষধ এবং ডাক্তারের ফি সহ)	টাকা ----<br>মনে নাই/জানি না ৯৭<br>উত্তর না দেয়া ৯৮		
৫০৯	যৌনমিলনের মাধ্যমে যেসব রোগ ছড়ায় সেই রোগ যাতে আপনার না হয় সে জন্য আপনি কি করেন?<br><br>**(পড়ে শোনাবেন না)**<br>**(একাধিক উত্তর সম্ভব)**<br>**উলেণ্ঢখ করলে ১-এ গোল কর–ন**<br>**উলেণ্ঢখ না করলে ২-এ গোল কর–ন**	কিছুই না ১ ২<br>সাবান/যৌনমিলনের পর ডেটল/প্রস্রাব দ্বারা যৌনাঙ্গ ধোয়া ১ ২<br>সর্বদা কনডম ব্যবহার করা ১ ২<br>মাঝে মাঝে কনডম ব্যবহার ১ ২<br>সবসময় একজন/বিশ্বস্ড় সঙ্গীর সাথে যৌন কাজ ১ ২<br>মহিলা যৌন কর্মী পরিহারের চেষ্টা ১ ২<br>যৌন কাজের আগে সঙ্গী পরীক্ষা/পরিষ্কার সঙ্গীর সাথে সেক্স করা ১ ২	→ ৫১১<br>(যদি ১ হয়)	

নং	প্রশ্ন	কোডের ধরন	নির্দেশ	মন্তব্য
		অন্যান্য---------------------------- ১ ২<br>ঔষধ গ্রহন ১ ২<br>জানি না ৯৭<br>উত্তর না দেয়া ৯৮	→ ৫১০ জিজ্ঞাসা করুন	
৫১০	আপনি কোন ধরনের ঔষধ গ্রহন করেন?<br>**(৫০৯ প্রশ্নে ঔষধ গ্রহণ করে থাকলে ঔষধের নাম অবশ্যই আনতে হবে)**	ঔষধের নাম ------------------------<br>জানি না ৯৭<br>উত্তর না দেয়া ৯৮		
৫১১	গত এক মাসে এই শহরে যৌনরোগের (STI) সেবা দেয় একরম কোন NGO ক্লিনিকে গিয়েছিলেন কি?	হ্যাঁ ১<br>না ২<br>জানি না ৯৭<br>উত্তর না দেয়া ৯৮	→ ৬০১	
৫১২	যদি হ্যাঁ হয়, কোন ক্লিনিকে গিয়েছিলেন?<br>**(একাধিক উত্তর গ্রহনযোগ্য)**	NGO/ক্লিনিকের নাম:<br>১. ____________________<br>২. ____________________<br>৩.		

সেকশন ৬ ঃ এখন আমি AIDS সম্বন্ধে জ্ঞান, ঝুঁকি এবং প্রতিরোধ সম্পর্কে কথা বলব

নং	প্রশ্ন	কোডের ধরন	নির্দেশ	মন্তব্য
৬০১	আপনি কি কখনো HIV/AIDS রোগের নাম শুনেছেন?	হ্যাঁ ১<br>না ২<br>উত্তর না দেয়া ৯৮	→ ৭০১	
৬০২	প্রতিবার যৌনমিলনের সময় সঠিকভাবে কনডম ব্যবহারের মাধ্যমে মানুষ কি HIV/AIDS - এর ঝুঁকি কমাতে পারে?	হ্যাঁ ১<br>না ২<br>জানি না ৯৭<br>উত্তর না দেয়া ৯৮		
৬০৩	পায়ুপথে যৌনমিলন না করে মানুষ কি HIV/AIDS - এর ঝুঁকি কমাতে পারে?	হ্যাঁ ১<br>না ২<br>জানি না ৯৭<br>উত্তর না দেয়া ৯৮		
৬০৪	যোনী/পায়ুপথে যৌনমিলনের সময় প্রতিবার সঠিকভাবে কনডম ব্যবহারের মাধ্যমে মানুষ কি HIV/AIDS - এর ঝুঁকি কমাতে পারে?	হ্যাঁ ১<br>না ২<br>জানি না ৯৭<br>উত্তর না দেয়া ৯৮		
৬০৫	একাধিক যৌনসঙ্গী পরিহার করে কি কেউ HIV/AIDS -এর ঝুঁকি কমাতে পারে?	হ্যাঁ ১<br>না ২<br>জানি না ৯৭<br>উত্তর না দেয়া ৯৮		
৬০৬	মশার কামড় থেকে কি কোন মানুষ HIV/AIDS দ্বারা আক্রান্ড় হতে পারে?	হ্যাঁ ১<br>না ২<br>জানি না ৯৭<br>উত্তর না দেয়া ৯৮		
৬০৭	HIV/AIDS দ্বারা আক্রান্ড় লোকের সাথে একত্রে খাবার খেয়ে কেউ কি HIV/AIDS দ্বারা আক্রান্ড় হতে পারে?	হ্যাঁ ১<br>না ২<br>জানি না ৯৭<br>উত্তর না দেয়া ৯৮		
৬০৮	অন্যের ব্যবহৃত সূঁচ/সিরিঞ্জ ব্যবহার করলে কেউ কি HIV/AIDS দ্বারা আক্রান্ড় হতে পারে?	হ্যাঁ ১<br>না ২<br>জানি না ৯৭<br>উত্তর না দেয়া ৯৮		
৬০৯	আপনি কি মনে করেন যে কাউকে দেখেই বলা যাবে	হ্যাঁ ১		

নং	প্রশ্ন	কোডের ধরন	নির্দেশ	মন্ড়ব্য
	সে HIV/AIDS দ্বারা আক্রান্ড় ?	না ২<br>জানি না ৯৭<br>উত্তর না দেয়া ৯৮		
৬১০	আপনার নিজের যাতে HIV/AIDS না হয় তার জন্য আপনি কি করেন?<br>**(পড়ে শোনাবেন না)**<br>**(একাধিক উত্তর গ্রহনযোগ্য)**<br>**উলেণ্ঢখ করলে ১-এ গোল কর**ু**ন**<br>**উলেণ্ঢখ না করলে ২-এ গোল কর**ু**ন**	কিছুই না ১ ২<br>যৌনমিলনের পর ডেটল/প্রস্রাব দ্বারা যৌনাঙ্গ<br>ধোয়া ১ ২<br>সর্বদা কনডম ব্যবহার করা ১ ২<br>মাঝে মাঝে কনডম ব্যবহার ১ ২<br>ঔষধ গ্রহন ১ ২<br>সবসময় একজন/বিশ্বস্ড় সঙ্গির সাথে যৌন ১ ২<br>কাজ ১ ২<br>মহিলা যৌন কর্মী পরিহারের চেষ্টা<br>যৌন কাজের আগে সঙ্গি পরীক্ষা/পরিষ্কার ১ ২<br>সঙ্গির সাথে সেক্স করা ১ ২<br>অন্যান্য---------------------------- ৯৭<br>জানি না ৯৮<br>উত্তর না দেয়া	→ ৬১১<br>(যদি ১ হয়)	

গোপনীয় ভাবে HIV এর পরীক্ষা
(গোপনীয় বলতে বুঝায় যে আপনি নিজে ব্যতীত অন্য কেউ এর ফলাফল জানবে না)

নং	প্রশ্ন	কোডের ধরন	নির্দেশ	মন্ড়ব্য
৬১১	আপনি HIV/AIDS দ্বারা আক্রান্ড় কিনা তা যদি পরীক্ষা করে দেখতে চান তাহলে গোপনীয়ভাবে কোথায় করতে পারবেন তা কি জানেন?	হ্যাঁ ১<br>না ২<br>উত্তর না দেয়া ৯৮	→ ৭০১	
৬১২	(পরীক্ষার ফলাফল আমি জানতে চাইনা)<br>আপনি কি কখনো HIV পরীক্ষা করেছেন?	হ্যাঁ ১<br>না ২<br>জানি না ৯৭<br>উত্তর না দেয়া ৯৮	→ ৭০১	
৬১৩	হ্যাঁ হলে সর্বশেষ কোথায় পরীক্ষা করেছেন?			
৬১৪	আপনি কি স্বেচছায় HIV পরীক্ষা করিয়েছেন নাকি অন্য কেউ উৎসাহিত করেছে অথবা আপনাকে পরীক্ষা করতে হয়েছে?	স্বেচছায় ১<br>অন্য কেউ উৎসাহিত করেছিল ২<br>প্রয়োজন হয়েছিল ৩<br>উত্তর না দেয়া ৯৮	→ ৬১৭ জিজ্ঞাসা করুন<br>→ ৬১৬ জিজ্ঞাসা করবেন না<br>→ ৬১৫ জিজ্ঞাসা করবেন না	
৬১৫	কে উৎসাহিত করেছিল? **(একটি মাত্র উত্তর হবে)**			
৬১৬	কেন প্রয়োজন হয়েছিল? **(একটি মাত্র উত্তর হবে)**			
৬১৭	আমি ফলাফল জানতে চাই না, আপনি ঐ ফলাফল পেয়েছিলেন কি?	হ্যাঁ ১<br>না ২<br>জানি না ৯৭<br>উত্তর না দেয়া ৯৮		
৬১৮	সর্বশেষ কতদিন আগে আপনি ঐওঠ পরীক্ষা করিয়েছেন?	গত ১ বছরের মধ্যে ১<br>১ বছর আগে ২<br>জানি না ৯৭<br>উত্তর না দেয়া ৯৮		

সেকশন ৭ ঃ নির্যাতন সংক্রান্ড় তথ্য

নং	প্রশ্ন	কোডের ধরন	নির্দেশ	মন্ড্ব্য
৭০১	গত ১২ মাসে কেউ কি আপনাকে মারধোর করেছে?	হ্যাঁ ১<br>না ২<br>মনে নাই ৯৭<br>উত্তর না দেয়া ৯৮	 → ৭০৩<br>→ ৭০৩	
৭০২	(যদি কেউ মারধর করে থাকে তাহলে) কে আপনাকে মারধর করেছে?<br><br>**(পড়ে শোনাবেন না)**<br>**(একাধিক উত্তর সম্ভব)**<br>**উলেণ্ডখ করলে ১-এ গোল করুন**<br>**উলেণ্ডখ না করলে ২-এ গোল করুন।**	আইন শৃখংলা রক্ষাকারী বাহিনী ১ ২<br>মাস্ড়ান ১ ২<br>নতুন যৌন সঙ্গি ১ ২<br>নিয়মিত যৌন সঙ্গি ১ ২<br>মহলণ্ডার লোক ১ ২<br>পরিবার/আত্নীয় ১ ২<br>অন্যান্য------------------------- ১ ২<br>মনে নাই ৯৭<br>উত্তর না দেয়া ৯৮		
৭০৩	গত ১২ মাসে কেউ কি আপনার ইচ্ছার বির্ব্দ্ধে আপনার সাথে যৌনমিলন করেছে?	হ্যাঁ ১<br>না ২<br>মনে নাই ৯৭<br>উত্তর না দেয়া ৯৮	 → ৭০৫<br>→ ৭০৫	
৭০৪	গত ১২ মাসে কে বা কারা আপনার ইচছার বিরূদ্ধে জোরপূর্বক যৌনমিলন করেছে?<br><br>**(পড়ে শোনাবেন না)**<br>**(একাধিক উত্তর সম্ভব)**<br>**উলেণ্ডখ করলে ১-এ গোল করুন**<br>**উলেণ্ডখ না করলে ২-এ গোল করুন।**	আইন শৃখংলা রক্ষাকারী বাহিনী ১ ২<br>মাস্ড়ান ১ ২<br>নতুন যৌন সঙ্গি ১ ২<br>নিয়মিত যৌন সঙ্গি ১ ২<br>মহলণ্ডার লোক ১ ২<br>আত্নীয় ১ ২<br>অন্যান্য-------------------------- ১ ২<br>মনে নাই ৯৭<br>উত্তর না দেয়া ৯৮		
৭০৫	গত ১২ মাসের মধ্যে আপনি কি গ্রেপ্তার হয়েছিলেন?	হ্যাঁ ১<br>না ২<br>জানি না ৯৭<br>উত্তর না দেয়া ৯৮	→ ৮০১	
৭০৬	যদি হ্যাঁ হয়, কি কারনে গ্রেফতার হয়েছিলেন ?	১ . . . .<br>২ . . . .<br>৩		

সেকশন ৮ ঃ এখন আমি ঝুঁকি সম্পর্কিত কথা বলব

নং	প্রশ্ন	কোডের ধরন	নির্দেশ	মন্ড্ব্য
৮০১	আপনি কি মনে করেন আপনার HIV/AIDS -এর ঝুঁকি বেশী মাঝারী বা কম?	বেশী ১<br>মাঝারী ২<br>ঝুঁকি নেই/কম ৩<br>জানি না ৯৭<br>উত্তর না দেয়া ৯৮	→৮০২<br>→ ৮০২<br>→ ৮০৩<br>→ ৯০১<br>→ ৯০১	
৮০২	আপনি কেন মনে করেন আপনার HIV/AIDS হওয়ার <u>**বেশী বা মাঝারী**</u> ঝুঁকি রয়েছে?<br><br>**(পড়ে শোনাবেন না)**<br>**(একাধিক উত্তর সম্ভব)**<br><br>**উলেণ্ডখ করলে ১-এ গোল কর্ব্দন**<br>**উলেণ্ডখ না করলে ২-এ গোল কর্ব্দন**	ঝুঁকিপুর্ন কাজ ১ ২<br>পুনঃপুনঃ পায়ুপথে যৌনকাজ ১ ২<br>কনডম ব্যবহার না করা ১ ২<br>অনিয়মিত কনডম ব্যবহার ১ ২<br>সূঁচ/সিরিঞ্জ ভাগাভাগি ১ ২<br>অন্যান্য ------------------------------- ১ ২<br>জানি না ৯৭<br>উত্তর না দেয়া ৯৮	→ ৯০১	
৮০৩	আপনি কেন মনে করেন আপনার HIV/AIDS হওয়ার ঝুঁকি <u>**নাই অথবা কম?**</u><br>**(পড়ে শোনাবেন না)**<br>**(একাধিক উত্তর সম্ভব)**<br>**উলেণ্ডখ করলে ১-এ গোল কর্ব্দন**<br>**উলেণ্ডখ না করলে ২-এ গোল কর্ব্দন**	সর্বদা কনডম ব্যবহার ১ ২<br>পরিস্কার সঙ্গী/ খদ্দের ১ ২<br>স্বাস্থ্যবান সঙ্গী/ খদ্দের ১ ২<br>কখনও সূঁচ/সিরিঞ্জি ভাগাভাগী করি না ১ ২<br>মাঝে মাঝে সূঁচ/সিরিঞ্জ ভাগাভাগী করি ১ ২<br>অনিয়মিত কনডম ব্যবহার ১ ২<br>সবসময় একজন/বিশ্বস্ড় সঙ্গির সাথে যৌন কাজ ১ ২		

নং	প্রশ্ন	কোডের ধরন	নির্দেশ	মন্ড্ব্য
		অন্যান্য ---------------------------------- ১ ২<br>জানি না ৯৭<br>উত্তর না দেয়া ৯৮		

সেকশন ৯ ঃ কর্মসূচীতে অংশগ্রহণ

নং	প্রশ্ন	কোডের ধরন	নির্দেশ	মন্ড্ব্য
৯০১	আপনি কি কখনো **এন জি ও/সেল্ফ হেল্প গ্রুপ/সিবিও** পরিচালিত কোন এইডস প্রতিরোধ কর্মসূচীতে অংশগ্রহণ করেছিলেন?	হ্যাঁ ১<br>না ২<br>এই এলাকায় কোন এন জি ও/সেল্ফ হেল্প গ্রুপ/সিবিও নাই ৯৬<br>মনে নাই/জানি না ৯৭<br>উত্তর না দেয়া ৯৮	907	
৯০২	আপনি সর্বশেষ কত মাস আগে **এন জি ও/সেল্ফ হেল্প গ্রুপ/সিবিও** পরিচালিত কোন এইডস প্রতিরোধ কর্মসূচীতে অংশগ্রহণ করেছিলেন?	. . . . মাস<br>০ (যদি ১ মা‡সর মধ্যে হয়) ৯৬<br>মনে নাই/জানি না ৯৭<br>উত্তর না দেয়া ৯৮		
৯০৩	**এন জি ও/সেল্ফ হেল্প গ্রুপ/সিবিও** পরিচালিত কোন এইডস প্রতিরোধ কর্মসূচীতে আপনি কত দিন যাবত যুক্ত আছেন?	. . . . মাস<br>০ (যদি ১ মা‡সর মধ্যে হয়) ৯৬<br>মনে নাই/জানি না ৯৭<br>উত্তর না দেয়া ৯৮		
৯০৪	যদি কর্মসূচীতে অংশ গ্রহন করে থাকেন, গত মাসে কতবার অংশ নিয়েছেন?	শূন্য ০<br>সংখ্যা -----<br>জানি না ৯৭<br>উত্তর না দেয়া ৯৮		
৯০৫	যদি হ্যাঁ হয় কি ধরনের কর্মসূচীতে অংশগ্রহণ করেছিলেন (সারাজীবনে)?<br>(একাধিক উত্তর গ্রহন যোগ্য)<br>**(পড়ে শোনাবেন না)**<br>উলেণ্ঢখ করলে ১ গোল করুন<br>উলেণ্ঢখ না করলে ২ গোল করুন	সূঁচ/সিরিঞ্জ বিনিময় কর্মসূচী ১ ২<br>শিক্ষামূলক কার্যক্রম ১ ২<br>কনডম পেয়েছেন ১ ২<br>যৌনরোগের জন্য চিকিৎসা পেয়েছেন ১ ২<br>IHC তে অংশগ্রহণ ১ ২<br>DIC তে অংশগ্রহণ ১ ২<br>VCT তে অংশগ্রহণ ১ ২<br>অন্যান্য---------------------------------- ১ ২<br>জানি না ৯৭<br>উত্তর না দেয়া ৯৮		
৯০৬	আপনি এ কর্মসূচী থেকে (গত ১২ মাসে) কিভাবে উপকৃত হয়েছিলেন?<br><br>**(পড়ে শোনাবেন না) (একাধিক উত্তর সম্ভব)**<br>**উলেণ্ঢখ করলে ১-এ গোল করুন**<br>**উলেণ্ঢখ না করলে ২-এ গোল করুন**	আচরন পরিবর্তন করতে সাহায্য করেছিল ১ ২<br>মূল্যবান তথ্য ছিল কিন্তু আচরন পরিবর্তন হয়নি ১ ২<br>এইচআইভি/এইডস/এসটিডি/নিরাপদ সেক্স ও কনডমের সঠিক ব্যবহার সম্পর্কে জানতে পেরেছি ১ ২<br>তথ্য সহজবোধ্য ছিল না ১ ২<br>আমাদের প্রয়োজনের সাথে মিল ছিল না ১ ২<br>অন্যান্য---------------------------------- ১ ২		

সেকশন ১০ ঃ সাক্ষাতের স্থান এবং বিবিধ

নং	প্রশ্ন	কোডের ধরন	নির্দেশ	মন্তব্য
৯০৭	আপনি আপনার জনগোষ্ঠির বন্ধুদের সাথে কোথায় দেখা করেন?<br><br>**(পড়ে শোনাবেন না)**<br>**(একাধিক উত্তর সম্ভব)**<br>**(উলেস্নখ করলে ১-এ গোল করম্নন)**<br>**(উলেস্নখ না করলে ২-এ গোল করম্নন)**	ক্রুজিং স্পটে ১ ২<br>বাসায় ১ ২<br>ক্লাবে ১ ২<br>পার্টিতে ১ ২<br>চায়ের দোকান ১ ২<br>রাস্তায় ১ ২<br>অন্যান্য.. ১ ২<br>মনে নাই/জানি না ৯৭<br>উত্তর না দেয়া ৯৮		
৯০৮	আপনি কিভাবে পুরম্নষ সঙ্গীর সাথে যৌন কাজের জন্য যোগাযোগ করেন?<br><br>**(পড়ে শোনাবেন না)**<br>**(একাধিক উত্তর সম্ভব)**<br>**(উলেস্নখ করলে ১-এ গোল করম্নন)**<br>**(উলেস্নখ না করলে ২-এ গোল করম্নন)**	ক্রুজিং স্পটে ১ ২<br>ফোনের মাধ্যমে ১ ২<br>ইন্টারনেটের মাধ্যমে ১ ২<br>বন্ধু-বান্ধবের মাধ্যমে ১ ২<br>দালালের মাধ্যমে ১ ২<br>ক্লাবে ১ ২<br>পার্টিতে ১ ২<br>চায়ের দোকান ১ ২<br>রাস্তায় ১ ২<br>অন্যান্য.. ১ ২<br>মনে নাই/জানি না ৯৭<br>উত্তর না দেয়া ৯৮		
৯০৯	যৌন আচরনের উপর ভিত্তি করে আপনি আপনাকে কিভাবে পরিচয় দিতে পছন্দ করেন?<br><br>**(পড়ে শোনাবেন না)**<br>**(একটি উত্তর হবে)**	পুরম্নষ/ম্যানলি/সাধারন মানুষ ১<br>পারিখ ২<br>সিনেমার নায়ক ৩<br>পান্থি ৪<br><br>সিনেমার নায়িকা ৫<br>গে ৬<br>কতি ৭<br>দো-পারাটা ৮<br>মেয়ে/নারী ৯<br>অন্যান্য.. ১০<br>জানি না ৯৭<br>উত্তর না দেয়া ৯৮	(১–৪) → ধন্যবাদ দিয়ে সাক্ষাৎকার সমাপ্ত করম্নন।<br><br>(৫–৯) → ৯১০	
৯১০	আপনি কি কখনও মেয়েলি ভাব আনার জন্য কোন প্রকার ঔষধ সেবন করেছেন?	হ্যাঁ ১<br>না ২ →<br>মনে নাই ৯৭<br>উত্তর না দেয়া ৯৮	ধন্যবাদ দিয়ে সাক্ষাৎকার সমাপ্ত করম্নন।	
৯১১	যদি নিয়ে থাকেন, গত তিন মাসে নিয়েছেন কি?	হ্যাঁ ১<br>না ২ →<br>মনে নাই ৯৭<br>উত্তর না দেয়া ৯৮	ধন্যবাদ দিয়ে সাক্ষাৎকার সমাপ্ত করম্নন।	

নং	প্রশ্ন	কোডের ধরন	নির্দেশ	মন্ড়ব্য
৯১২	গত তিন মাসে কি কি ঔষধ সেবন করেছেন?<br><br>**(পড়ে শোনাবেন না)**<br>**(একাধিক উত্তর সম্ভব)**<br>**(উলেণ্ডখ করলে ১-এ গোল করুন)**<br>**(উলেণ্ডখ না করলে ২-এ গোল করুন)**	সুখী (Shukhi) ১ ২<br>সিলেস্ট-২১ (Cilest-21) ১ ২<br>ডেসোলন (Desolon) ১ ২<br>লাইনেস (Lynes) ১ ২<br>মারভেলন (Marvelon) ১ ২<br>ওভস্টেট (Ovostat) ১ ২<br>ফেমিকন (Femecon) ১ ২<br>নরডেট-২৮ (Nordat-28) ১ ২<br>এমকন (Emcon) ১ ২<br>পোসটিনর-২ (Postinor-2) ১ ২<br>মিনিকন (Minicon) ১ ২<br>ওভাকন (Ovacon) ১ ২<br>অন্যান্য.. ১ ২<br>মনে নাই/জানি না ৯৭<br>উত্তর না দেয়া ৯৮		
৯১৩	গত তিন মাসে ঐসকল ঔষধ সেবনের কারণগুলো কি?<br><br>**(পড়ে শোনাবেন না)**<br>**(একাধিক উত্তর সম্ভব)**<br>**(উলেণ্ডখ করলে ১-এ গোল করুন)**<br>**(উলেণ্ডখ না করলে ২-এ গোল করুন)**	বুকের মাপ বৃদ্ধি করার জন্য ১ ২<br>উরু/পাছা বৃদ্ধি/মসৃন করার জন্য ১ ২<br>শরীরের কোমলতা বৃদ্ধি করার জন্য ১ ২<br>দাড়ি/গোঁফ না গজানোর জন্য ১ ২<br><br>অন্যান্য.. ১ ২<br>মনে নাই/জানি না ৯৭<br>উত্তর না দেয়া ৯৮		
৯১৪	আপনি গত তিন মাসে কি হারে ঐসকল ঔষধ সেবন করেছেন?<br><br>(পড়ে শোনান)	প্রতিদিন ১<br>সপ্তাহে একাধিক বার ২<br>সপ্তাহে একবার ৩<br>মাসে একবার ৪<br>মনে নাই/জানি না ৯৭<br>উত্তর না দেয়া ৯৮		

আপনার ম□ল্যবান সময় দিয়ে সহযোগীতা করার জন্য অসংখ্য ধন্যবাদ।

= < =
