# Supplementary material for: Changes in condom use among males who have sex with males (MSM): Measuring the effect of HIV prevention programme in Dhaka city
Source: PLoS One. 2020 Jul 24;15(7):e0236557. doi: 10.1371/journal.pone.0236557 (PMC7380615; doi:10.1371/journal.pone.0236557)
Supplement: S1 File — (ZIP) [file pone.0236557.s001.zip › MSM_ BSS Midline Bangla Questionnaire_2013.pdf]

প্রশ্নপত্রের ID নম্বর [ ]

এইচআইভি ঝুঁকি আচরণগত সার্ভে ফর্ম ২০১৩
এম এস এম (MSM)

আমি (নাম) icddr,b (কলেরা হাসপাতাল) এর পক্ষ থেকে একটি জরীপ প্রকল্পে কাজ করছি। এইডস নামক রোগ যাতে না হয় সে ব্যাপারে মানুষকে কিভাবে সাহায্য করা যায় আমরা তা জানার চেষ্টা করছি। এ ব্যাপারে আপনাকে কিছু একান্ত ব্যক্তিগত প্রশ্ন জিজ্ঞাসা করা প্রয়োজন। আপনি আমাকে যে সমস্ত তথ্য দিবেন তা শুধুমাত্র গবেষণার প্রয়োজনে ব্যবহার করা হবে। আপনার নাম অথবা ঠিকানা কোথাও লিপিবদ্ধ করা হবে না। সাক্ষাৎকার প্রদান প্রত্যাখান করা অথবা সাক্ষাৎকার শুরু করার পর আপনি যে কোন সময়ে সাক্ষাৎকার প্রদান বন্ধ করে দিতে পারবেন। আপনি আমাকে যা বলবেন তা সম্পূর্ণভাবে গোপন রাখা হবে। কারন আমরা আন্তরিক ভাবে চাই, বাংলাদেশে আপনাদের মধ্যে কারো যাতে HIV সংক্রমন বা AIDS না হয়। আপনি যদি সাক্ষাৎকার দিতে রাজী থাকেন, তা হলে সম্পূর্ণ সত্য কথা বলা অত্যন্ত গুরুত্বপূর্ন। আমরা কি তাহলে সাক্ষাৎকার শুরু করতে পারি?

হ্যাঁ না

যদি না হয়, কেন (একটি মাত্র উত্তর গ্রহণযোগ্য)? .. ..

অভিভাবক / সিনিয়র কমিউনিটি সহযোগীর সম্মতি (শুধুমাত্র তাদের জন্য প্রযোজ্য যারা ১৫ বছর থেকে ১৮ বছরের কম বয়সী)

আপনি যদি এই এমএসএম/এমএসডবিণ্টউ/হিজড়াকে এই গবেষণায় অংশগ্রহণ করার অনুমতি দিতে সম্মত থাকেন, তাহলে নিচের নির্দিষ্ট স্থানে আপনার স্বাক্ষর বা বাম বৃদ্ধাঙ্গুলীর টিপসই দিন।

______________________________ ____________

অভিভাবক / সিনিয়র কমিউনিটি সহযোগী এর স্বাক্ষর বা বাম বৃদ্ধাঙ্গুলীর টিপসই তারিখ

জরীপ এলাকা :----------------------------------

ক্লাষ্টার কোড ঃ [ ]

থানা কোড ঃ [ ]

সাক্ষাৎকার গ্রহনকারীর কোড ঃ [ ] স্বাক্ষর ঃ [ ]

সাক্ষাৎকার গ্রহণকরার তারিখ :-- (দিন/মাস/বছর)

সাক্ষাৎকার শুরু করার সময় :--- (ঘন্টাঃমিনিট) (২৪ ঘন্টা)

সাক্ষাৎকার শেষ করার সময় :--- (ঘন্টাঃমিনিট) (২৪ ঘন্টা)

সাক্ষাৎকার গ্রহন কি সম্পন্ন হয়েছে?

হ্যাঁ [ ]

না [ ]

যদি না হয়, কেন? (একটি মাত্র উত্তর গ্রহণযোগ্য) .. ..

সুপারভাইজারের স্বাক্ষর .... তারিখ ঃ ....../....../........
(দিন/মাস/বছর)

সেকশন ১ঃ পটভূমি

নং	প্রশ্ন	কোডের ধরন	নির্দেশ	মন্ড্রব্য
১০১	আপনার বয়স কত? (পূর্ণ বছরে লিখুন)	বৎসর---------------		
১০২	এ যাবৎ আপনি কত (শ্রেনী) পর্যন্ড় লেখাপড়া করেছেন?	সম্পন্নকৃত বৎসর ------<br>১ বৎসরের কম ০০<br>উত্তর না দেয়া ৯৮<br>কখনও স্কুলে যায় নাই ৯৯		
১০৩	এই শহরে আপনি কতদিন যাবৎ বসবাস করছেন?	বৎসর ______<br>১ বৎসরের কম ০০<br>সারাজীবন ৯৬<br>জানি না/মনে নাই ৯৭<br>উত্তর না দেয়া ৯৮		
১০৪	গত মাসে আপনার মোট আয় কত?	টাকা --------<br>জানি না/মনে নাই ৯৭<br>উত্তর না দেয়া ৯৮		
১০৫	কিভাবে এই টাকা উপার্জন করেছেন?<br><br>(একাধিক উত্তর সম্ভব)<br>(র‌্যাংকিং কর¯œন)<br>(পড়ে শোনাবেন না)	র‌্যাংকিং<br>ব্যবসা ১ ----<br>চাকুরী ২ ----<br>ড্রাইভার ৩ ----<br>টিউশনি/শিক্ষকতা ৪ ----<br>পরিবার ৫ ----<br>অন্যান্য ----------------------------- ৬ ----		
১০৬	গত ১২ মাসে কি আপনি টাকার জন্য রক্ত বিক্রি করেছেন?	হ্যাঁ ১<br>না ২<br>জানি না/মনে নাই ৯৭<br>উত্তর না দেয়া ৯৮		
১০৭	কিছু লোক মজা করার জন্য বা নেশার জন্য অবৈধ মাদক দ্রব্য নেয়। গত ১২ মাসে আপনি কি **অবৈধ** মাদকদ্রব্য **(ঘুমের ট্যাবলেট ছাড়া)** নিয়েছেন?	হ্যাঁ ১<br>না ২<br>জানি না/মনে নাই ৯৭<br>উত্তর না দেয়া ৯৮	(২, ৯৭, ৯৮) → ১১১	
১০৮	যদি হ্যাঁ হয়, তবে কি কি অবৈধ মাদক দ্রব্য নিয়েছেন?<br><br>(পড়ে শোনাবেন না)<br>(একাধিক উত্তর সম্ভব)<br>(উলেণ্টখ করলে ১ -এ গোল কর¯œন)<br>(উলেণ্টখ না করলে ২-এ গোল কর¯œন)	মদ ১ ২<br>গাঁজা ১ ২<br>ফেন্সিডিল ১ ২<br>হেরোইন ১ ২<br>ইঞ্জেকশন (ব্র¯œপেনরফিন/পেথেডিন) ১ ২<br>ইয়াবা ১ ২<br>অন্যান্য ----------------------------- ১ ২		
১০৯	গত ১২ মাসের মধ্যে সর্বশেষ কবে অবৈধ মাদক দ্রব্য নিয়েছেন?	-------------------- দিন আগে		
১১০	গত ছয় মাসের মধ্যে কোন মাদক দ্রব্য বেশী গ্রহন করেছেন?<br><br>**একটি মাত্র উত্তর হবে**	গত ৬ মাসে মাদক গ্রহণ করে নাই ০<br>মদ ১<br>গাঁজা ২<br>ফেন্সিডিল ৩<br>হেরোইন ৪<br>ইনজেকশন (ব্র¯œপেনরফিন/পেথেডিন) ৫<br>ইয়াবা ৬<br>অন্যান্য ----------------------------- ৭		
১১১	কিছু লোক মজার জন্য বা নেশার জন্য ইনজেকশন নেয়। গত ১২ মাসে আপনি কি মজাকরার জন্য বা নেশার জন্য ইনজেকশন নিয়েছেন?	হ্যাঁ ১<br>না ২<br>জানি না/মনে নাই ৯৭<br>উত্তর না দেয়া ৯৮	(২, ৯৭, ৯৮) → ২০১	
১১২	কিছু লোক মজা করার জন্য বা নেশার জন্য ইনজেকশন নেয়। গত ২ মাসে আপনি কি নেশার জন্য ইনজেকশন নিয়েছেন ?	হ্যাঁ ১<br>না ২<br>Rvwb bv/g‡b bvB ৯৭<br>উত্তর না দেয়া ৯৮		

নং	প্রশ্ন	কোডের ধরন	নির্দেশ	মন্তব্য
১১৩	গত ১২ মাসে নেশার ইনজেকশন নেয়ার সময় **শেষবার** আপনি কি আপনার ব্যবহৃত সূঁচ/সিরিঞ্জ অন্য কাউকে দিয়েছেন বা অন্যের ব্যবহৃত সূঁচ/সিরিঞ্জ আপনি নিয়েছেন?	হ্যাঁ ১<br>না ২<br>জানি না/মনে নাই ৯৭<br>উত্তর না দেয়া ৯৮		

সেকশন ২ ঃ বিবাহ, সঙ্গী এবং যৌন ইতিহাস

নং	প্রশ্ন	কোডের ধরন	নির্দেশ	মন্তব্য
২০১	বর্তমানে আপনার বৈবাহিক অবস্থা কি?<br>**(পড়ে শোনাবেন)**<br>(একটি মাত্র উত্তর হবে)	বিবাহিত ১<br>অবিবাহিত ২<br>ডিভোর্সড ৩<br>বিপত্নীক ৪<br>আলাদা বসবাস ৫<br>উত্তর না দেয়া ৯৮	(২–৯৮) → ২০৩	
২০২	বর্তমানে আপনি কি আপনার স্ত্রীর সাথে বসবাস করছেন?	হ্যাঁ ১<br>না ২<br>উত্তর না দেয়া ৯৮		
২০৩	বর্তমানে আপনার স্ত্রী ছাড়া কি কোন নিয়মিত যৌনসঙ্গী আছে?	হ্যাঁ ১<br>না ২<br>উত্তর না দেয়া ৯৮	→ ২০৫	
২০৪	আপনার বর্তমান নিয়মিত যৌনসঙ্গী কে?<br>(একাধিক উত্তর সম্ভব)	পুরুষ ১ ২<br>মহিলা ১ ২<br>হিজড়া ১ ২<br>উত্তর না দেয়া ৯৮		
২০৫	কত বৎসর বয়সে আপনি প্রথম যৌনমিলন করেছেন? (পায়ু/যোনীপথে)	বৎসর ---<br>কখনো না ৯৬<br>জানি না/মনে নাই ৯৭<br>উত্তর না দেয়া ৯৮	→ ৩৩১	
২০৬ক	আপনার প্রথম যৌনসঙ্গী পুরুষ, মহিলা না হিজড়া ছিল?<br>(একটি মাত্র উত্তর হবে)	পুরুষ ১<br>মহিলা ২<br>হিজড়া ৩<br>জানি না/মনে নাই ৯৭<br>উত্তর না দেয়া ৯৮		
২০৬খ	আপনি কতদিন যাবৎ এমএসএম হিসেবে আছেন (বৎসর)	------- বছর<br>এক বছরের কম ০		
২০৭ক	আপনি কি কখনো টাকা নিয়ে অথবা বাধ্যতামূলক উপহার নিয়ে পুরুষ/হিজড়া খদ্দেরের সাথে <u>**পায়ুপথে**</u> যৌনমিলন করেছেন?	হ্যাঁ ১ →<br>না ২ →<br>উত্তর না দেয়া ৯৮	২০৭খ জিজ্ঞেস করে **MSW** প্রশ্ন পুরণ কর“ন<br>২০৭গ	
২০৭খ	আপনি সর্বশেষ কবে টাকা নিয়ে অথবা বাধ্যতামূলক উপহার নিয়ে পুরুষ/হিজড়া খদ্দেরের সাথে <u>**পায়ুপথে**</u> যৌনমিলন করেছেন?	. . . . মাস<br>১ মা‡সর মধ্যে ০০<br>জানি না/মনে নাই ৯৭<br>উত্তর না দেয়া ৯৮		
২০৭গ	আপনি সর্বশেষ কত মাস আগে <u>**পুর“ষের**</u> (হিজড়া নয়) সাথে (টাকা দিয়ে/ টাকা ছাড়া) <u>**পায়ুপথে**</u> যৌনমিলন করেছেন?	. . . . মাস<br>১ মা‡সর মধ্যে ০০<br>জানি না/মনে নাই ৯৭<br>উত্তর না দেয়া ৯৮		
২০৮	আপনি সর্বশেষ কত মাস আগে <u>**হিজড়ার**</u> সাথে (টাকা দিয়ে/টাকা ছাড়া) <u>**পায়ুপথে**</u> যৌনমিলন করেছেন?	. . . . মাস<br>১ মাসের মধ্যে ০০<br>হিজড়ার সাথে যৌনমিলন করে নাই ৯৬<br>জানি না/মনে নাই ৯৭<br>উত্তর না দেয়া ৯৮		

নং	প্রশ্ন	কোডের ধরন	নির্দেশ	মন্তব্য
২০৯	আপনি সর্বশেষ কত মাস আগে কোন মহিলার সাথে (হিজড়া নয়) (টাকা দিয়ে/টাকা ছাড়া) <u>**যোনী অথবা পায়ূপথে**</u> যৌনমিলন করেছেন?	. . . . মাস<br>১ মাসের মধ্যে ০০<br>মহিলার সাথে যৌনমিলন করে নাই ৯৬<br>জানি না/মনে নাই ৯৭<br>উত্তর না দেয়া ৯৮		
২১০	আপনি কি জীবনে কখনও **প্রবেশমূ□লক** (আপনার লিঙ্গ যৌনসঙ্গীর **পায়ূপথে** প্রবেশ করিয়েছেন) অথবা **গ্রহনম□লক** (যৌনসঙ্গী তার লিঙ্গ আপনার **পায়ূপথে** প্রবেশ করিয়েছে) যৌনমিলনে কনডম ব্যবহার করেছেন?	হ্যাঁ (গ্রহণমূলক যৌনমিলনে) ১<br>হ্যাঁ (প্রবেশমূলক যৌনমিলনে) ২<br>হ্যাঁ (গ্রহণমূলক এবং প্রবেশমূলক যৌনমিলনে) ৩<br>কখনও কনডম ব্যবহার করে নাই ৪<br>উত্তর না দেয়া ৯৮		
২১১	গত **১২** মাসের মধ্যে শেষবার গ্রহনমূলক (যৌনসঙ্গী তার লিঙ্গ আপনার **পায়ূপথে** প্রবেশ করিয়েছে) যৌন কাজের সময় আপনি কি কনডম ব্যবহার করেছিলেন?	হ্যাঁ ১<br>না ২<br>কখনো গ্রহনমূলক যৌন কাজ করে নাই ৩<br>কখনও কনডম ব্যবহার করে নাই ৪<br>উত্তর না দেয়া ৯৮		
২১২	গত **১২** মাসের মধ্যে শেষবার প্রবেশমূলক (আপনার লিঙ্গ যৌনসঙ্গীর **পায়ূপথে** প্রবেশ করিয়েছেন) যৌন কাজের সময় আপনি কি কনডম ব্যবহার করেছিলেন?	হ্যাঁ ১<br>না ২<br>কখনো প্রবেশমূলক যৌন কাজ করে নাই ৩<br>কখনও কনডম ব্যবহার করে নাই ৪<br>উত্তর না দেয়া ৯৮		
২১৩	শেষবার গ্রহনমূলক/প্রবেশমূলক যে কোন <u>**পুরুষের**</u> সাথে <u>**পায়ূপথে**</u> যৌন কাজের সময় আপনি কি কনডম ব্যবহার করেছিলেন?	হ্যাঁ ১<br>না ২<br>উত্তর না দেয়া ৯৮		

সেকশন ৩ক ঃ পুরুষ সঙ্গীদের সাথে যৌন আচরন

প্রথমে আমি আপনার টাকা ছাড়া পুরুষ/হিজড়া সঙ্গীদের সাথে সেক্সের ব্যাপারে কথা বলব

নং	প্রশ্ন	কোডের ধরন	নির্দেশ	মন্তব্য
২৯৮	গত ছয় মাসের মধ্যে আপনি কি কোন পুরুষ/হিজড়ার সাথে টাকা ছাড়া <u>**পায়ূপথে**</u> যৌনমিলন করেছেন?	হ্যাঁ ১<br>না ২	না → ৩০০	
২৯৯	গত ছয় মাসে যখন টাকা ছাড়া পুরুষ/হিজড়া সঙ্গীর সাথে <u>**পায়ূপথে**</u> সর্বশেষ যৌনমিলন করেছিলেন তখন কি কনডম ব্যবহার করেছিলেন?	হ্যাঁ ১<br>না ২<br>জানি না/মনে নাই ৯৭<br>উত্তর না দেয়া ৯৮		
৩০০	গত এক মাসের মধ্যে আপনি কি কোন পুরুষ/হিজড়ার সাথে টাকা ছাড়া <u>**(পায়ূপথে বা মুখে)**</u> যৌনমিলন করেছেন?	হ্যাঁ ১<br>না ২	না → ৩০৬	
৩০১	গত এক মাসের মধ্যে আপনি কতজন ভিন্ন ভিন্ন পুরুষ/হিজড়া সঙ্গীর সাথে টাকা ছাড়া <u>**(পায়ূপথে বা মুখে)**</u> যৌনমিলন করেছেন?	সংখ্যা ---<br>জানি না/মনে নাই ৯৭<br>উত্তর না দেয়া ৯৮		
৩০২	গত এক মাসের মধ্যে আপনি টাকা ছাড়া পুরুষ/হিজড়া সঙ্গীর সাথে কতবার <u>**পায়ূপথে**</u> যৌনমিলন করেছেন?	শূন্য ০<br>সংখ্যা ________<br>জানি না/মনে নাই ৯৭<br>উত্তর না দেয়া ৯৮	শূন্য → ৩০৫	
৩০৩	গত এক মাসে যখন টাকা ছাড়া পুরুষ/হিজড়া সঙ্গীর সাথে <u>**পায়ূপথে**</u> সর্বশেষ যৌনমিলন করেছিলেন তখন কি কনডম ব্যবহার করেছিলেন?	হ্যাঁ ১<br>না ২<br>জানি না/মনে নাই ৯৭<br>উত্তর না দেয়া ৯৮		
৩০৪	গত এক মাসের মধ্যে আপনি টাকা ছাড়া পুরুষ/হিজড়া সঙ্গীর সাথে যতবার <u>**পায়ূপথে**</u> যৌনমিলন করেছিলেন তখন কি হারে কনডম ব্যবহার করেছিলেন?<br>**(১-৩ পড়ে শুনান)**	সব সময় ১<br>মাঝে মাঝে ২<br>কখনো না ৩<br>উত্তর না দেয়া ৯৮		

নং	প্রশ্ন	কোডের ধরন	নির্দেশ	মন্ত্মব্য
৩০৫	গত এক মাসের মধ্যে আপনি কতজন ভিন্ন ভিন্ন পুরুষ সঙ্গীর সাথে টাকা ছাড়া **মুখে (বীর্যপাত পর্যন্ত্ম)** যৌনমিলন করেছিলেন?	শূন্য ০<br>সংখ্যা ------<br>জানি না/মনে নাই ৯৭<br>উত্তর না দেয়া ৯৮		

এখন টাকা ছাড়া মহিলা সঙ্গীদের (হিজড়া বাদে)/স্ত্রীর সাথে সেক্সের ব্যাপারে কথা বলব

নং	প্রশ্ন	কোডের ধরন	নির্দেশ	মন্ত্মব্য
৩০৬	গত এক মাসে আপনি কি কোন মহিলার (হিজড়া নয়) সাথে টাকা ছাড়া **(যোনী পথে বা পায়ুপথে)** যৌনমিলন করেছেন?	হ্যাঁ ১<br>না ২	→ ৩১০	
৩০৭ক	গত এক মাসের মধ্যে আপনি কতজন ভিন্ন ভিন্ন মহিলার সাথে টাকা ছাড়া **(যোনী পথে বা পায়ুপথে)** যৌনমিলন করেছেন?	সংখ্যা ---<br>জানি না/মনে নাই ৯৭<br>উত্তর না দেয়া ৯৮		
৩০৭খ	গত এক মাসের মধ্যে আপনি মহিলাদের সাথে টাকা ছাড়া কতবার **যোনী পথে** যৌনমিলন করেছেন?	শূন্য ০<br>সংখ্যা ---<br>জানি না/মনে নাই ৯৭<br>উত্তর না দেয়া ৯৮	→ ৩০৮ক	
৩০৭গ	গত এক মাসে শেষবার যখন টাকা ছাড়া মহিলা সঙ্গীর সাথে **যোনীপথে** যৌনমিলন করেছিলেন তখন কি কনডম ব্যবহার করেছিলেন?	হ্যাঁ ১<br>না ২<br>জানি না/মনে নাই ৯৭<br>উত্তর না দেয়া ৯৮		
৩০৮ক	গত একমাসের মধ্যে আপনি কতবার টাকা ছাড়া মহিলা সঙ্গীর সাথে **পায়ুপথে** যৌনমিলন করেছিলেন?	শূন্য ০<br>সংখ্যা ---<br>জানি না/মনে নাই ৯৭<br>উত্তর না দেয়া ৯৮	→ ৩০৯	
৩০৮খ	গত এক মাসে শেষবার যখন টাকা ছাড়া মহিলা সঙ্গীর সাথে **পায়ুপথে** যৌনমিলন করেছিলেন তখন কি কনডম ব্যবহার করেছিলেন?	হ্যাঁ ১<br>না ২<br>জানি না/মনে নাই ৯৭<br>উত্তর না দেয়া ৯৮		
৩০৯	গত একমাসের মধ্যে আপনি যতবার টাকা ছাড়া মহিলা সঙ্গীর সাথে **যোনীপথে অথবা পায়ুপথে** যৌনমিলন করেছিলেন তখন কি হারে কনডম ব্যবহার করেছিলেন? **(১-৩ পড়ে শুনান)**	সব সময় ১<br>মাঝে মাঝে ২<br>কখনো না ৩<br>উত্তর না দেয়া ৯৮		

এখন টাকা দিয়ে মহিলা যৌনসঙ্গীর (হিজড়া বাদে) সাথে সেক্সের ব্যাপারে কথা বলব

নং	প্রশ্ন	কোডের ধরন	নির্দেশ	মন্ত্মব্য
৩১০	গত একমাসের মধ্যে আপনি কি টাকা দিয়ে কোন মহিলার (হিজড়া নয়) সাথে **(যোনী পথে বা পায়ুপথে)** যৌনমিলন করেছেন?	হ্যাঁ ১<br>না ২	→ ৩১৫ক	
৩১১	গত একমাসের মধ্যে আপনি কতজন ভিন্ন ভিন্ন মহিলার সাথে টাকা দিয়ে **(যোনী পথে বা পায়ুপথে)** যৌনমিলন করেছেন?	সংখ্যা ---<br>জানি না/মনে নাই ৯৭<br>উত্তর না দেয়া ৯৮		
৩১২ক	গত একমাসের মধ্যে আপনি কতবার টাকা দিয়ে **যোনীপথে** যৌনমিলন করেছিলেন?	শূন্য ০<br>সংখ্যা ---<br>জানি না/মনে নাই ৯৭<br>উত্তর না দেয়া ৯৮	→ ৩১২গ	
৩১২খ	গত এক মাসে শেষবার যখন টাকা দিয়ে মহিলার সাথে **যোনীপথে** যৌনমিলন করেছিলেন তখন কি কনডম ব্যবহার করেছিলেন?	হ্যাঁ ১<br>না ২<br>জানি না/মনে নাই ৯৭<br>উত্তর না দেয়া ৯৮		
৩১২গ	গত একমাসের মধ্যে আপনি কতবার টাকা দিয়ে **পায়ুপথে** যৌনমিলন করেছিলেন?	শূন্য ০<br>সংখ্যা ---<br>জানি না/মনে নাই ৯৭	→ ৩১৩	

নং	প্রশ্ন	কোডের ধরন	নির্দেশ	মন্তব্য
		উত্তর না দেয়া ৯৮		
৩১২ঘ	গত এক মাসে **শেষবার** যখন টাকা দিয়ে মহিলার সাথে **পায়ূপথে** যৌনমিলন করেছিলেন তখন কি কনডম ব্যবহার করেছিলেন?	হ্যাঁ ১<br>না ২<br>জানি না/মনে নাই ৯৭<br>উত্তর না দেয়া ৯৮		
৩১৩	গত এক মাসের মধ্যে আপনি যতবার টাকা দিয়ে মহিলাদের সাথে **যোনীপথে বা পায়ূপথে** যৌনমিলন করেছিলেন তখন কি হারে কনডম ব্যবহার করেছিলেন? **(১-৩ পড়ে শুনান)**	সব সময় ১<br>মাঝে মাঝে ২<br>কখনো না ৩<br>উত্তর না দেয়া ৯৮		
৩১৪	গত এক মাসে আপনার সর্বশেষ যৌনসঙ্গী কে ছিল যার সাথে টাকা দিয়ে যৌনমিলন করেছিলেন?	রাস্তার যৌনকর্মী ১<br>হোটেল যৌনকর্মী ২<br>বাসার যৌনকর্মী ৩<br>যৌনপলণ্টীর যৌনকর্মী ৪<br>অন্যান্য (নির্দিষ্ট কর ুন - - -- -------) ৫<br>জানি না/মনে নাই ৯৭<br>উত্তর না দেয়া ৯৮		

এখন টাকা দিয়ে পুর ুষ যৌনসঙ্গীর (হিজড়া বাদে) সাথে সেক্সের কথা বলব

নং	প্রশ্ন	কোডের ধরন	নির্দেশ	মন্তব্য
৩১৫ক	গত ছয় মাসের মধ্যে আপনি কি টাকা দিয়ে কোন পুরুষের (হিজড়া নয়) সাথে **(পায়ূপথে)** যৌনমিলন করেছেন?	হ্যাঁ ১<br>না ২	→ ৩২১	
৩১৫খ	শেষবার (ছয় মাসের মধ্যে) যখন টাকা দিয়ে পুরুষ সঙ্গীর সাথে **পায়ূপথে** যৌনমিলন করেছিলেন তখন কি কনডম ব্যবহার করেছিলেন?	হ্যাঁ ১<br>না ২<br>জানি না/মনে নাই ৯৭<br>উত্তর না দেয়া ৯৮		
৩১৫গ	গত এক মাসের মধ্যে আপনি কি টাকা দিয়ে কোন পুরুষের (হিজড়া নয়) সাথে **(পায়ূপথে বা মুখে)** যৌনমিলন করেছেন?	হ্যাঁ ১<br>না ২	→ ৩২১	
৩১৬	গত এক মাসের মধ্যে আপনি কতজন ভিন্ন ভিন্ন পুরুষের সাথে টাকা দিয়ে **(পায়ূপথে বা মুখে)** যৌনমিলন করেছেন?	সংখ্যা ---<br>জানি না/মনে নাই ৯৭<br>উত্তর না দেয়া ৯৮		
৩১৭	গত এক মাসের মধ্যে আপনি কতবার টাকা দিয়ে পুরুষের সাথে **পায়ূপথে** যৌনমিলন করেছিলেন?	শূন্য ০<br>সংখ্যা ---<br>জানি না/মনে নাই ৯৭<br>উত্তর না দেয়া ৯৮	→ ৩২০	
৩১৮	গত এক মাসের মধ্যে শেষবার যখন টাকা দিয়ে পুরুষ সঙ্গীর সাথে **পায়ূপথে** যৌনমিলন করেছিলেন তখন কি কনডম ব্যবহার করেছিলেন?	হ্যাঁ ১<br>না ২<br>জানি না/মনে নাই ৯৭<br>উত্তর না দেয়া ৯৮		
৩১৯	গত এক মাসের মধ্যে আপনি যতবার টাকা দিয়ে পুরুষ সঙ্গীর সাথে **পায়ূপথে** যৌনমিলন করেছিলেন তখন কি হারে কনডম ব্যবহার করেছিলেন? **(১-৩ পড়ে শুনান)**	সব সময় ১<br>মাঝে মাঝে ২<br>কখনো না ৩<br>উত্তর না দেয়া ৯৮		
৩২০	গত এক মাসের মধ্যে আপনি টাকা দিয়ে কতজন ভিন্ন ভিন্ন পুরুষের সাথে **মুখে (বীর্যপাত পর্যন্ড়)** যৌনমিলন করেছেন?	শূন্য ০<br>সংখ্যা ---<br>জানি না/মনে নাই ৯৭<br>উত্তর না দেয়া ৯৮		

এখন আমি টাকা দিয়ে হিজড়া যৌনসঙ্গীর সাথে সেক্সের কথা বলব

নং	প্রশ্ন	কোডের ধরন	নির্দেশ	মন্তব্য
৩২১	গত এক মাসের মধ্যে আপনি কি টাকা দিয়ে কোন হিজড়ার সাথে **(পায়ুপথে বা মুখে)** যৌনমিলন করেছেন?	হ্যাঁ ১<br>না ২	না → ৩২৭	
৩২২	গত এক মাসের মধ্যে আপনি টাকা দিয়ে কতজন ভিন্ন ভিন্ন হিজড়ার সাথে **(পায়ুপথে বা মুখে)** যৌনমিলন করেছেন?	সংখ্যা ---<br>জানি না/মনে নাই ৯৭<br>উত্তর না দেয়া ৯৮		
৩২৩ক	গত এক মাসের মধ্যে আপনি টাকা দিয়ে কতজন ভিন্ন ভিন্ন হিজড়ার সাথে **পায়ুপথে** যৌনমিলন করেছেন?	শূন্য ০<br>সংখ্যা ---<br>জানি না/মনে নাই ৯৭<br>উত্তর না দেয়া ৯৮	শূন্য → ৩২৬	
৩২৩খ	গত এক মাসের মধ্যে আপনি টাকা দিয়ে **কতবার** ভিন্ন ভিন্ন হিজড়ার সাথে **পায়ুপথে** যৌনমিলন করেছেন?	শূন্য ০<br>সংখ্যা ---<br>জানি না/মনে নাই ৯৭<br>উত্তর না দেয়া ৯৮		
৩২৪	শেষবার যখন টাকা দিয়ে হিজড়ার সাথে **পায়ুপথে** যৌনমিলন করেছিলেন তখন কি কনডম ব্যবহার করেছিলেন?	হ্যাঁ ১<br>না ২<br>জানি না/মনে নাই ৯৭<br>উত্তর না দেয়া ৯৮		
৩২৫	গত এক মাসের মধ্যে আপনি যতবার টাকা দিয়ে হিজড়াদের সাথে **পায়ুপথে** যৌনমিলন করেছিলেন তখন কি হারে কনডম ব্যবহার করেছিলেন?<br>**(১-৩ পড়ে শুনান)**	সব সময় ১<br>মাঝে মাঝে ২<br>কখনো না ৩<br>উত্তর না দেয়া ৯৮		
৩২৬	গত এক মাসের মধ্যে আপনি টাকা দিয়ে কতজন ভিন্ন ভিন্ন হিজড়ার সাথে **মুখে (বীর্যপাত পর্যন্ড়)** যৌনমিলন করেছেন?	শূন্য ০<br>সংখ্যা ---<br>জানি না/মনে নাই ৯৭<br>উত্তর না দেয়া ৯৮		

এখন আমি দলগত যৌনকাজ সম্পর্কে বলব
(পায়ুপথে/মুখে বা যোনীপথে যৌনমিলন এবং একের অধিক সঙ্গী)

নং	প্রশ্ন	কোডের ধরন	নির্দেশ	মন্তব্য
৩২৭	গত একমাসের মধ্যে আপনি কি কোন **দলগত** যৌনমিলনে অংশগ্রহন করেছিলেন?	হ্যাঁ ১<br>না ২<br>জানি না/মনে নাই ৯৭<br>উত্তর না দেয়া ৯৮	না, ৯৭, ৯৮ → ৩৩১	
৩২৮	শেষবার **দলগত** যৌনমিলনের সময় (আপনি সহ) কতজন সঙ্গী ছিল?	সংখ্যা-----------------<br>জানি না/মনে নাই ৯৭<br>উত্তর না দেয়া ৯৮		
৩২৯	শেষবার **দলগত** যৌনমিলনে **(আপনি ছাড়া)** কতজন সঙ্গী কনডম ব্যবহার করেছেন?	সংখ্যা-----------------<br>জানি না/মনে নাই ৯৭<br>উত্তর না দেওয়া ৯৮		
৩৩০	শেষবার **দলগত** যৌনমিলনের সময় আপনি **নিজে** কি কনডম ব্যবহার করেছেন?	হ্যাঁ ১<br>না ২<br>জানি না/মনে নাই ৯৭<br>উত্তর না দেয়া ৯৮		

সেকশন ৩খ ঃ এখন আমি অন্য কোন শহরে (দেশের ভিতরে অথবা দেশের বাহিরে) টাকা নিয়ে অথবা টাকা দিয়ে অথবা টাকা ছাড়া যৌন কাজ সম্পর্কে কথা বলব

নং	প্রশ্ন	কোডের ধরন	নির্দেশ	মন্তব্য
৩৩১	আপনি কি গত এক বছরে এই শহরের বাহিরে অন্য কোন শহরে গিয়েছেন? (দেশের ভিতরে)	হ্যাঁ ১<br>না ২<br>উত্তর না দেয়া ৯৮	(না / উত্তর না দেয়া) → ৩৩৬	
৩৩২	আপনি কি গত ১২ মাসে এই শহরের বাহিরে অন্য কোন শহরে টাকা দিয়ে যৌনমিলন করেছেন?	হ্যাঁ ১<br>না ২<br>উত্তর না দেয়া ৯৮	(না / উত্তর না দেয়া) → ৩৩৪	
৩৩৩	শেষবার গত ১২ মাসে এই শহরের বাহিরে অন্য কোন শহরে টাকা দিয়ে যৌনমিলনের সময় কনডম ব্যবহার করেছিলেন কি?	হ্যাঁ ১<br>না ২<br>উত্তর না দেয়া ৯৮		
৩৩৪	আপনি কি গত ১২ মাসে এই শহরের বাহিরে অন্য কোন শহরে টাকা ছাড়া যৌনমিলন করেছেন?	হ্যাঁ ১<br>না ২<br>উত্তর না দেয়া ৯৮	(না / উত্তর না দেয়া) → ৩৩৬	
৩৩৫	শেষবার গত ১২ মাসে এই শহরের বাহিরে অন্য কোন শহরে টাকা ছাড়া যৌনমিলনের সময় কনডম ব্যবহার করেছিলেন কি?	হ্যাঁ ১<br>না ২<br>উত্তর না দেয়া ৯৮		
৩৩৬	আপনি কি গত ১২ মাসে বাংলাদেশের বাহিরে অন্য কোন দেশে গিয়েছিলেন?	হ্যাঁ ১<br>না ২<br>উত্তর না দেয়া ৯৮	(না / উত্তর না দেয়া) → ৪০১	
৩৩৬ক	দেশের বাহিরে গিয়ে থাকলে, কোন দেশ গিয়েছিলেন?	১. ....<br>২. ....<br>৩.		
৩৩৭	আপনি কি গত ১২ মাসে দেশের বাহিরে অন্য কোন দেশে টাকা দিয়ে যৌনমিলন করেছেন?	হ্যাঁ ১<br>না ২<br>উত্তর না দেয়া ৯৮	(না / উত্তর না দেয়া) → ৩৩৯	
৩৩৮	শেষবার গত ১২ মাসে এই দেশের বাহিরে টাকা দিয়ে যৌনমিলনের সময় কনডম ব্যবহার করেছিলেন কি?	হ্যাঁ ১<br>না ২<br>উত্তর না দেয়া ৯৮		
৩৩৯	আপনি কি গত ১২ মাসে দেশের বাহিরে অন্য কোন দেশে টাকা ছাড়া যৌনমিলন করেছেন?	হ্যাঁ ১<br>না ২<br>উত্তর না দেয়া ৯৮	(না / উত্তর না দেয়া) → ৪০১	
৩৪০	শেষবার গত ১২ মাসে এই দেশের বাহিরে টাকা ছাড়া যৌনমিলনের সময় কনডম ব্যবহার করেছিলেন কি?	হ্যাঁ ১<br>না ২<br>উত্তর না দেয়া ৯৮		

সেকশন ৪ ঃ এখন আমি পুরুষ কনডম এবং লুব্রিকেন্ট সম্পর্কে বলব

নং	প্রশ্ন	কোডের ধরন	নির্দেশ	মন্তব্য
৪০১	**(সাক্ষাৎকার গ্রহনকারী)**<br>কনডম দেখান এবং জিজ্ঞেস করুন<br>**"আপনি বলতে পারেন এটা কি"?**	কনডম চিনতে পারা ১<br>কনডম চিনতে না পারা ২<br>উত্তর না দেয়া ৯৮	(না পারা / উত্তর না দেয়া) → ৪০৬	
৪০২	আপনার সঙ্গে কি এখন কনডম আছে? দয়া করে আমাকে দেখান।	কনডম দেখাতে পারা ১<br>কনডম দেখাতে না পারা ২<br>উত্তর না দেয়া ৯৮		
৪০৩	আপনি কি জানেন কোথায় বা কার নিকট থেকে কনডম পাওয়া যায়?<br><br>(পড়ে শোনাবেন না)<br>(একাধিক উত্তর সম্ভব)<br>(উল্লেখ করলে ১-এ গোল করুন)<br>(উল্লেখ না করলে ২-এ গোল করুন)	জানিনা কনডম কোথায় পাওয়া যায় ১ ২<br>দোকান ১ ২<br>ফার্মেসী ১ ২<br>DIC ১ ২<br>ডিপো হোল্ডার ১ ২<br>স্বাস্থ্যসেবা কেন্দ্র (DIC বাদে) ১ ২<br>বার/গেস্টহাউস/হোটেল ১ ২<br>বন্ধুবান্ধব ১ ২<br>NGO কর্মী ১ ২	→ যদি ১ হয়, ৪০৬ নং প্রশ্নে যান	

নং	প্রশ্ন	কোডের ধরন	নির্দেশ	মন্তব্য
		যৌন সঙ্গী ১ ২<br>গত এক মাসে কনডম কিনি নাই ১ ২<br>কখনও কনডম ব্যবহার করি নাই ১ ২<br>কনডম পাই নাই ১ ২<br>অন্যান্য---------------------------- ১ ২<br>উত্তর না দেয়া ৯৮		
৪০৪	যখনই আপনার প্রয়োজন হয় তখনই কি কনডম পান?	হ্যাঁ ১<br>না ২<br>প্রয়োজন হয় না ৩<br>কখনও কনডম ব্যবহার করে নাই ৪<br>জানি না/মনে নাই ৯৭<br>উত্তর না দেয়া ৯৮	→ ৪০৬<br>→ ৪০৫<br>(৩–৯৮) → ৪০৬	
৪০৫	যখন আপনার কনডমের প্রয়োজন হয় তখন কনডম পান না কেন?<br><br>(পড়ে শোনাবেন না)<br>(একাধিক উত্তর গ্রহণযোগ্য)<br>(উল্লেখ করলে ১-এ গোল করুন)<br>(উল্লেখ না করলে ২-এ গোল করুন)	ডিআইসি অনেক দুরে ১ ২<br>পিয়ার এডুকেটর প্রয়োজনের সময় পাওয়া যায়না ১ ২<br>দাম বেশী ১ ২<br>দোকান/ফার্মেসী দুরে ১ ২<br>দোকান/ফার্মেসী বন্ধ ১ ২<br>কনডম কিনতে লজ্জাবোধ ১ ২<br>কোথায় পাওয়া যায় জানি না ১ ২<br>বহন করতে চাই না ১ ২<br>প্রয়োজন হয় না ১ ২<br>জীবনে কখনো ব্যবহার করি নাই ১ ২<br>অন্যান্য ---------------------------- ১ ২<br>জানি না/মনে নাই ৯৭<br>উত্তর না দেয়া ৯৮		
৪০৬	আপনি কি কখনো **পায়ূপথে** যৌনমিলনের সময় পিচ্ছিল জাতীয় কোন কিছু ব্যবহার করেছেন?<br>(আমি বুঝাতে চাচ্ছি **পায়ূপথে** আপনি বা আপনার পুরুষ যৌনসঙ্গীর পুরুষ যৌনাঙ্গ সহজে প্রবেশের জন্য কোন পিচ্ছিল পদার্থ ব্যবহার করেছিলেন কিনা।)	হ্যাঁ ১<br>না ২<br>জানি না/মনে নাই ৯৭<br>উত্তর না দেয়া ৯৮	(২–৯৮) → ৪০৯	
৪০৭	শেষবার **পায়ূপথে** যৌনমিলনের সময় কোন ধরনের পিচ্ছিল জাতীয় পদার্থ ব্যবহার করেছিলেন?<br><br>(একাধিক উত্তর গ্রহণযোগ্য)<br>(পড়ে শোনাবেন না)	লালা ১ ২<br>তৈল ১ ২<br>Water based কনডম লুব্রিকেন্ট ১ ২<br>এন্টিসেপ্টিক ক্রীম ১ ২<br>সাধারন লোশন ১ ২<br>ভ্যাসিলিন/পেট্রোলিয়াম জেলী/বিউটি ক্রীম ১ ২<br>শ্যাম্পু/ সাবান ১ ২<br>অন্যান্য ------------------------ ১ ২<br>শেষবার লুব্রিকেন্ট ব্যবহার করে নাই ১ ২<br>জানি না/মনে নাই ৯৭<br>উত্তর না দেয়া ৯৮	→ ৪০৯ (শেষবার লুব্রিকেন্ট ব্যবহার করে নাই)	
৪০৮	শেষবার যখন পিচ্ছিল জাতীয় কিছু ব্যবহার করে যৌনমিলন করেছিলেন তখন কি কনডম ব্যবহার করেছিলেন?	হ্যাঁ ১<br>না ২<br>জানি না/মনে নাই ৯৭<br>উত্তর না দেয়া ৯৮		
৪০৯	এমন কিছু লুব্রিকেন্ট আছে যা মূলত কনডম এর সাথে ব্যবহারের জন্য তৈরী করা হয়। আপনি কি এমন লুব্রিকেন্ট এর নাম শুনেছেন?	হ্যাঁ ১<br>না ২<br>জানি না/মনে নাই ৯৭	 → ৪১৪<br>→ ৪১৪	

নং	প্রশ্ন	কোডের ধরন	নির্দেশ	মন্তব্য
		উত্তর না দেয়া ৯৮	→ ৪১৪	
৪১০	আপনি কি আমাকে ঐ জাতীয় লুব্রিকেন্ট এর নাম বলতে পারবেন?	হ্যাঁ ১<br>নাম লিখুন ---------------------------<br>না ২<br>জানি না/মনে নাই ৯৭<br>উত্তর না দেয়া ৯৮		
৪১১	গত ১ মাসের মধ্যে কি হারে আপনি কনডমের জন্য তৈরী লুব্রিকেন্ট এবং কনডম **একসাথে** **পায়ূপথে** যৌনমিলনের সময় ব্যবহার করেছেন?	সব সময় ১<br>মাঝে মাঝে ২<br>কখনো না ৩<br>জানি না/মনে নাই ৯৭<br>উত্তর না দেয়া ৯৮<br>গত এক মাসে যৌনকাজ করে নাই ৯৯	→ ৪১৩<br>→ ৪১২<br>→ ৪১২<br>→ ৪১৪<br>→ ৪১৪<br>→ ৫০১	
৪১২	আপনি কেন **মাঝে মাঝে অথবা কখনোই** কনডমের জন্য বিশেষভাবে তৈরী লুব্রিকেন্ট এবং কনডম **একসাথে** ব্যবহার করেন না?<br><br>(পড়ে শোনাবেন না)<br>(একাধিক উত্তর সম্ভব)<br>(উলেণ্টখ করলে ১-এ গোল কর ্ন)<br>(উলেণ্টখ না করলে ২-এ গোল কর ্ন)	দাম বেশী ১ ২<br>কিনতে লজ্জা ১ ২<br>কোথায় পাওয়া যায় জানি না ১ ২<br>ব্যবহার করার দরকার নাই ১ ২<br>অন্য ক্রীম ব্যবহার করি ১ ২<br>সরবরাহ কম ১ ২<br>সহজে বহনযোগ্য নয় ১ ২<br>অন্যান্য ----------------------------- ১ ২<br>জানি না/মনে নাই ৯৭<br>উত্তর না দেয়া ৯৮	→ ৪১৪	
৪১৩	কি কারনে আপনি **সবসময়** যৌনমিলনের সময় কনডমের সাথে বিশেষভাবে তৈরী লুব্রিকেন্ট ব্যবহার করেন?<br><br>(পড়ে শোনাবেন না)<br>(একাধিক উত্তর সম্ভব)<br>(উলেণ্টখ করলে ১-এ গোল কর ্ন)<br>(উলেণ্টখ না করলে ২-এ গোল কর ্ন)	ফোলা/ব্যথা কমানো/সহজে প্রবেশ ১ ২<br>বেশী অনুভূতি ১ ২<br>কনডম ফাটার ঝুঁকি কমানো ১ ২<br>STI/HIV/AIDS কে বাধা দেয়া ১ ২<br>অন্যান্য ----------------------------- ১ ২<br>জানি না/মনে নাই ৯৭<br>উত্তর না দেয়া ৯৮		
৪১৪	গতমাসে যখন আপনি **পায়ূপথে** যৌনমিলনের সময় কনডম ব্যবহার করেছিলেন তখন কি আপনার কনডম ফেটেছিল?	হ্যাঁ ১<br>না ২<br>জানি না/মনে নাই ৯৭<br>গত মাসে কনডম ব্যবহার করি নাই ৩<br>জীবনে কনডম ব্যবহার করি নাই ৪<br>উত্তর না দেয়া ৯৮<br>গত এক মাসে যৌনকাজ করি নাই ৯৯	 <br><br>→ ৫০১<br>→ ৫০১<br>→ ৫০১<br>→ ৫০১	
৪১৫	গত ১ মাসে আপনি কোথায় অথবা কার নিকট থেকে কনডম পেয়েছেন?<br><br>**(গত মাসে যারা কনডম ব্যবহার করেছে শুধুমাত্র তাদেরকেই এই প্রশ্ন জিজ্ঞাসা কর ্ন)**<br><br>(পড়ে শোনাবেন না)<br>(একাধিক উত্তর গ্রহণযোগ্য)<br><br>(উলেণ্টখ করলে ১-এ গোল কর ্ন)<br>(উলেণ্টখ না করলে ২-এ গোল কর ্ন)	দোকান ১ ২<br>ফার্মেসী ১ ২<br>ডিআইসি ১ ২<br>ডিপো হোল্ডার ১ ২<br>স্বাস্থ্য সেবা কেন্দ্র (ডিআইসি ছাড়া) ১ ২<br>বার/গেষ্ট হাউস/হোটেল ১ ২<br>বন্ধুবান্ধব ১ ২<br>দালাল ১ ২<br>N.G.O কর্মী ১ ২<br>এক মাসের আগে কেনা ছিল ১ ২<br>যৌন সঙ্গী ১ ২<br>অন্যান্য ----------------------------- ১ ২<br>জানি না/মনে নাই ৯৭<br>উত্তর না দেয়া ৯৮		

সেকশন ৫ ঃ এখন আমি যৌন রোগ সম্পর্কে বলব

নং	প্রশ্ন	কোডের ধরন	নির্দেশ	মন্তব্য
৫০১	আপনি কি পুরুষের এমন কোন রোগের **লক্ষণ বা উপসর্গের** কথা জানেন যা যৌনমিলনের মাধ্যমে ছড়ায়?<br><br>(পড়ে শোনাবেন না)<br>(একাধিক উত্তর সম্ভব)<br>(উল্লেখ করলে ১-এ গোল করুন)<br>(উল্লেখ না করলে ২-এ গোল করুন)	পুরুষাঙ্গে পুঁজ/স্রাব ১ ২<br>প্রস্রাবে জ্বালাপোড়া ১ ২<br>পুরুষাঙ্গে ঘাঁ/ক্ষত ১ ২<br>কুচকিতে বাগী ১ ২<br>পায়ূপথে স্রাব ১ ২<br>পায়ূপথে ঘাঁ/ক্ষত ১ ২<br>অন্যান্য---------------------------- ১ ২<br>জানি না/মনে নাই ৯৭<br>উত্তর না দেয়া ৯৮		
৫০২	গত ১২ মাসের মধ্যে আপনার কি মূত্রনালী থেকে স্রাব/পুঁজ বেরিয়েছে? (কামরস ব্যতিত তরল কিংবা আঠালো জাতীয় কিছু)	হ্যাঁ ১<br>না ২<br>জানি না/মনে নাই ৯৭<br>উত্তর না দেয়া ৯৮		
৫০৩	গত ১২ মাসের মধ্যে আপনার কি পায়ূপথ থেকে স্রাব/পুঁজ (তরল কিংবা আঠালো জাতীয় কিছু) বেরিয়েছে?	হ্যাঁ ১<br>না ২<br>জানি না/মনে নাই ৯৭<br>উত্তর না দেয়া ৯৮		
৫০৪	গত ১২ মাসের মধ্যে আপনার কি পুরুষাঙ্গে ঘাঁ/ক্ষত হয়েছিল?	হ্যাঁ ১<br>না ২<br>জানি না/মনে নাই ৯৭<br>উত্তর না দেয়া ৯৮		
৫০৫	প্রশ্ন নং ৫০২, ৫০৩, ৫০৪ দেখুন যদি যে কোন একটিতে হ্যাঁ হয় তবে "**১**"গোল করুন অন্যথায় "**২**" গোল করুন।	হ্যাঁ ১<br>না ২	→ ৫০৯	
৫০৬ক	শেষবার আপনার যখন যৌন রোগের লক্ষণ দেখা দিয়েছিল তখন প্রথমে কি করেছিলেন?<br><br>(পড়ে শোনাবেন না)<br>(একটি উত্তর হবে)	সরকারী হাসপাতাল থেকে চিকিৎসা ০<br>বেসরকারী হাসপাতাল থেকে চিকিৎসা ১<br>ঔষধ বিক্রেতা থেকে চিকিৎসা ২<br>প্রাইভেট ডাক্তার দ্বারা চিকিৎসা ৩<br>প্রাইভেট ক্লিনিক থেকে চিকিৎসা ৪<br>এন জি ও ক্লিনিক থেকে চিকিৎসা ৫ →<br>সনাতনী চিকিৎসা ৬<br>বন্ধুবান্ধব থেকে উপদেশ/চিকিৎসা ৭<br>নিজে নিজে চিকিৎসা ৮<br>কিছুই না ৯ →<br>অন্যান্য---------------------------- ১০<br>জানি না/মনে নাই ৯৭<br>উত্তর না দেয়া ৯৮	৫০৬খ জিজ্ঞাসা করুন<br><br>৫০৯	
৫০৬খ	এনজিও ক্লিনিকের নাম			
৫০৭	শেষবার (গত ১২ মাসে) যৌনরোগের লক্ষণ দেখা দেয়ার কতদিন পর চিকিৎসা নিয়েছিলেন?<br><br>(যদি প্রথম দিনই চিকিৎসা নেয় তাহলে ১ দিন লিখুন)	দিন ---<br>এক দিনের মধ্যে ০<br>জানি না/মনে নাই ৯৭<br>উত্তর না দেয়া ৯৮		
৫০৮	শেষবার (গত ১২ মাসে) যখন চিকিৎসা নিয়েছিলেন তখন মোট কত টাকা খরচ হয়েছিল? **(ঔষধ এবং ডাক্তারের ফি সহ)**	টাকা ----<br>জানি না/মনে নাই ৯৭<br>উত্তর না দেয়া ৯৮		
৫০৯	যৌনমিলনের মাধ্যমে যেসব রোগ ছড়ায় সেই রোগ যাতে আপনার না হয় সে জন্য আপনি কি করেন?<br><br>(পড়ে শোনাবেন না)<br>(একাধিক উত্তর সম্ভব)<br>(উল্লেখ করলে ১-এ গোল করুন)<br>(উল্লেখ না করলে ২-এ গোল করুন)	কিছুই না ১ ২ →<br>সাবান/যৌনমিলনের পর ডেটল/প্রস্রাব দ্বারা যৌনাঙ্গ ধোয়া ১ ২<br>সর্বদা কনডম ব্যবহার করা ১ ২<br>মাঝে মাঝে কনডম ব্যবহার ১ ২<br>সবসময় একজন/বিশ্বস্ত সঙ্গীর সাথে যৌন কাজ ১ ২<br>মহিলা যৌন কর্মী পরিহারের চেষ্টা ১ ২<br>যৌন কাজের আগে সঙ্গী পরীক্ষা/পরিষ্কার সঙ্গীর সাথে সেক্স করা ১ ২	৫১১<br>(যদি ১ হয়)	

নং	প্রশ্ন	কোডের ধরন	নির্দেশ	মন্তব্য
		অন্যান্য---------------------------- ১ ২<br>ঔষধ গ্রহন ১ ২<br>জানি না/মনে নাই ৯৭<br>উত্তর না দেয়া ৯৮	→ ৫১০ জিজ্ঞাসা করুন	
৫১০	আপনি কি ঔষধ গ্রহন করেন?<br>**(৫০৯ প্রশ্নে ঔষধ গ্রহণ করে থাকলে ঔষধের নাম অবশ্যই আনতে হবে)**	ঔষধের নাম -----------------------<br>জানি না/মনে নাই ৯৭<br>উত্তর না দেয়া ৯৮		
৫১১	গত এক মাসে এই শহরে যৌনরোগের (STI) সেবা দেয় একরম কোন NGO ক্লিনিকে গিয়েছিলেন কি?	হ্যাঁ ১<br>না ২<br>জানি না/মনে নাই ৯৭<br>উত্তর না দেয়া ৯৮	(না/জানি না/উত্তর না দেয়া) → ৬০১	
৫১২	যদি হ্যাঁ হয়, কোন ক্লিনিকে গিয়েছিলেন?<br>(একাধিক উত্তর গ্রহনযোগ্য)	NGO/ক্লিনিকের নামঃ<br>১. ____________<br>২. ____________<br>৩. ____________		

সেকশন ৬ ঃ এখন আমি AIDS সম্মন্ধে জ্ঞান, ঝুঁকি এবং প্রতিরোধ সম্পর্কে কথা বলব

নং	প্রশ্ন	কোডের ধরন	নির্দেশ	মন্তব্য
৬০১	আপনি কি আগে কখনো HIV অথবা AIDS নামক রোগের নাম শুনেছেন?	হ্যাঁ ১<br>না ২<br>উত্তর না দেয়া ৯৮	(না) → ৭০১	
৬০২	প্রতিবার যৌনমিলনের সময় সঠিকভাবে কনডম ব্যবহারের মাধ্যমে মানুষ কি HIV - এর ঝুঁকি কমাতে পারে?	হ্যাঁ ১<br>না ২<br>জানি না/মনে নাই ৯৭<br>উত্তর না দেয়া ৯৮		
৬০৩	**পায়ুপথে** যৌনমিলন না করে মানুষ কি HIV - এর ঝুঁকি কমাতে পারে?	হ্যাঁ ১<br>না ২<br>জানি না/মনে নাই ৯৭<br>উত্তর না দেয়া ৯৮		
৬০৪	**যোনী/পায়ুপথে** যৌনমিলনের সময় প্রতিবার সঠিকভাবে কনডম ব্যবহারের মাধ্যমে মানুষ কি HIV - এর ঝুঁকি কমাতে পারে?	হ্যাঁ ১<br>না ২<br>জানি না/মনে নাই ৯৭<br>উত্তর না দেয়া ৯৮		
৬০৫	একাধিক যৌনসঙ্গী পরিহার করে কি কেউ HIV -এর ঝুঁকি কমাতে পারে?	হ্যাঁ ১<br>না ২<br>জানি না/মনে নাই ৯৭<br>উত্তর না দেয়া ৯৮		
৬০৬	মশার কামড় থেকে কি কোন মানুষ HIV দ্বারা আক্রান্ড় হতে পারে?	হ্যাঁ ১<br>না ২<br>জানি না/মনে নাই ৯৭<br>উত্তর না দেয়া ৯৮		
৬০৭	HIV দ্বারা আক্রান্ড় লোকের সাথে একত্রে খাবার খেয়ে কেউ কি HIV দ্বারা আক্রান্ড় হতে পারে?	হ্যাঁ ১<br>না ২<br>জানি না/মনে নাই ৯৭<br>উত্তর না দেয়া ৯৮		
৬০৮	অন্যের ব্যবহৃত সূঁচ/সিরিঞ্জ ব্যবহার করলে কেউ কি HIV দ্বারা আক্রান্ড় হতে পারে?	হ্যাঁ ১<br>না ২<br>জানি না/মনে নাই ৯৭<br>উত্তর না দেয়া ৯৮		

নং	প্রশ্ন	কোডের ধরন	নির্দেশ	মন্তব্য
৬০৯	আপনি কি মনে করেন যে কাউকে দেখেই বলা যাবে সে HIV দ্বারা আক্রান্ত?	হ্যাঁ ১<br>না ২<br>জানি না/মনে নাই ৯৭<br>উত্তর না দেয়া ৯৮		
৬১০	আপনার নিজের যাতে HIV না হয় তার জন্য আপনি কি করেন?<br><br>(পড়ে শোনাবেন না)<br>(একাধিক উত্তর গ্রহনযোগ্য)<br>(উল্লেখ করলে ১-এ গোল করুন)<br>(উল্লেখ না করলে ২-এ গোল করুন)	কিছুই না ১ ২<br>যৌনমিলনের পর ডেটল/প্রস্রাব দ্বারা যৌনাঙ্গ ধোয়া ১ ২<br>সর্বদা কনডম ব্যবহার করা ১ ২<br>মাঝে মাঝে কনডম ব্যবহার ১ ২<br>ঔষধ গ্রহন ১ ২<br>সবসময় একজন/বিশ্বস্ত সঙ্গীর সাথে যৌন কাজ ১ ২<br>যৌন কাজের আগে সঙ্গী পরীক্ষা/পরিষ্কার ১ ২<br>সঙ্গীর সাথে সেক্স করা ১ ২<br>অন্যান্য---------------------------- ৯৭<br>জানি না/মনে নাই ৯৮<br>উত্তর না দেয়া	→ ৬১১ (যদি ১ হয়)	

গোপনীয় ভাবে HIV এর পরীক্ষা
(গোপনীয় বলতে বুঝায় যে আপনি নিজে ব্যতীত অন্য কেউ এর ফলাফল জানবে না)

নং	প্রশ্ন	কোডের ধরন	নির্দেশ	মন্তব্য
৬১১	আপনি HIV দ্বারা আক্রান্ত কিনা তা যদি পরীক্ষা করে দেখতে চান তাহলে গোপনীয়ভাবে কোথায় করতে পারবেন তা কি জানেন?	হ্যাঁ ১<br>না ২<br>উত্তর না দেয়া ৯৮	(না ২) → ৭০১	
৬১২	(পরীক্ষার ফলাফল আমি জানতে চাইনা)<br>আপনি কি কখনো HIV পরীক্ষা করেছেন?	হ্যাঁ ১<br>না ২<br>জানি না/মনে নাই ৯৭<br>উত্তর না দেয়া ৯৮	(না ২) → ৭০১	
৬১৩	হ্যাঁ হলে সর্বশেষ কোথায় HIV পরীক্ষা করেছেন?			
৬১৪	আপনি কি স্বেচ্ছায় HIV পরীক্ষা করিয়েছেন নাকি অন্য কেউ উৎসাহিত করেছে অথবা আপনাকে পরীক্ষা করতে হয়েছে?	স্বেচ্ছায় ১ → ৬১৭ জিজ্ঞাসা করুন<br>অন্য কেউ উৎসাহিত করেছিল ২ → ৬১৫ জিজ্ঞাসা করুন<br>প্রয়োজন হয়েছিল ৩ → ৬১৬ জিজ্ঞাসা করুন<br>উত্তর না দেয়া ৯৮ → ৬১৭ জিজ্ঞাসা করুন		
৬১৫	কে উৎসাহিত করেছিল? **(একটি মাত্র উত্তর হবে)**			
৬১৬	কেন প্রয়োজন হয়েছিল? **(একটি মাত্র উত্তর হবে)**			
৬১৭	আমি ফলাফল জানতে চাই না, আপনি ঐ ফলাফল পেয়েছিলেন কি?	হ্যাঁ ১<br>না ২<br>জানি না/মনে নাই ৯৭<br>উত্তর না দেয়া ৯৮		
৬১৮	সর্বশেষ কতদিন আগে আপনি HIV পরীক্ষা করিয়েছেন?	গত ৬ মাসের মধ্যে ০<br>৬ মাস থেকে ১ বছরের মধ্যে ১<br>১ বছর আগে ২<br>জানি না/মনে নাই ৯৭<br>উত্তর না দেয়া ৯৮		

সেকশন ৭ ঃ নির্যাতন সংক্রান্ড় তথ্য

নং	প্রশ্ন	কোডের ধরন	নির্দেশ	মন্ড়ব্য
৭০১	গত ১২ মাসে কেউ কি আপনাকে মারধোর করেছে?	হ্যাঁ ১<br>না ২<br>জানি না/মনে নাই ৯৭<br>উত্তর না দেয়া ৯৮	(না/জানি না/উত্তর না দেয়া) → ৭০৩	
৭০২	(যদি কেউ মারধর করে থাকে তাহলে) কে আপনাকে মারধর করেছে?<br><br>(পড়ে শোনাবেন না)<br>(একাধিক উত্তর সম্ভব)<br>(উলেণ্টখ করলে ১-এ গোল করুন)<br>(উলেণ্টখ না করলে ২-এ গোল করুন)	আইন শৃংখলা রক্ষাকারী বাহিনী ১ ২<br>মাস্ড়ান ১ ২<br>নতুন যৌন সঙ্গী ১ ২<br>নিয়মিত যৌন সঙ্গী ১ ২<br>মহলণ্টার লোক ১ ২<br>পরিবার/আত্নীয় ১ ২<br>অন্যান্য-------------------------- ১ ২<br>জানি না/মনে নাই ৯৭<br>উত্তর না দেয়া ৯৮		
৭০৩	গত ১২ মাসে কেউ কি আপনার ইচ্ছার বির"দ্ধে আপনার সাথে যৌনমিলন করেছে?	হ্যাঁ ১<br>না ২<br>জানি না/মনে নাই ৯৭<br>উত্তর না দেয়া ৯৮	(না/জানি না/উত্তর না দেয়া) → ৭০৫	
৭০৪	গত ১২ মাসে কে বা কারা আপনার ইচছার বিরূদ্ধে জোরপূর্বক যৌনমিলন করেছে?<br><br>(পড়ে শোনাবেন না)<br>(একাধিক উত্তর সম্ভব)<br>(উলেণ্টখ করলে ১-এ গোল করুন)<br>(উলেণ্টখ না করলে ২-এ গোল করুন)	আইন শৃংখলা রক্ষাকারী বাহিনী ১ ২<br>মাস্ড়ান ১ ২<br>নতুন যৌন সঙ্গী ১ ২<br>নিয়মিত যৌন সঙ্গী ১ ২<br>মহলণ্টার লোক ১ ২<br>আত্নীয় ১ ২<br>অন্যান্য-------------------------- ১ ২<br>জানি না/মনে নাই ৯৭<br>উত্তর না দেয়া ৯৮		
৭০৫	গত ১২ মাসের মধ্যে আপনি কি গ্রেপ্তার হয়েছিলেন?	হ্যাঁ ১<br>না ২<br>জানি না/মনে নাই ৯৭<br>উত্তর না দেয়া ৯৮	(না/জানি না/উত্তর না দেয়া) → ৮০১	
৭০৬	**যদি হ্যাঁ হয়**, কি কারনে গ্রেফতার হয়েছিলেন ?	১. . . . .<br>২. . . . .<br>৩.		

সেকশন ৮ ঃ ঝুঁকি উপলব্ধি

নং	প্রশ্ন	কোডের ধরন		নির্দেশ	মন্তব্য
৮০১	আপনি কি মনে করেন আপনার HIV -এর ঝুঁকি বেশী, মাঝারী, কম বা ঝুঁকি নেই?	বেশী	১	→ ৮০২	
		মাঝারী	২	→ ৮০২	
		ঝুঁকি কম	৩	→ ৮০৩	
		ঝুঁকি নেই	৪	→ ৮০৩	
		জানি না/মনে নাই	৯৭	→ ৯০১	
		উত্তর না দেয়া	৯৮	→ ৯০১	
৮০২	আপনি কেন মনে করেন আপনার HIV হওয়ার **বেশী বা মাঝারী ঝুঁকি** রয়েছে?<br><br>(পড়ে শোনাবেন না)<br>(একাধিক উত্তর সম্ভব)<br><br>(উলেণ্ঢখ করলে ১-এ গোল কর্ন)<br>(উলেণ্ঢখ না করলে ২-এ গোল কর্ন)	ঝুঁকিপূর্ন কাজ	১ ২	এই প্রশ্নের উত্তর আসলে ৮০৩ প্রশ্ন করার দরকার নাই।	
		পুনঃপুনঃ পায়ূপথে যৌনকাজ	১ ২		
		কনডম ব্যবহার না করা	১ ২		
		অনিয়মিত কনডম ব্যবহার	১ ২		
		সূঁচ/সিরিঞ্জ ভাগাভাগি	১ ২		
		অন্যান্য ---------------------------------	১ ২		
		জানি না/মনে নাই	৯৭		
		উত্তর না দেয়া	৯৮		
৮০৩	আপনি কেন মনে করেন আপনার HIV হওয়ার **ঝুঁকি নাই অথবা কম?**<br>(পড়ে শোনাবেন না)<br>(একাধিক উত্তর সম্ভব)<br>(উলেণ্ঢখ করলে ১-এ গোল কর্ন)<br>(উলেণ্ঢখ না করলে ২-এ গোল কর্ন)	সর্বদা কনডম ব্যবহার	১ ২		
		পরিস্কার সঙ্গী/ খদ্দের	১ ২		
		স্বাস্থ্যবান সঙ্গী/ খদ্দের	১ ২		
		কখনও সূঁচ/সিরিঞ্জ ভাগাভাগী করি না	১ ২		
		মাঝে মাঝে সূঁচ/সিরিঞ্জ ভাগাভাগী করি	১ ২		
		অনিয়মিত কনডম ব্যবহার	১ ২		
		সবসময় একজন/বিশ্বস্ড় সঙ্গীর সাথে যৌন কাজ	১ ২		
		সবসময় পরিস্কার পরিচ্ছন্ন থাকি	১ ২		
		কম যৌনমিলন করি	১ ২		
		যৌনমিলন করার পর ধুয়ে ফেলি	১ ২		
		অন্যান্য ---------------------------------	১ ২		
		জানি না/মনে নাই	৯৭		
		উত্তর না দেয়া	৯৮		

সেকশন ৯ ঃ কর্মসূচীতে অংশগ্রহণ

নং	প্রশ্ন	কোডের ধরন		নির্দেশ	মন্তব্য
৯০১	আপনি কি জীবনে কখনো **এন জি ও/সেল্ফ হেল্প গ্র্প/সিবিও** পরিচালিত কোন এইডস প্রতিরোধ কর্মসূচীতে অংশগ্রহণ করেছিলেন?	হ্যাঁ	১		
		না	২	→ 907	
		এই এলাকায় কোন এন জি ও/সেল্ফ হেল্প গ্র্প/সিবিও নাই	৯৬		
		জানি না/মনে নাই	৯৭		
		উত্তর না দেয়া	৯৮		
৯০২	এইডস প্রতিরোধ কর্মসূচীতে আপনি কত দিন যাবৎ যুক্ত আছেন?	. . . . মাস			
		১ মাসের মধ্যে	০০		
		জানি না/মনে নাই	৯৭		
		উত্তর না দেয়া	৯৮		
৯০৩	আপনি সর্বশেষ কত মাস আগে এইডস প্রতিরোধ কর্মসূচীতে অংশগ্রহণ করেছিলেন?	. . . . মাস			
		১ মাসের মধ্যে	০০		
		জানি না/মনে নাই	৯৭		
		উত্তর না দেয়া	৯৮		
৯০৪	গত মাসে কতবার এইডস প্রতিরোধ কর্মসূচীতে অংশ নিয়েছেন?	শূন্য	০		
		সংখ্যা	-----		
		জানি না/মনে নাই	৯৭		
		উত্তর না দেয়া	৯৮		
৯০৫ক	আপনি **গত মাসে** কি কি ধরনের কর্মসূচীতে অংশগ্রহণ করেছিলেন?<br>(একাধিক উত্তর গ্রহন যোগ্য)<br>(পড়ে শোনাবেন না)<br>উলেণ্ঢখ করলে ১ গোল কর্ন	স[illegible]চ/সিরিঞ্জ বিনিময় কর্মসৃ[illegible]চী	১ ২		
		শিক্ষাম[illegible]লক কার্যক্রম	১ ২		
		কনডম পেয়েছেন	১ ২		
		লুব্রিকেন্ট পেয়েছেন	১ ২		
		যৌনরোগের জন্য চিকিৎসা পেয়েছেন	১ ২		

নং	প্রশ্ন	কোডের ধরন	নির্দেশ	মন্তব্য
	উলেণ্ঢখ না করলে ২ গোল করুন	সাধারন স্বাস্থ্য সেবা পেয়েছি ১ ২<br>IHC তে অংশগ্রহণ ১ ২<br>DIC তে অংশগ্রহণ ১ ২<br>VCT তে অংশগ্রহণ ১ ২<br>অন্যান্য---------------------------------- ১ ২<br>জানি না/মনে নাই ৯৭<br>উত্তর না দেয়া ৯৮		
৯০৫খ	আপনি **<u>সারা জীবনে</u>** কি কি ধরনের কর্মসূচীতে অংশগ্রহণ করেছিলেন?<br>(একাধিক উত্তর গ্রহন যোগ্য)<br>(পড়ে শোনাবেন না)<br>উলেণ্ঢখ করলে ১ গোল করুন<br>উলেণ্ঢখ না করলে ২ গোল করুন	সুঁচ/সিরিঞ্জ বিনিময় কর্মসূচী ১ ২<br>শিক্ষামূলক কার্যক্রম ১ ২<br>কনডম পেয়েছেন ১ ২<br>লুব্রিকেন্ট পেয়েছেন ১ ২<br>যৌনরোগের জন্য চিকিৎসা পেয়েছেন ১ ২<br>সাধারন স্বাস্থ্য সেবা পেয়েছি ১ ২<br>IHC তে অংশগ্রহণ ১ ২<br>DIC তে অংশগ্রহণ ১ ২<br>VCT তে অংশগ্রহণ ১ ২<br>অন্যান্য---------------------------------- ১ ২<br>জানি না/মনে নাই ৯৭<br>উত্তর না দেয়া ৯৮		
৯০৬	আপনি এ কর্মসূচি থেকে (**<u>সারাজীবনে</u>**) কিভাবে উপকৃত হয়েছিলেন?<br><br>(পড়ে শোনাবেন না)<br>(একাধিক উত্তর সম্ভব)<br>(উলেণ্ঢখ করলে ১-এ গোল করুন)<br>(উলেণ্ঢখ না করলে ২-এ গোল করুন)	আচরন পরিবর্তন করতে সাহায্য করেছিল ১ ২<br>মূল্যবান তথ্য ছিল কিন্তু আচরন পরিবর্তন হয়নি ১ ২<br>এইচআইভি/এইডস/এসটিডি/নিরাপদ সেক্স ও কনডমের সঠিক ব্যবহার সম্পর্কে জানতে পেরেছি ১ ২<br>তথ্য সহজবোধ্য ছিল না ১ ২<br>আমাদের প্রয়োজনের সাথে মিল ছিল না ১ ২<br>অন্যান্য---------------------------------- ১ ২		

সেকশন ১০ ঃ সাক্ষাতের স্থান এবং বিবিধ

নং	প্রশ্ন	কোডের ধরন	নির্দেশ	মন্তব্য
৯০৭	আপনি আপনার জনগোষ্ঠির বন্ধুদের সাথে কোথায় দেখা করেন?<br><br>(পড়ে শোনাবেন না)<br>(একাধিক উত্তর সম্ভব)<br>(উলেণ্ঢখ করলে ১-এ গোল করুন)<br>(উলেণ্ঢখ না করলে ২-এ গোল করুন)	ক্রুজিং স্পটে ১ ২<br>বাসায় ১ ২<br>ক্লাবে ১ ২<br>পার্টিতে ১ ২<br>চায়ের দোকান ১ ২<br>রাস্তায় ১ ২<br>বাজার/মার্কেট ১ ২<br>হোটেল/বোর্ডিং ১ ২<br>কাজের জায়গায় ১ ২<br>স্কুল/কলেজ/মাদ্রাসা ১ ২<br>অন্যান্য.. ১ ২<br>জানি না/মনে নাই ৯৭<br>উত্তর না দেয়া ৯৮		

নং	প্রশ্ন	কোডের ধরন	নির্দেশ	মন্তব্য
৯০৮	আপনি কিভাবে পুরুষ সঙ্গীর সাথে যৌন কাজের জন্য যোগাযোগ করেন?<br><br>(পড়ে শোনাবেন না)<br>(একাধিক উত্তর সম্ভব)<br>(উল্লেখ করলে ১-এ গোল করুন)<br>(উল্লেখ না করলে ২-এ গোল করুন)	ক্রুজিং স্পটে ১ ২<br>ফোনের মাধ্যমে ১ ২<br>ইন্টারনেটের মাধ্যমে ১ ২<br>বন্ধু-বান্ধবের মাধ্যমে ১ ২<br>দালালের মাধ্যমে ১ ২<br>ক্লাবে ১ ২<br>পার্টিতে ১ ২<br>চায়ের দোকান ১ ২<br>রাস্তায় ১ ২<br>বাজার/মার্কেট ১ ২<br>হোটেল/বোর্ডিং ১ ২<br>কাজের জায়গায় ১ ২<br>স্কুল/কলেজ/মাদ্রাসা ১ ২<br>বাসায় ১ ২<br>অন্যান্য.. ১ ২<br>জানি না/মনে নাই ৯৭<br>উত্তর না দেয়া ৯৮		
৯০৯	যৌন আচরনের উপর ভিত্তি করে আপনি আপনাকে কিভাবে পরিচয় দিতে পছন্দ করেন?<br><br>(পড়ে শোনাবেন না)<br>(একটি উত্তর হবে)	পুরুষ/ম্যানলি/সাধারন মানুষ ১<br>পারিখ ২<br>সিনেমার নায়ক ৩<br>পান্থি ৪<br><br>সিনেমার নায়িকা ৫<br>গে ৬<br>কতি ৭<br>দো-পারাটা ৮<br>মেয়ে/নারী ৯<br>অন্যান্য.. ১০<br>জানি না/মনে নাই ৯৭<br>উত্তর না দেয়া ৯৮	(১–৪) → ধন্যবাদ দিয়ে সাক্ষাৎকার সমাপ্ত করুন।<br><br>(৫–৯) → ৯১০	
৯১০	আপনি কি কখনও মেয়েলি ভাব আনার জন্য কোন প্রকার ঔষধ সেবন করেছেন?	হ্যাঁ ১<br>না ২<br>জানি না/মনে নাই ৯৭<br>উত্তর না দেয়া ৯৮	(২, ৯৭, ৯৮) → ধন্যবাদ দিয়ে সাক্ষাৎকার সমাপ্ত করুন।	
৯১১	যদি নিয়ে থাকেন, গত তিন মাসে নিয়েছেন কি?	হ্যাঁ ১<br>না ২<br>জানি না/মনে নাই ৯৭<br>উত্তর না দেয়া ৯৮	(২, ৯৭, ৯৮) → ধন্যবাদ দিয়ে সাক্ষাৎকার সমাপ্ত করুন।	
৯১২	গত তিন মাসে কি কি ঔষধ সেবন করেছেন?<br><br>(পড়ে শোনাবেন না)<br>(একাধিক উত্তর সম্ভব)<br>(উল্লেখ করলে ১-এ গোল করুন)<br>(উল্লেখ না করলে ২-এ গোল করুন)	সুখী (Shukhi) ১ ২<br>সিলেস্ট-২১ (Cilest-21) ১ ২<br>ডেসোলন (Desolon) ১ ২<br>লাইনেস (Lynes) ১ ২<br>মারভেলন (Marvelon) ১ ২<br>ওভস্টেট (Ovostat) ১ ২<br>ফেমিকন (Femecon) ১ ২<br>নরডেট-২৮ (Nordat-28) ১ ২<br>এমকন (Emcon) ১ ২<br>পোসটিনর-২ (Postinor-2) ১ ২<br>মিনিকন (Minicon) ১ ২<br>ওভাকন (Ovacon) ১ ২<br>অন্যান্য.. ১ ২<br>জানি না/মনে নাই ৯৭<br>উত্তর না দেয়া ৯৮		

নং	প্রশ্ন	কোডের ধরন	নির্দেশ	মন্তব্য
৯১৩	গত তিন মাসে ঐ সকল ঔষধ সেবনের কারণগুলো কি?<br><br>(পড়ে শোনাবেন না)<br>(একাধিক উত্তর সম্ভব)<br>(উল্লেখ করলে ১-এ গোল করুন)<br>(উল্লেখ না করলে ২-এ গোল করুন)	বুকের মাপ বৃদ্ধি করার জন্য ১ ২<br>উরু/পাছা বৃদ্ধি/মসৃন করার জন্য ১ ২<br>শরীরের কোমলতা বৃদ্ধি করার জন্য ১ ২<br>দাড়ি/গোঁফ না গজানোর জন্য ১ ২<br>অন্যান্য.. ১ ২<br>জানি না/মনে নাই ৯৭<br>উত্তর না দেয়া ৯৮		
৯১৪	আপনি গত তিন মাসে কি হারে ঐ সকল ঔষধ সেবন করেছেন?<br><br>(পড়ে শোনাবেন না)	প্রতিদিন ১<br>সপ্তাহে একাধিক বার ২<br>সপ্তাহে একবার ৩<br>মাসে একবার ৪<br>মাসে একাধিক বার ৫<br>জানি না/মনে নাই ৯৭<br>উত্তর না দেয়া ৯৮		

আপনার মূল্যবান সময় দিয়ে সহযোগীতা করার জন্য অসংখ্য ধন্যবাদ।

=<=
