# Supplementary material for: Changes in condom use among males who have sex with males (MSM): Measuring the effect of HIV prevention programme in Dhaka city
Source: PLoS One. 2020 Jul 24;15(7):e0236557. doi: 10.1371/journal.pone.0236557 (PMC7380615; doi:10.1371/journal.pone.0236557)
Supplement: S1 File — (ZIP) [file pone.0236557.s001.zip › MSM_ BSS Midline English Questionnaire_2013.pdf]

Annex-3

Questionnaire identification number [ ]

MIDLINE HIV RISK BEHAVIOURAL SURVEY FORM 2013
MSM

I (name) am working in a research project of icddr,b (Cholera hospital). We are trying to find out how to help people avoid a sickness called AIDS. We need to ask you some very personal questions. Nothing you tell me will be used for anything but the purposes of this survey. Your name or address will never be written down. You can refuse without any problem and you can stop the interview at any time after we start. What you tell me will be kept strictly confidential. Because we sincerely want to help all the people of Bangladesh avoid AIDS, if you agree to give the interview, it is really important that you are willing to be very truthful. Is it all right to begin?

Yes No

If no, why (only one answer allowed): ……………………………………………….

Consent of the Guardian /Senior Community Fellow (applicable for those who are 15 to <18 years old)

Do you agree to allow this MSM/MSW/hijra to participate in this study, if 'Yes' then please indicate that by putting your signature or your left thumb impression at the specified space below

____ ______________________

Signature or left thumb impression of Guardian or Senior Community Fellow — Date

Name of the location: -

Cluster code: [ ]

Thana code: [ ]

Interviewer code: [ ]

Date of Interview: -- (DD/MM/YYYY)

Interview starting time: ----------------------------- (HH:MM) (24HR)

Interview ending time: ------------------------------ (HH:MM) (24HR)

Has the interview been completed?

Yes [ ]

No [ ]

If no, why (only one answer allowed): ……………………………………………….

Checked by the supervisor: Signature - - - - - - - - - - - - - - - - - - **Date: ------------------------**
(DD/MM/YYYY)

Section 1: Introduction

No.	Questions and filters	Coding categories	Skip to	Comments
101	How old are you?<br>(In completed years)	Years____		
102	How many years of education have you completed up to now?	# Years Completed _____<br>Less than one year 00<br>No response 98<br>Never been to school 99		
103	How long have you been living in this city?	Number of Years ______<br>less than 1 year 00<br>All my life 96<br>Don't know/Cannot remember 97<br>No response 98		
104	What is your total income from the last month?	Tk. _______.<br>Don't know/Cannot remember 97<br>No response 98		
105	How did you earn that money?<br><br>Multiple answers possible<br>Provide ranking	Ranking<br>Business 1 ----------<br>Service 2 -----<br>Driving 3 -----<br>Tuition/Teaching 4 ----------<br>Family 5<br>Others 6		
106	Have you sold blood for money in the last 12 months?	Yes....1<br>No....2<br>Don't know/Cannot remember ...97<br>No Response...98		
107	Some people take illicit drugs for fun or to get high. Have you taken any illicit drugs other than sleeping pill in the last 12 months?	Yes 1<br>No 2<br>Don't know/Cannot remember 97<br>No response 98	→ 109	
108	If yes, mention name of illicit drugs?<br><br>(Do not read out)<br>(Multiple answer possible)<br>(If yes, circle 1)<br>(If no, circle 2)	Alcohol 1 2<br>Cannabis 1 2<br>Phensidyl 1 2<br>Heroin 1 2<br>Injection (Buprenorphine/Pethedine)----------<br>------------------------ 1 2<br>Yaba 1 2<br>Others -------------------- 1 2		
109	When was the last time you took illicit drugs within 12 months period?	----------------------------------days before		
110	Within last 6 months period, which illicit drug did you take highest?<br>(single answer only)	Did not take any illicit drug within last 6 month 0<br><br>Alcohol 1<br>Cannabis 2		

No.	Questions and filters	Coding categories	Skip to	Comments
		Phensidyl 3<br>Heroin 4<br>Injection (Buprenorphine/Pethedine)5<br>Yaba 6<br>Others ------------------- 7		
111	Some people have tried injecting drugs for fun or to get high. Have you injected drugs in the last 12 months?	Yes 1<br>No 2<br>Don't know/Cannot remember .. 97<br>No Response 98	(No, Don't know, No Response) → 201	
112	Some people have tried injecting drugs for fun or to get high. Have you injected drugs in the last 2 months?	Yes….1<br>No….2<br>Don't know/Cannot remember …97<br>No Response…98		
113	Last time you injected, did you use a needle or syringe after someone else had used it or did you pass your needle or syringe on to someone else in the last 12 months?	Yes 1<br>No 2<br>Don't Know/ Don't Remember 97<br>No Response 98		

Section 2: Marriage and Partnership and Sexual History

No.	Questions and filters	Coding categories	Skip to	Comments
201	What is your current marital status?<br><br>**(**READ OUT**)**<br>(Only one response)	Married 1<br>Unmarried 2<br>Divorced 3<br>Widower 4<br>Separated 5<br>No Response 98	(Unmarried–Separated) →Q203	
202	Are you currently living with spouse?	Yes 1<br>No 2<br>No Response 98		
203	Do you currently have any regular sex partner (except wife)?	Yes .1<br>No. 2<br>No Response 98	(No) →205	
204	Who is your current sex partner?<br><br>(Multiple answers possible)	Male 1 2<br>Female 1 2<br>Hijra 1 2<br>No response 98		
205	At what age did you first have sexual intercourse?<br>(Anal and or vaginal sex)	Age In Years _____<br>Never had vaginal or anal sex 96<br>Don't know/Cannot remember 97<br>No Response 98	(Never had vaginal or anal sex) →331	
206A	Was your first sexual partner male, female or *hijra*?<br>(Only one response)	Male 1<br>Female 2<br>Hijra 3<br>Don't know/Cannot remember 97<br>No response 98		

No.	Questions and filters	Coding categories	Skip to	Comments
206B	How long have you been practicing male to male sex? (in years)	------------------- years<br>0 (less than 1 year)		
207A	Have you ever had anal sex with a male/*hijra* in exchange of money (sold sex)/by force gift?	Yes 1<br><br>No 2<br>No response 98	→ Ask 207B, then stop the interview with thanks<br>→207c	
207B	When did you have last anal sex with male/*hijra* sex partners in exchange of money (sold sex)/by force gift?	------ months<br>Within 1 month 0<br>Don't know/Cannot remember 97<br>No response 98		
207C	When did you have last anal sex with male (not *hijra*) sex partners in exchange of money (buying sex)/without money?	------ months<br>Within 1 month 0<br>Don't know/Cannot remember 97<br>No response 98		
208	When did you have last anal sex with *hijra* sex partners in exchange of money (buying sex)/without money?	------ months<br>Within 1 month 0<br>No sex with *hijra* 96<br>Don't know/Cannot remember 97<br>No response 98		
209	When did you have last vaginal/anal sex with female (not *hijra*) sex partners in exchange of money (buying sex)/without money?	------ months<br>Within 1 month 0<br>No sex with female 96<br>Don't know/Cannot remember 97<br>No response 98		
210	Did you ever use condom during any **receptive/penetrative** anal sex act?	Yes (receptive sex).......1<br>Yes (penetrative sex)......2<br>Yes (receptive and penetrative sex)<br>......3<br>Never use condom....4<br>No responses.....98		
211	Did you use condom during your last receptive anal sex act in the **last 12 months**?	Yes....1<br>No....2<br>Never have receptive sex ....3<br>Never use condom....4<br>No response...98		
212	Did you use condom during your last penetrative anal sex act in the **last 12 months**?	Yes....1<br>No....2<br>Never have penetrative sex ....3<br>Never use condom....4<br>No response...98		
213	Did you use condom in anal sex during your last receptive/penetrative sex act?	Yes....1<br>No....2<br>No response...98		

Section 3A: Sexual Behaviour with Male Partners

Let's talk about **non-paying male/Hijra sex partner**

No.	Questions and filters	Coding categories	Skip to	Comments
298	In the last **six-months**, have you had **anal** sex with a man/hijra where no payment was involved?	Yes…….1<br>No……2	 →306	
299	The last time (**in the last six months**) you had anal sex with a non-paying male/hijra partner, did you use a condom?	Yes……1<br>No…..2<br>Don't know/Cannot remember 97<br>No response....98		
300	In the last **one-month**, have you had sex **(anal or oral)** with a man/hijra where no payment was involved?	Yes…….1<br>No……2	 →306	
301	In the last **one month**, how many different male/ hijra partners have you had sex **(anal or oral)** with where no payment was involved?	Number______<br>Don't know/Cannot remember 97<br>No response.98		
302	In the last **one month**, how many times have you had **anal sex act** with your non-paying male/hijra sex partners?	Zero 0<br>Number ………….<br>Don't know/Cannot remember 97<br>No response…….98	→305	
303	The last time **(in the last one month)** you had **anal sex** with a non-paying male/hijra partner, did you use a condom?	Yes……1<br>No…..2<br>Don't know/Cannot remember 97<br>No response....98		
304	Of all times you had **anal sex** with a non-paying male/hijra partner in the last month, how frequently did you use a condom? **(Read out options 1-3)**	Always 1<br>Sometimes 2<br>Never 3<br>No response 98		
305	In the **last one month**, how many different non-paying male partners did you have **oral sex**?	Zero 0<br>Number ………….<br>Don't know/Cannot remember 97<br>No response…….98		

Now let's talk about **non-paying female partner (not Hijras)**

No.	Questions and filters	Coding categories	Skip to	Comments
306	In the **last one-month**, have you had sex **(vaginal/ anal)** with a woman where no payment was involved?	Yes…..1<br>No……2	 →310	
307A	In the **last one month**, how many different female partners have you had sex **(vaginal/ anal)** where no payment was involved?	Number______<br>Don't know/Cannot remember 97<br>No response.98		
307B	In the last **one month**, how many times have you had **vaginal sex** with your non-paying female partners?	Zero 0<br>Number______<br>Don't know/Cannot remember 97<br>No response.98	→308A	
307C	The last time you had **vaginal sex** with a non-paying female partner, did you use a condom?	Yes……1<br>No…..2<br>Don't know/Cannot remember 97<br>No response....98		

No.	Questions and filters	Coding categories	Skip to	Comments
308A	In the last **one month**, how many times have you had **anal sex** with your non-paying female partners?	Zero 0<br>Number______<br>Don't know/Cannot remember 97<br>No response.98	→309	
308B	The last time you had **anal sex** with a non-paying female partner, did you use a condom?	Yes......1<br>No.....2<br>Don't know/Cannot remember 97<br>No response....98		
309	Of all times you had **vaginal or anal sex** with a non-paying female partner in the last month, how frequently did you use a condom?<br>**(Read out options 1-3)**	Always 1<br>Sometimes 2<br>Never 3<br>No response 98		

Now let's talk about **buying sex from women (not hijras)**

No.	Questions and filters	Coding categories	Skip to	Comments
310	In the **last one month**, have you paid any woman (not hijra) to have **(vaginal/anal)** sex with you?	Yes.....1<br>No......2	→315A	
311	In the last one month, how many different women have you paid to have **(vaginal/anal**) sex with you?	Number______<br>Don't know/Cannot remember 97<br>No response.98		
312A	In the **last one month**, how many times did you pay to have **vaginal** sex?	Zero 0<br>Number ....<br>Don't know/Cannot remember 97<br>No response.......98	→312C	
312B	The last time you paid a woman for **vaginal sex**, did you use a condom?	Yes 1<br>No.....2<br>Don't know/Cannot remember 97<br>No response....98		
312C	In the **last one month**, how many times did you pay to have **anal** sex?	Zero 0<br>Number ....<br>Don't know/Cannot remember 97<br>No response.......98	→313	
312D	The last time you paid a woman for **anal sex**, did you use a condom?	Yes 1<br>No.....2<br>Don't know/Cannot remember 97<br>No response....98		
313	Of all times you paid a woman for **vaginal/anal sex** in the **last one month**, how frequently did you use a condom?<br>**(Read out options 1-3)**	Always 1<br>Sometimes 2<br>Never 3<br>No response 98		
314	How would you describe the woman when you last paid for sex?	Street based female sex worker 1<br>Hotel based female sex worker 2<br>Residence based female sex worker 3<br>Brothel based female sex worker 4		

No.	Questions and filters	Coding categories	Skip to	Comments
		Other (specify……………………….) 5<br>Don't know/Cannot remember 97<br>No response….98		

Let's talk about **buying sex from men (not hijra)**

No.	Questions and filters	Coding categories	Skip to	Comments
315A	In the last six months, have you paid another man (not hijra) to have (anal or oral) sex with you?	Yes…..1<br>No……2	→321	
315B	The last time (**in last six months)** you paid for **anal sex** with a man, did you use a condom?	Yes…..1<br>No……2<br>Don't know/Cannot remember 97<br>No response…….98		
315C	In the last one month, have you paid another man (not hijra) to have (anal or oral) sex with you?	Yes…..1<br>No……2	→321	
316	In the **last one month**, how many different men have you paid to have **(anal or oral)** sex with you?	Number______<br>Don't know/Cannot remember 97<br>No response…..98		
317	In the last one month, how many times did you pay to have **anal sex** with men?	Zero 0<br>Number ………….<br>Don't know/Cannot remember 97<br>No response…….98	→320	
318	The last time **in the last one month** you paid for **anal sex** with a man, did you use a condom?	Yes…..1<br>No……2<br>Don't know/Cannot remember 97<br>No response…….98		
319	Of all times you paid for **anal sex** with a man in the last month, how frequently did you use a condom?<br>(**Read out options 1-3**)	Always 1<br>Sometimes 2<br>Never 3<br>No response 98		
320	In the **last one month**, how many different men have you paid to have **oral sex** with you?	Zero 0<br>Number ………….<br>Don't know/Cannot remember 97<br>No response…….98		

Let's talk about **buying sex from hijra**

No.	Questions and filters	Coding categories	Skip to	Comments
321	In the last one month, have you paid a hijra to have **(anal or oral)** sex with you?	Yes…..1<br>No……2	→327	
322	In the **last one-month**, how many different hijra have you paid to have **(anal or oral)** sex with you?	Number ………….<br>Don't know/Cannot remember 97<br>No response…….98		
323A	In the **last one-month**, how many different	Zero 0	→326	

No.	Questions and filters	Coding categories	Skip to	Comments
	hijra did you pay to have **anal sex** with?	Number ....<br>Don't know/Cannot remember 97<br>No response…….98		
323B	In the **last one month**, how many times did you pay to have **anal sex** with hijra?	Zero 0<br>Number ....<br>Don't know/Cannot remember 97<br>No response…….98		
324	The last time you paid a hijra for **anal sex**, did you use a condom?	Yes…..1<br>No……2<br>Don't know/Cannot remember 97<br>No response…….98		
325	Of all times you paid a hijra for anal **sex in the last one month**, how frequently did you use a condom?<br>**(Read out options 1-3)**	Always 1<br>Sometimes 2<br>Never 3<br>No response 98		
326	In the **last one month**, how many different hijra have you paid to have **oral sex** with you?	Zero 0<br>Number ....<br>Don't know/Cannot remember 97<br>No response…….98		

Let's talk about Group Sex

(Vaginal/oral/anal sex with more than 1 partner at the same time)

No.	Questions and filters	Coding categories	Skip to	Comments
327	Have you had sex in a group in the last one month?	Yes…..1<br>No……2<br>Don't know/Cannot remember 97<br>No response…….98	(No, Don't know, No response) →331	
328	Last time you had sex in a group, how many partners (including yourself) were there?	Number ....<br>Don't know/Cannot remember 97<br>No response…….98		
329	Last time you had sex in a group how many of the partners **besides you** used condom?	Number ....<br>Don't know/Cannot remember 97<br>No response…….98		
330	Last time you had sex in a group did you **yourself** use a condom?	Yes…..1<br>No……2<br>Don't know/Cannot remember 97<br>No response…….98		

Section 3B: Let's talk about Mobility (inside country or outside country) & commercial or non-commercial sex

No.	Questions and filters	Coding categories	Skip to	Comments
331	*Have you visited outside this city in the last 12 months (inside country)?*	Yes.....1<br>No......2<br>No response.......98	→336	
332	Have you bought sex in another city in the **last 12 months**?	Yes.....1<br>No......2<br>No response.......98	→334	
333	*Did you use condom in the last sex act while buying sex in another city in the last 12 months?*	Yes.....1<br>No......2<br>No response.......98		
334	Have you had sex in another city in the last ***12 months*** where no payment was made?	Yes.....1<br>No......2<br>No response.......98	→336	
335	Did you *use condom in the last non-commercial sex act in another city in the last 12 months?*	Yes.....1<br>No......2<br>No response.......98		
336	Have you been to any other country outside Bangladesh during the last *12 months*?	Yes.....1<br>No......2<br>No response.......98	→401	
336 A	If you have visited to other country than Bangladesh- which country was that?	1 ....<br>2.........................<br>3.........................		
337	Have you bought sex in another country in the **last 12 months**?	Yes.....1<br>No......2<br>No response.......98	→339	
338	Did you use condom during your last sex act while buying sex in another country in the last **12 months**?	Yes.....1<br>No......2<br>No response.......98		
339	Have you had sex in another country in the last **12 months** where no payment was made?	Yes.....1<br>No......2<br>No response.......98	→401	
340	Did you *use condom during your last non-commercial sex act in another country in the* last **12 months**?	Yes.....1<br>No......2<br>No response.......98		

Section 4: Let's talk about Male Condoms and Lubricants

No.	Questions and filters	Coding categories	Skip to	Comments
401	**SHOW CONDOM and ask**<br>"Can you tell me what this is"?	Can identify as condom 1<br>Cannot identify as condom 2<br>No response 98	(2, 98) →406	
402	Do you have a condom with you now? Please show me	Can show a condom…1<br>Cannot show a condom 2<br>No response 98		
403	Which places or persons have you obtained condoms from in the last one month?<br><br>(Multiple answers possible)<br><br>(Do not read out)<br>(Circle 1 if mentioned)<br>(Circle 2 if not mentioned)	Don't know where condom available 1 2<br>Shop….. 1 2<br>Pharmacy….. 1 2<br>DIC------1 2<br>Depot holder 1 2<br>Health facility (Excluding DIC)…… 1 2<br>Bar/Guest House/Hotel….. 1 2<br>Friends…… 1 2<br>NGO worker….. 1 2<br>Did not buy condom in last month-- 1 2<br>Never used condom 1 2<br>Did not get condom 1 2<br>Other-------------------------------- 1 2<br>No response…..98	→ if 1, then go to q406	
404	Can you obtain a condom every time you need one?	Yes…..1<br>No……2<br>Don't need one…3<br>Never used condom 4<br>Don't know/Cannot remember 97<br>No response…….98	→406<br>→405<br>(3, 4, 97, 98) →406	
405	Why can't you get a condom every time you need one?<br><br>(Multiple answers possible)<br>(Do not read out)<br>(Circle 1 if mentioned)<br>(Circle 2 if not mentioned)	DIC is far away 1 2<br>Peer educator not available when needed 1 2<br>Cost too much…..1 2<br>Shop/pharmacy too far away……1 2<br>Shops pharmacy closed……1 2<br>Shy to buy condom…….1 2<br>Don't know where to obtain…….1 2<br>Don't want to carry them ……1 2<br>No need------1 2<br>Never use in life-----1 2<br>Other___________________1 2<br>Don't know/Cannot remember 97<br>No response……….98		
406	Have you ever used lubricant when having	Yes….. 1		

No.	Questions and filters	Coding categories	Skip to	Comments
	anal sex?<br>**I mean something to make your own or your partner's penis slippery so it is easier to have anal sex**	No...... 2<br>Don't know/Cannot remember 97<br>No response....... 98	→409	
407	What lubricant did you use during last anal sex?<br><br>(Multiple responses possible)<br>(Do Not Read Out)	Saliva......1 2<br>Oil........1 2<br>Water-based condom lubricant.......1 2<br>Antiseptic cream.......1 2<br>Normal lotion........1 2<br>Vaseline/Petroleum Jelly/Beauty Cream......1 2<br>Shampoo/Soap......1 2<br>Other______________1 2<br>Don't know/Cannot remember 97<br>Not response............98		
408	Were you using a condom that time?	Yes.........1<br>No..............2<br>Don't know/Cannot remember 97<br>No response... 98		
409	Some people use a lubricant product made especially for use with condoms. Have you heard of such a product?	Yes.....1<br>No......2<br>Don't know/Cannot remember 97<br>No response.......98	 →414<br>→414<br>→414	
410	Can you tell me the brand name of such a product?	Yes.....1<br>Record Name __________________<br>No......2<br>Don't know/Cannot remember 97<br>No response.......98		
411	In the last one month, how frequently have you used a special lubricant for condoms together with a condom during anal sex?	Always.........1<br>Sometimes.........2<br>Never 3<br>Don't know/Cannot remember 97<br>No response........98<br>No sex in last month........99	→413<br>→412<br>→412<br>→414<br>→414<br>→501	
412	Why do you **sometimes** not use special condom lubricant, or **never** use it?<br><br>(Multiple answers possible)<br>(Do not read out)<br>(Circle 1 if mentioned)<br>(Circle 2 if not mentioned)	Costs too much.......1 2<br>Shy to buy lubricant.......1 2<br>Don't know where to obtain......1 2<br>I do not need to use.......1 2<br>I use other cream.......1 2<br>Supply short......1 2<br>Not easy to carry.......1 2<br>Other___________________1 2<br>Don't know/Cannot remember 97<br>No response.......98	→414<br><br>→414<br>→414	
413	For you, what are the purposes of using	Decrease pain/inflammation..1 2		

No.	Questions and filters	Coding categories	Skip to	Comments
	special condom lubricant with condoms during sex **always**?<br><br>(Multiple answers possible)<br>(Do not read out)<br>(Circle 1 if mentioned)<br>(Circle 2 if not mentioned)	Increase feeling..........1 2<br>Decrease risk of condom breakage..1 2<br>Prevent STI/HIV/AIDS infection.....1 2<br>Other ____________________1 2<br>Don't know/Cannot remember 97<br>No response........98		
414	In the last one month, did you have a breaking of condom while having anal sex?	Yes.........1<br>No..............2<br>Don't know... 97<br>No condom use in last month...3<br>Never use condom in life...4<br>No response... 98<br>No sex in last month...99	→501<br>→501<br>→501<br>→501	
415	Sources of condom in the last one month?<br><br>(Multiple answers possible)<br>(Do not read out)<br>(Circle 1 if mentioned)<br>(Circle 2 if not mentioned)	Shop..... 1 2<br>Pharmacy..... 1 2<br>DIC......1 2<br>Depot holder......1 2<br>Bar/Guest House/Hotel..... 1 2<br>Friends...... 1 2<br>Broker (Dalal).... 1 2<br>NGO worker..... 1 2<br>Bought condom in last month.....1 2<br>Sex partner......1 2<br>Other-------------------------------- 1 2<br>Don't know/Cannot remember 97<br>No response.......98		

Section 5: Let's talk about STDs

No.	Questions and filters	Coding categories	Skip to	Comments
501	Could you describe any symptoms in men of diseases that can be transmitted by having sex?<br><br>(DO NOT READ OUT)<br>(Circle 1 when mentioned)<br>(Circle 2 when not mentioned)<br>(Multiple responses possible)	Penis discharge 1 2<br>Burning pain on urination 1 2<br>Genital ulcers/sores 1 2<br>Swellings in groin area 1 2<br>Anal discharge 1 2<br>Anal ulcer/sores 1 2<br>Other: .... 1 2<br>Don't know/Cannot remember 97<br>No response 98		
502	Have you had a urethral discharge during the last 12 months? (Something liquid and sticky but not semen)	Yes 1<br>No 2<br>Don't know/Cannot remember 97<br>No Response 98		
503	Have you had anal discharge during the last 12 months? (Something liquid and sticky)	Yes 1<br>No 2		

No.	Questions and filters	Coding categories	Skip to	Comments
		Don't know/Cannot remember 97<br>No Response 98		
504	Have you had a genital ulcer / sore during the last 12 months?	Yes 1<br>No 2<br>Don't know/Cannot remember 97<br>No response 98		
505	Had genital ulcer / discharge / sore (penis and or anal) during the last 12 months?<br>**If yes in any of Q502, 503, 504- then circle 1 of 505 otherwise circle 2.**	Yes 1<br>No 2	→509	
506A	What was the first thing you did when you had those symptoms in last time in the last 12 months?<br><br>(DO NOT READ OUT)<br>(Only one response)	Treatment from hospital……1<br>Treatment from drug seller……2<br>Treatment from private doctor……3<br>Treatment from private clinic……4<br>Treatment from NGO clinic ……5<br>Treatment from traditional healer……6<br>Advice/treatment from friend……7<br>Self treatment…… 8<br>Nothing ……… 9<br>Other__________________10<br>Don't know/Cannot remember 97<br>No Response 98	→Ask 506B<br><br>→509	
506B	Name of NGO clinic?			
507	Last time you had one of those symptoms that you just told me about, how many days did you wait between discovering symptoms and going for treatment	Within a day………0<br>Number of days____________<br>Don't know/Cannot remember 97<br>No response 98		
508	Last time you had those symptoms, how much did the treatment cost you, including the medicine and the fees for the service?	Tk_________<br>Don't know/Cannot remember 97<br>No response 98		
509	Do you yourself do anything to avoid getting diseases which are transmitted by sex?<br><br>(Multiple answers possible)<br>(DO NOT READ OUT)<br>(Circle 1 if mentioned)<br>(Circle 2 if not mentioned)	Nothing….1 2<br>Wash genitals with dettol or urine after sexual intercourse…. 1 2<br>Always use condoms 1 2<br>Sometimes use condom 1 2<br>Always trusted sex partners 1 2<br>Sex partners test before sex/sex with neat clean partners…. 1 2<br>Other__________________1 2<br>Take medicine……1 2<br>Don't know/Cannot remember 97<br>No response 98	→511 (if "1")<br><br>→ask 510	
510	What medicine do you take?	Name________________<br>Don't know/Cannot remember 97		

No.	Questions and filters	Coding categories	Skip to	Comments
		No response 98		
511	During the last month have you visited an NGO STI clinic that is working with men who have sex with men in this city?	Yes 1<br>No 2<br>Don't know/Cannot remember 97<br>No response 98	→ 601 (No, Don't know/Cannot remember, No response)	
512	If yes, which clinic was it?<br>(Multiple answer possible)	Name(s) of the clinic<br>1. ....<br>2 ....<br>3		

Section 6: Let's talk about AIDS knowledge, risk and avoidance

No.	Questions and filters	Coding categories	Skip to	Comments
601	Have you ever heard of HIV or the disease called AIDS?	Yes 1<br>No 2<br>No response 98	→ 701	
602	Can people reduce their risk of HIV by using a condom correctly every time they have sex?	Yes 1<br>No 2<br>Don't know/Cannot remember 97<br>No response 98		
603	Can people reduce their risk of HIV by avoiding anal sex?	Yes 1<br>No 2<br>Don't know/Cannot remember 97<br>No response 98		
604	Can people reduce their risk of HIV by using a condom correctly every time they have vaginal/anal sex?	Yes 1<br>No 2<br>Don't know/Cannot remember 97<br>No response 98		
605	Can people reduce their risk of HIV by avoiding multiple sexual partners?	Yes 1<br>No 2<br>Don't know/Cannot remember 97<br>No response 98		
606	Can a person get the HIV from mosquito bites?	Yes 1<br>No 2<br>Don't know/Cannot remember 97<br>No response 98		
607	Can a person get HIV by sharing a meal with someone who is infected?	Yes 1<br>No 2<br>Don't know/Cannot remember 97<br>No response 98		
608	Can a person get HIV by taking injections with a needle that has already been used by someone else?	Yes 1<br>No 2<br>Don't know/Cannot remember 97<br>No response 98		
609	Do you think you can tell by looking at	Yes 1		

No.	Questions and filters	Coding categories	Skip to	Comments
	someone whether they are infected with HIV?	No 2<br>Don't know/Cannot remember 97<br>No response 98		
610	What do you yourself do to avoid getting HIV?<br><br>(Multiple responses possible)<br><br>(Do not read out)<br>(Circle 1 if mentioned)<br>(Circle 2 if not mentioned)	Nothing….1 2<br>Wash genitals with dettol or urine after sexual intercourse ………1 2<br>Always use condoms ….. 1 2<br>Sometimes use condom…. 1 2<br>Take medicine…… 1 2<br>Always sex with trusted partner…… 1 2<br>Sex partner check before sex/sex with clean partners …… 1 2<br>Other___________________1 2<br>Don't know/Cannot remember 97<br>No response 98	→ 611 (if "1")	

Confidential test for HIV
(Confidential means that no one will know the result if you don't want them to know)

No.	Questions and filters	Coding categories	Skip to	Comments
611	Do you know anywhere you could go if you wanted to get a confidential test to find out if you are infected with HIV?	Yes 1<br>No 2<br>Don't know/Cannot remember 97<br>No response 98	→ 701	
612	I don't want to know the result, but have you ever had an HIV test?	Yes 1<br>No 2<br>Don't know/Cannot remember 97<br>No response……. 98	→ 701	
613	If yes, where the HIV test was conducted last time?			
614	Did you yourself request the test or did someone offer you to test for HIV, or were you required to have the test?	Self 1<br>Someone offered 2<br>Required 3<br>No response 98	→ ask 617<br>→ ask 615<br>→ ask 616<br>→ ask 617	
615	Who request to have HIV test? **(only 1 response)**			
616	Why the HIV test was required? **(only 1 response)**			
617	Please do not tell me the result, but did you get the result of your test?	Yes 1<br>No 2<br>Don't know/Cannot remember 97<br>No response 98		
618	When did you have the most recent HIV test?	Within last six months 0<br>More than six months to one year ……1		

No.	Questions and filters	Coding categories	Skip to	Comments
		More than a year ago ....2<br>Don't know/Cannot remember 97<br>No response 98		

Section 7: Let's Talk about Violence

No.	Questions and filters	Coding categories	Skip to	Comments
701	In the last 12 months, were you ever beaten?	Yes 1<br>No 2<br>Don't know/Cannot remember 97<br>No response 98	 → 703<br>→ 703<br>→ 703	
702	If yes, who was the person beat you?<br><br>(DO NOT READ OUT)<br>(Multiple answer possible)<br>(Circle 1 if mentioned)<br>(Circle 2 if not mentioned)	Men in uniform..... 1 2<br>Mastans...... 1 2<br>New sex partner..... 1 2<br>Regular sex partner.... 1 2<br>Local people .... 1 2<br>Family/Relatives .... 1 2<br>Others_____________ 1 2<br>Don't know/Cannot remember 97<br>No responses.... 98		
703	In the last 12 months, were you physically forced to have sex with someone even though you did not want to?	Yes 1<br>No 2<br>Don't know/Cannot remember 97<br>No response 98	 → 705<br>→ 705<br>→ 705	
704	Who was the person (or people) physically forced you to have sex against your will?<br><br>**(**Multiple answers possible**)**<br>(DO NOT READ OUT)<br>(Circle 1 if mentioned)<br>(Circle 2 if not mentioned)	Men in uniform..... 1 2<br>Mastans...... 1 2<br>New sex partner..... 1 2<br>Regular sex partner.... 1 2<br>Local people .... 1 2<br>Relatives .... 1 2<br>Others_____________ 1 2<br>Don't know/Cannot remember 97<br>No responses.... 98		
705	During the last 12 months, have you been arrested?	Yes 1<br>No 2<br>Don't know/Cannot remember 97<br>No response 98	(No, 97, 98) → 801	
706	If yes, what was the reason for arrest?	1. ....<br>2. ....<br>3.		

Section 8: Risk Perceptions

No.	Questions and filters	Coding categories	Skip to	Comments
801	Do you think that you are at high risk, some risk or little or no risk for HIV?	High risk........1<br>Some risk........2<br>Little risk........3<br>No risk........4<br>Don't know/Cannot remember 97<br>No response......98	→ 802<br>→ 802<br>→ 803<br>→ 803<br>→ 901<br>→ 901	
802	Why do you think you are at **high or some** risk for HIV?<br><br>**(**Multiple answers possible**)**<br>(DO NOT READ OUT)<br>(Circle 1 if mentioned)<br>(Circle 2 if not mentioned)	Risky behaviour .... 1 2<br>Frequent anal sex .... 1 2<br>Don't use condoms........ 1 2<br>Irregular condom use..... 1 2<br>Shared needles/syringes........... 1 2<br>Other-------------------------------- 1 2<br>Don't know/Cannot remember 97<br>No response......98		
803	Why do you think you are at **little or no risk** of HIV?<br><br>**(**Multiple answers possible**)**<br>(DO NOT READ OUT)<br>(Circle 1 if mentioned)<br>(Circle 2 if not mentioned)	Always use condoms ....1 2<br>Partners are clean ....1 2<br>Partners are healthy.......1 2<br>Never share needles/syringes 1 2<br>Sometimes share needles/syringes 1 2<br>Irregular use of condom 1 2<br>Always have sex with single trusted partner 1 2<br>Always be neat and clean 1 2<br>Less frequent sex 1 2<br>Wash after sex work 1 2<br>Other____________________1 2<br>Don't know/Cannot remember 97<br>No response 98		

Section 9: Exposure to interventions

No.	Questions and filters	Coding categories	Skip to	Comments
901	Have you **ever** participated in any NGO/Self help group/CBO - run AIDS prevention program?	Yes 1<br>No 2<br>No NGO/Self help group/CBO in this area 96<br>Don't know/Cannot remember 97<br>No response 98	(No, 96, 97, 98) → 907	
902	How long have you been involved in the AIDS prevention program?	.... months<br>Within in a month 00<br>Don't know/Cannot remember 97<br>No response 98		
903	When did you last participate in any AIDS prevention program?	.... months<br>Within in a month 00<br>Don't know/Cannot remember 97		

No.	Questions and filters	Coding categories	Skip to	Comments
		No response 98		
904	How many times did you participate in any AIDS prevention program in the **last month**?	Zero 0<br>Number .....<br>Don't know/Cannot remember 97<br>No response 98		
905A	In last 1 month,which type of activities have you participated?<br><br>(Do not read out)<br>(Multiple answers possible)<br>(Circle 1 if mentioned<br>(Circle 2 if not mentioned	Needle exchange program 1 2<br>Education 1 2<br>Received condoms 1 2<br>Received lubricants 1 2<br>Treatment for STD 1 2<br>Treatment for general health 1 2<br>Attending IHC 1 2<br>Attending DIC 1 2<br>Attending VCT 1 2<br>Others .... 1 2<br>Don't know/Cannot remember 97<br>No Response 98		
905B	In your whole life which type of activities have you participated?<br><br>(Do not read out)<br>(Multiple answers possible)<br>(Circle 1 if mentioned<br>(Circle 2 if not mentioned	Needle exchange program 1 2<br>Education 1 2<br>Received condoms 1 2<br>Received lubricants 1 2<br>Treatment for STD 1 2<br>Treatment for general health 1 2<br>Attending IHC 1 2<br>Attending DIC 1 2<br>Attending VCT 1 2<br>Others .... 1 2<br>Don't know/Cannot remember 97<br>No Response 98		
906	In your whole life, how did you benefit from the sessions?<br><br>(Multiple answers possible)<br><br>(DO NOT READ OUT)<br>(Circle 1 if mentioned)<br>(Circle 2 if not mentioned)	Helped you change your behaviour 1 2<br>Gave useful information but did not affect behaviour 1 2<br>Learnt about HIV/AIDS/STD/safe sex and correct use of condom 1 2<br>Information was not easily understandable 1 2<br>Was not relevant to our needs....... 1 2<br>Other ----------------------------------- 1 2		

Section 10: Meeting place and Miscellaneous

No.	Questions and filters	Coding categories	Skip to	Comments
907	Where do you usually meet your friends?<br><br>(Multiple answers possible)<br><br>(DO NOT READ OUT)<br>(Circle 1 if mentioned)<br>(Circle 2 if not mentioned)	Cruising spot 1 2<br>Residence 1 2<br>Club 1 2<br>Party 1 2<br>Tea stall 1 2<br>On the street 1 2<br>Bazaar/Market 1 2<br>Hotel/Boarding 1 2<br>Working place 1 2<br>School/College/Madrasha 1 2<br>Other...................................... 1 2<br>Don't know/Cannot remember 97<br>No response 98		
908	How do you contact your male sex partner for having sex?<br><br>(Multiple answers possible)<br><br>(DO NOT READ OUT)<br>(Circle 1 if mentioned)<br>(Circle 2 if not mentioned)	Cruising spot 1 2<br>Phone 1 2<br>Internet 1 2<br>Through friends 1 2<br>Pimp 1 2<br>In clubs 1 2<br>In parties 1 2<br>Tea stall 1 2<br>On the street 1 2<br>Bazaar/Market 1 2<br>Hotel/Boarding 1 2<br>Working place 1 2<br>School/College/Madrasha 1 2<br>At home 1 2<br>Other... 1 2<br>Don't know/Cannot remember 97<br>No response 98		
909	With regards to your sexual behaviour, how would you identify yourself?<br><br>(DO NOT READ OUT)<br><br>(Only one response)	Man/manly/general people 1<br>Parikh 2<br>Film hero 3<br>Panthi 4<br><br>Film heroin 5<br>Gay 6<br>Koti 7<br>Do-parata 8<br>Girls/woman 9<br>Other: .... 10<br>Don't know/Cannot remember 97<br>No response 98	(1–4) → Stop the interview with thanks<br><br>(5–9) → 910	
910	Have you ever taken any medicine to adopt feminine trait?	Yes 1<br>No 2<br>Don't know/Cannot remember 97<br>No response 98	(2, 97, 98) → Stop interview with thanks	
911	If yes, have you taken in the last 3 months?	Yes 1<br>No 2<br>Don't know/Cannot remember 97<br>No response 98	(2, 97, 98) → Stop interview with thanks	

No.	Questions and filters	Coding categories	Skip to	Comments
912	Mention the name of medicine which was taken in the last 3 months?<br><br>(Multiple answers possible)<br><br>(DO NOT READ OUT)<br>(Circle 1 if mentioned)<br>(Circle 2 if not mentioned)	Shukhi 1 2<br>Cilest-21 1 2<br>Desolon 1 2<br>Lynes 1 2<br>Marvelon 1 2<br>Ovostat 1 2<br>Femecon 1 2<br>Nordat-28 1 2<br>Emcon 1 2<br>Postinor-2 1 2<br>Minicon 1 2<br>Ovacon 1 2<br>Others ……… 1 2<br>Don't know/Cannot remember 97<br>No response 98		
913	Mention the reasons for taking medicine in the last 3 months?<br><br>(Multiple answers possible)<br>(DO NOT READ OUT)<br>(Circle 1 if mentioned)<br>(Circle 2 if not mentioned)	*Increased breast size 1 2*<br>*Increased shape of thigh/hip 1 2*<br>*Increased smoothness of body 1 2*<br>To prevent moustache 1 2<br>Others ……… 1 2<br>Don't know/Cannot remember 97<br>No response 98		
914	How frequently have you taken that medicine in the last 3 months?<br><br>(DO NOT READ OUT)	Every day 1<br>More than once in a week 2<br>Once in a week 3<br>Once in a month 4<br>More than once in a month 5<br>Don't know/Cannot remember 97<br>No response 98		

Thank you very much for your kind cooperation and spending your valuable time with me.

= ✜ =
